# Synthesis and Antimycobacterial Evaluation of *N*-(4-(Benzyloxy)benzyl)-4-aminoquinolines

**DOI:** 10.3390/molecules27082556

**Published:** 2022-04-15

**Authors:** Estevão Silveira Grams, Alessandro Silva Ramos, Mauro Neves Muniz, Raoní S. Rambo, Marcia Alberton Perelló, Nathalia Sperotto, Laura Calle González, Lovaine Silva Duarte, Luiza Galina, Adilio Silva Dadda, Guilherme Arraché Gonçalves, Cristiano Valim Bizarro, Luiz Augusto Basso, Pablo Machado

**Affiliations:** 1 Instituto Nacional de Ciência e Tecnologia em Tuberculose, Centro de Pesquisas em Biologia Molecular e Funcional, Pontifícia Universidade Católica do Rio Grande do Sul, Porto Alegre 90619-900, RS, Brazil; grams.est@gmail.com (E.S.G.); alessandro.ramos@acad.pucrs.br (A.S.R.); mauro.neves.muniz@gmail.com (M.N.M.); raoni.rambo@gmail.com (R.S.R.); marcia.perello@pucrs.br (M.A.P.); nathalia.sperotto@gmail.com (N.S.); laura.gonzalez@edu.pucrs.br (L.C.G.); lovaine.duarte@pucrs.br (L.S.D.); luiza.galina@acad.pucrs.br (L.G.); adiliosd@yahoo.com.br (A.S.D.); arracheg@gmail.com (G.A.G.); cristiano.bizarro@pucrs.br (C.V.B.); luiz.basso@pucrs.br (L.A.B.); 2 Programa de Pós-Graduação em Biologia Celular e Molecular, Pontifícia Universidade Católica do Rio Grande do Sul, Porto Alegre 90619-900, RS, Brazil; 3 Programa de Pós-Graduação em Medicina e Ciências da Saúde, Pontifícia Universidade Católica do Rio Grande do Sul, Porto Alegre 90616-900, RS, Brazil

**Keywords:** *Mycobacterium tuberculosis*, drug discovery, synthesis, quinolines, tuberculosis

## Abstract

Tuberculosis remains a global health problem that affects millions of people around the world. Despite recent efforts in drug development, new alternatives are required. Herein, a series of 27 *N*-(4-(benzyloxy)benzyl)-4-aminoquinolines were synthesized and evaluated for their ability to inhibit the *M. tuberculosis* H37Rv strain. Two of these compounds exhibited minimal inhibitory concentrations (MICs) similar to the first-line drug isoniazid. In addition, these hit compounds were selective for the bacillus with no significant change in viability of Vero and HepG2 cells. Finally, chemical stability, permeability and metabolic stability were also evaluated. The obtained data show that the molecular hits can be optimized aiming at the development of drug candidates for tuberculosis treatment.

## 1. Introduction

Tuberculosis (TB) is an infectious disease caused by *Mycobacterium tuberculosis* (Mtb), its main etiologic agent in humans. Despite the negative impact on the number of diagnoses and notifications caused by the COVID-19 pandemic, the numbers of cases and deaths caused by tuberculosis remain alarming. According to the World Health Organization (WHO), in 2020, there were 5.8 million cases with 1.8 million deaths worldwide [1]. The WHO also warns that underreporting and lack of adequate treatment, due to a health system overloaded by the pandemic, could reverse years of advances made in reducing the impacts of TB. The Mtb mainly affects the respiratory tract of the infected individual which may expel droplets containing the bacillus. The main symptoms are a persistent cough, hemoptysis, weight loss, fever or night sweats, chest pain, and permanent pneumonia [2]. The recommended treatment for disease indicates a therapeutic regimen consisting of 2 months of isoniazid (INH), ethambutol, rifampicin, and pyrazinamide, and 4 months of isoniazid and rifampicin [3]. Although this regimen has some efficacy, the emergence of drug-resistant Mtb strains has made clinical management difficult and is currently one of the main difficulties in reducing mortality and disease prevalence [4]. Among the first-line drugs, rifampicin was the last to come into clinical use at the end of the 1960s [5]. Only more than 40 years later, a new drug was incorporated into the pharmacological alternatives to combat the disease. Bedaquiline [6] was approved in 2012 for the treatment of tuberculosis caused by Mtb-resistant strains. Subsequently, two new drugs were also approved for the treatment of patients infected with resistant strains to clinically available drugs—delamanid [7] and pretomanid [8], approved in 2014 and 2019, respectively.

Within this context, our research group has studied new compounds capable of inhibiting the growth of drug-susceptible and drug-resistant Mtb strains with some encouraging results [9,10,11,12]. Among the chemical classes evaluated in our laboratories, 4-aminoquinolines have shown selective activities against the bacillus using in vitro experiments [13]. Therefore, following our ongoing program for new compounds endowed with potent activities against Mtb and in an attempt to advance the understanding of the structure–activity relationship (SAR) of this class of molecules, we synthesized a series of *N*-(4-(benzyloxy)benzyl)-4-aminoquinolines for further evaluation as possible inhibitors of Mtb growth. To the best of our knowledge, the synthesis and antimycobacterial activity of 4-aminoquinolines containing the benzyloxybenzyl system have not yet been described. This bulky group can assume different conformations with significant impact on lipophilicity. It is important to mention that substituents attached at quinoline ring and the benzyloxybenzyl group were chosen among halogens, alkyl or methoxyl groups in order to obtain the basic structural requirements for activity of the synthesized structures (SAR). Furthermore, the viability of Vero and HepG2 cells was used as measure of the selectivity of most active molecules (selective toxicity). Finally, chemical stability, permeability, and metabolic stability of these structures were also determined in a preliminary ADME in vitro profiling. 

## 2. Results and Discussion

**Scheme 1:**
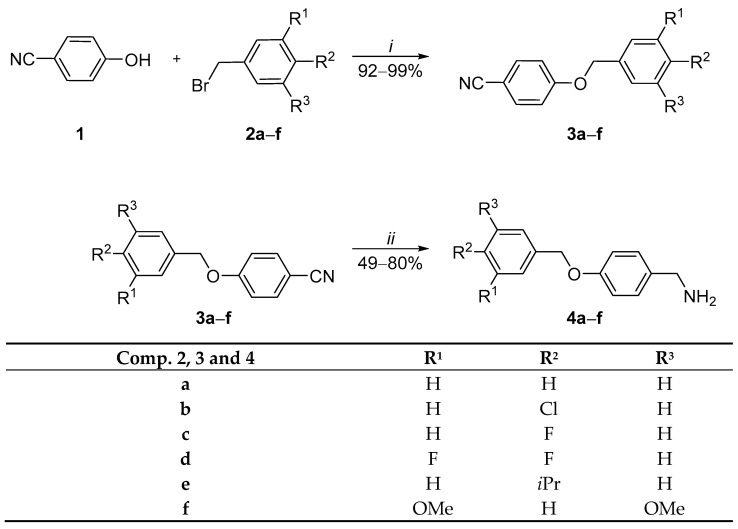
Reagents and conditions: *i* = K_2_CO_3_, acetone, 56 °C, 4 h; *ii* = (1) Li(AlH_4_), THF, 0–25 °C, 16 h, (2) H_2_O, NaOH, 12 h.

**Scheme 2:**
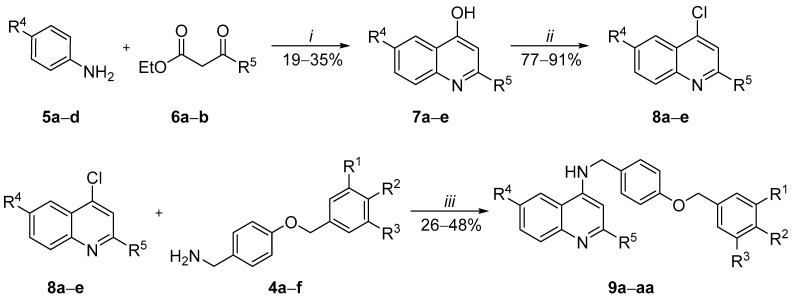
Reagents and conditions: *i* = (1) Na_2_SO_4_, AcOH, EtOH, 80 °C, 16 h, (2) Dowtherm^®^ A, 230–250 °C, 15 min; *ii* = POCl_3_, toluene, 110 °C, 2 h; *iii* = DIPEA, DMSO, 150 °C, 20 h.

The synthesis of the compounds of interest involved five reaction steps (Scheme 1 and Scheme 2). The first series of synthesized molecules were obtained under second-order nucleophilic substitution reaction conditions (S_N_2) from 4-cyanophenol (1) and benzyl bromides **2a**–**f**. The reaction was carried out using acetone under reflux in the presence of potassium carbonate (K_2_CO_3_) providing the 4-(benzyloxy)benzonitriles **3a**–**f** with 92–99% yields (Scheme 1). The second step was related to the reduction reaction of the nitrile group using lithium aluminum hydride (Li(AlH_4_)). This reaction was performed in two steps under one-pot conditions. Using THF as solvent at 0 °C, the nitrile group suffered a nucleophilic addition of Li(AlH_4_) with formation of corresponding imine. The reaction was left for 16 h under stirring at 25 °C. The second step involved the hydrolysis of the intermediate structure formed by adding water in a basic medium (NaOH) with additional stirring for 12 h. The 4-(benzyloxy)benzylamines (**4a**–**f**) were obtained in 49–80% yields under these experimental conditions and were used in the subsequent reaction without further purification procedure (Scheme 1). The electronic parameters of the substituents do not seem to have any influence on the conversions and formations of the products. Regardless of the electron-donating or -withdrawing characteristic of the substituents, the yields varied without a defined pattern. Thus, the observed yield variations must be related to the isolation procedures rather than electronic effects in obtaining amines **4a**–**f**.

The synthesis of 2-alkyl-4-hydroxyquinolines **7a**–**e** was carried out from the classical Conrad–Limpach reaction between anilines (**5**) and *β*-ketoesters (**6**) in two steps. The first step was accomplished in the presence of magnesium sulfate and acetic acid in ethanol to provide the *β*-acrilate intermediate. Addition-elimination occurs at the more electrophilic ketone carbonyl forming the *β*-acrilate. These intermediates were not isolated or characterized and were reacted in the subsequent one-pot step. This next step was performed using Dowtherm^®^ A as the heating transfer fluid. The thermal cyclization of the intermediates with the concomitant formation of heterocycles occurred at temperatures of 230–250 °C for 15 min [11]. 2-Alkyl-4-hydroxyquinolines **7a**–**e** were obtained in 19–35% yields (Scheme 2).

The synthesis of 2-alkyl-4-chloroquinolines **8a**–**e** was carried out in the presence of phosphorus (V) oxychloride (POCl_3_) under refluxing toluene for 2 h [14]. The products were obtained with yields of 77–91% (Scheme 2). Regardless of the electronic characteristics of the substituents attached at the 6-position of the heterocyclic ring (R^4^), the yields were similar. In addition, methyl or ethyl at the 2-position of the quinoline (R^5^) also did not significantly alter the product yields.

The last synthetic step was performed under nucleophilic aromatic substitution conditions between 2-alkyl-4-chloroquinolines (**8**) and 4-(benzyloxy)benzylamines (**4**). The 4-amino-*N*-(4-benzyloxy)quinolines **9a**–**aa** were obtained in the presence of *N*,*N*-diisopropylethylamine (DIPEA) as base and dimethylsulfoxide (DMSO) as solvent. The reaction was carried out at 150 °C for 20 h. The 4-amino-*N*-(4-benzyloxy)quinolines **9a**–**aa** were obtained in 26–48% yields after purification on silica gel column chromatography (Scheme 2). The observed yields seem to be related to the efficiency of the purification process, as no differences were obtained in the chemical conversions linked to the different substituents used in the reactions. Spectroscopic and spectrometric data were found to be in total agreement with the proposed structures (Supplementary Materials).

The synthesized compounds **9a**–**aa** were evaluated for their ability to inhibit the growth of the *M. tuberculosis* H37Rv strain [10]. The first-line drug INH was used as positive control in the experiments. In general, unsubstituted molecules at the 6-position of the quinoline ring (R^4^) showed the lowest ability to inhibit the growth of the bacillus (Table 1). A relative potency improvement was observed in the presence of the methoxy group attached at the 6-position of heterocycle (Table 1). Finally, the most effective compounds of the synthesized series presented the chlorine and bromine attached at the 6-position (Table 1). Indeed, the absence of substituent at the 6-position of quinolines **9a**–**f** produced inactive compounds at the highest concentrations evaluated (**9a** and **9b**) and some molecules with minimal inhibitory concentrations (MIC) ranging from 12.1 to 26.8 µM. Interestingly, the presence of the chlorine atom at the 6-position of the heterocyclic ring lead to structures with similar activities independent of the substituents in the benzyloxy moiety (R^1^, R^2^, and R^3^). Compounds **9g**–**l** exhibited MICs ranging from 5.7 to 6.4 µM. It is also important to mention that these quinolines showed more than one unit of variation in CLogP values, indicating that liposolubility does not seem positively correlated with the antimycobacterial activity of this chemical class. The classical bioisosteric exchange between chlorine and bromine lead to the most effective compounds in the series of synthesized molecules. Quinolines **9m**–**t** exhibited MICs values ranging from 2.7 to 5.8 µM. In the benzyloxy group, the presence of chlorine (**9n**) and fluorine (**9o**) halogens attached at the 4-position of the benzene ring resulted in more potent molecules. Compounds **9n** and **9o** presented MIC values of 2.7 and 2.8 µM, respectively. The absence of these atoms in the six-membered ring reduced the activity by more than 2-fold as quinoline **9m** (unsubstituted) showed a MIC of 5.8 µM. Additionally, the presence of one more fluorine atom at the 3-position of the benzene ring also reduced the inhibition capacity on the bacillus as difluorinated-compounds **9p** exhibited a MIC of 5.3 µM. Finally, the presence of a bulky and electron-donating group such as iso-propyl group in **9q** was also unable to maintain the antimycobacterial activity at the same levels as benzyloxy-halogenated molecules **9n** and **9o**. Furthermore, the 2-position (R^5^) of the heterocyclic ring was also studied. In this position, the presence of an ethyl in **9t** also reduced the activity of the structure when compared to its methylated analogue **9n**. Quinoline **9t** exhibited a MIC of 5.2 µM, which was 1.9-fold higher than MIC value showed by **9n**. Considering the presence of the methoxy group at the 6-position of quinolines **9u**–**9aa**, the inhibition potency presented was intermediate and inferior compared to that presented by halogenated compounds. When attached at the 6-position, this electron-donating group produced compounds with MIC values ranging from 5.9 to 23.4 µM.

Although quinoline derivatives have inhibited the Mtb growth targeting *Mycobacterium tuberculosis* enoyl-acyl carrier protein reductase [13] and cytochrome *bc_1_* complex [9], the molecular mechanism of action of *N*-(4-(benzyloxy)benzyl)-4-aminoquinolines is still unknown and further studies are needed to clarify this point.

Finally, it is noteworthy that first-line drug INH, when evaluated under the same experimental conditions, presented a MIC of 2.3 µM. This value was close to that observed for quinolines **9n** and **9o**, which prompted us to continue subsequent studies with these two compounds.

The most effective compounds (**9n** and **9o**) were evaluated for selectivity using Vero and HepG2 cells. In these experiments, two methods of viability assessment were used; MTT to determine mitochondrial activity [15] and neutral red to assess lysosomal viability [16]. Exposed for 72 h at concentrations similar to MIC presented by quinolines **9n** and **9o**, cell viability was not significantly altered, indicating a certain selectivity in the inhibition of Mtb versus mammalian cells (Table 2). When placed in media containing different pHs for 24 h, the compounds **9n** and **9o** showed low stability, with remaining concentrations ranging from 5 to 22.9% (Table 2). The passive permeability of the molecules was evaluated by the parallel artificial membrane permeability assay (PAMPA) [17] (Table 2). The results showed that the chlorinated structure **9n** has low permeability while fluorinated derivative **9o** showed high permeability under the conditions studied. This finding denotes that minor structural change can drastically alter the permeability parameters of this class of compounds. Finally, metabolism in the presence of rat microsomes also showed different results for quinolines **9n** and **9o** (Table 2). While compound **9n** exhibited moderate metabolism rates (47 ≤ Cl_int_ ≥ 16) [18], fluorinated analogue **9o** showed high metabolism rates (Cl_int_ > 47) [18]. The rate of metabolism showed significant effect on difference in half-life presented by the molecules. Compound **9n** had half-life of 19 min while this parameter for molecule **9o** was only 7.9 min.

Taking together, the data described herein indicate that we have developed a new series of compounds containing the quinoline nucleus using accessible reactants and reagents in straightforward chemical steps. Among the synthesized structures, some molecules were able to inhibit the growth of the *M. tuberculosis* H37Rv strain at concentrations close to that of isoniazid, one of the main drugs for tuberculosis treatment. In addition, these molecular hits demonstrated selectivity in inhibiting the bacillus when compared to mammalian cell lines. It is important to mention that chemical and metabolic stabilities may be the attrition rate for potential future pharmaceutical development. Although chemical stability can be improved by pharmacotechnical interventions and compounds that undergo high rates of hepatic metabolism are clinically useful, improving these parameters in the initial stages of development should be a priority. Therefore, our findings indicate that this class of molecules may provide lead candidates for the development of new antituberculosis drugs. Hit to lead optimization is in progress and these data will be communicated to specialized literature in due course.

## 3. Experimental Section

Commercially available reactants and solvents were obtained from commercial suppliers and were used without additional purification. The progress of the reaction was monitored using thin-layer chromatography (TLC, Kenilworth, NJ, USA) with Merck TLC Silica gel 60 F254. The melting points were measured using a Microquímica MQAPF-302 apparatus. ^1^H and ^13^C NMR spectra were acquired on an Avance III HD Bruker spectrometer (Bruker Corporation, Fällanden, Switzerland). Chemical shifts (*δ*) were expressed in parts per million (ppm) relative to CDCl_3_ or DMDO-*d6*, which were used as the solvent, and to TMS, as an internal standard. High-resolution mass spectra (HRMS) for **9n** and **9o** were obtained on an LTQ Orbitrap Discovery mass spectrometer (Thermo Fisher Scientific, Bremer, Germany). This system combines an LTQ XL linear ion-trap mass spectrometer and an Orbitrap mass analyzer. The analyses were performed through the direct infusion of the sample in positive-ion mode using electrospray ionization (ESI). For the elemental composition, the calculations used the specific tool included in the Qual Browser module of Xcalibur (Thermo Fisher Scientific, release 2.0.7) software. Compound purity was measured using an Dionex ultimate 3000 HPLC system (Thermo Fisher Scientific Inc., Waltham, MA, USA) equipped with a dual pump, automatic injector, and UV detector. For data acquisition, processing, and elementary composition, calculations were performed using the Chromeleon 6.80 SR11 software (Build 3160). The HPLC conditions: RP column, 5 μm Nucleodur C-18 (250 × 4.6 mm); flow rate, 1.5 mL/min; UV detection, 260 nm; 100% water (0.1% acetic acid) was maintained from 0 to 7 min, followed by a linear gradient from 100% water (0.1% acetic acid) to 90% acetonitrile/methanol (1:1, *v*/*v*) from 7 to 15 min (15–30 min) and subsequently returned to 100% water (0.1% acetic acid) in 5 min (30–35 min) and maintained for more 10 min (35–45 min). All the evaluated compounds were ≥90% pure.

General procedure for the synthesis of 4-(benzyloxy)benzonitriles (**3a**–**3f**).

To a round-bottom flask was added 4-cyanophenol (476 g, 4 mmol) in the presence of potassium carbonate (2.211 g, 16 mmol) using acetone (60 mL) as solvent. Then, benzyl bromide (0.680 g, 4 mmol) was added to the mixture. The reaction system was refluxed (56 °C) for 4 h. After returning to room temperature, the solvent was evaporated under reduced pressure. To the formed residue, 100 mL of distilled water was added and the solid obtained was filtered and washed with water (3 × 30 mL) [19].

General procedure for the synthesis of 4-(benzyloxy)benzylamines (**4a**–**f**).

The reactions were carried out in a two-necked round-bottom flask under argon atmosphere. To a solution containing lithium aluminum hydride (0.398 g, 10.5 mmol) in THF (5 mL) at 0 °C was added dropwise a solution of 4-(benzyloxy)benzonitrile (0.732 g, 3.5 mmol) dissolved in THF (5 mL). After the addition, the temperature was gradually raised to 24 °C and the reaction mixture was stirred for 16 h. At the end of 16 h, the temperature was reduced again to 0 °C and distilled water (2 mL) was slowly added. Then, a 15% NaOH solution (*w*/*v*; 1 mL) and a new portion of distilled water (2 mL) were added. The mixture formed was then stirred for 12 h and then filtered through Celite. The solvent was evaporated under reduced pressure and the product was purified on silica gel column chromatography (chloroform:methanol 95:5 to 70:30) [19].

General procedure for the synthesis of 2-alkyl-4-hydroxyquinolines (**7a**–**e**).

The synthesis of 2-alkyl-4-hydroxyquinolines was carried out using the reaction between substituted anilines (25 mmol) and *β*-ketoesters (29.4 mmol) in the presence of magnesium sulfate (3.611 g, 30 mmol) and acetic acid (0.429 mL, 7.5 mmol) using ethanol (30 mL) as solvent. The mixture was heated under stirring at 80 °C for 16 h. Afterwards, the magnesium sulfate was filtered off and the ethanol was evaporated under reduced pressure yielding the corresponding intermediate *β*-acrilate. Thermal cyclization of the intermediary was carried out by heating in Dowtherm^®^ A (30 mL) at a temperature of 230–250 °C for 15 min. Then, the residue formed was washed with hexane (100 mL). Finally, the solid formed was washed with chloroform (100 mL) and then dried under reduced pressure [10].

General procedure for the synthesis of 2-alkyl-4-chloroquinolines (**8a**–**e**).

To a flask under argon atmosphere containing the 2-alkyl-4-hydroxyquinoline (1 mmol) dissolved in dry toluene (10 mL) was added phosphorus (V) oxychloride (POCl_3_) (0.233 mL, 2.5 mmol) also dissolved in dry toluene. The reaction was kept at 110 °C under stirring for 2 h. After cooling, excess POCl_3_ and solvent were removed under reduced pressure. Finally, the reaction mixture was neutralized with saturated sodium bicarbonate (60 mL). The product was extracted with ethyl acetate (3 × 30 mL), the organic phase was dried over magnesium sulfate, filtered, and concentrated under reduced pressure. The solvent was evaporated on reduced pressure and the product was purified on silica gel column chromatography (hexane:ethyl acetate 90:10) [14].

General procedure for the synthesis of *N*-(4-benzyloxy)-4-aminoquinolines (**9a**–**aa**).

In a Schlenk tube were added 2-alkyl-4-chloroquinoline (1.0 mmol) *N*,*N*-diisopropylethylamine (DIPEA) (0.400 g, 2.3 mmol), dimethylsulfoxide (4 mL), and 4-(benzyloxy)benzylamine of interest (1.4 mmol). The reaction was heated at a temperature of 150 °C for 20 h. After cooling, the product was extracted with ethyl acetate (3 × 30 mL). The organic phase was washed with water (3 × 50 mL), dried over magnesium sulfate and the solvent evaporated under reduced pressure. The product was purified on silica gel column chromatography (hexane:ethyl acetate—70:30 to 0:100). For some substituents, the polarity of the eluent used was changed (ethyl acetate:methanol—100:0 to 70:30) [20].

*N-(4-(benzyloxy)benzyl)-2-methylquinolin-4-amine* (**9a**): Flash column chromatography on silica gel (hexanes/ethyl acetate, 90/10 to 30/70), pale yellow solid, 32% yield, m.p. = 134–136 °C, HPLC: 94%; ^1^H NMR (400 MHz, DMSO-*d*_6_) *δ* ppm: 1.91 (s, 3H), 2.39 (s, 3H), 4.48 (d, *J* = 5.6 Hz, 2H), 5.07 (s, 2H), 6.32 (s, 1H), 6.95–7.01 (m, 2H), 7.31–7.45 (m, 8H), 7.55–7.62 (m, 1H), 7.71 (dd, *J* = 8.4, 1.3 Hz, 1H), 7.84 (t, *J* = 6.1 Hz, 1H), 8.24 (dd, *J* = 8.5, 1.4 Hz, 1H). ^13^C NMR (101 MHz, DMSO) *δ* ppm: 21.66, 45.46, 69.65, 99.19, 115.21 (2C), 117.94, 121.97, 123.82, 128.11 (2C), 128.18, 128.25, 128.68 (2C), 128.87 (2C), 129.45, 131.39, 137.61, 147.82, 150.69, 157.82, 158.62. 

*N-(4-((4-chlorobenzyl)oxy)benzyl)-2-methylquinolin-4-amine* (**9b**): Flash column chromatography on silica gel (hexanes/ethyl acetate, 90/10 to 30/70), pale yellow solid, 29% yield, m.p. = 111–113 °C, HPLC: 91%; ^1^H NMR (400 MHz, DMSO-*d*_6_) *δ* ppm: 2.38 (s, 3H), 4.47 (d, *J* = 5.9 Hz, 2H), 5.06 (s, 2H), 6.28 (s, 1H), 6.94–7.01 (m, 2H), 7.31–7.39 (m, 3H), 7.42–7.48 (m, 4H), 7.57 (t, *J* = 7.6 Hz, 1H), 7.67–7.74 (m, 2H), 8.22 (d, *J* = 8.3 Hz, 1H); ^13^C NMR (101 MHz, DMSO) *δ* ppm: 25.70, 45.43, 68.80, 99.20, 115.22 (2C), 118.06, 121.87, 123.62, 128.67 (2C),128.78, 128.87 (2C), 129.15, 129.90 (2C), 131.69, 132.82,136.68, 148.50, 150.34, 157.59, 158.91.

*N-(4-((4-fluorobenzyl)oxy)benzyl)-2-methylquinolin-4-amine* (**9c**): Flash column chromatography on silica gel (hexanes/ethyl acetate, 90/10 to 50/50), pale yellow solid, 31% yield, m.p. = 134–136 °C, HPLC: 98%; ^1^H NMR (400 MHz, DMSO-*d*_6_) *δ* ppm: 2.37 (s, 3H), 4.47 (d, *J* = 5.9 Hz, 2H), 5.04 (s, 2H), 6.28 (s, 1H), 6.92–7.02 (m, 2H), 7.15–7.26 (m, 2H), 7.30–7.34 (m, 2H), 7.34–7.41 (m, 1H), 7.43–7.52 (m, 2H), 7.52–7.61 (m, 1H), 7.66–7.75 (m, 2H), 8.21 (dd, *J* = 8.3, 1.4 Hz, 1H); ^13^C NMR (101 MHz, DMSO-*d*_6_) *δ* ppm: 25.13, 44.86, 68.36, 98.63, 114.64, 115.11 (d, *J* = 21.3 Hz), 117.49, 121.29, 123.04, 128.08, 128.20, 128.57, 129.78 (d, *J* = 8.3 Hz), 131.05, 133.27 (d, *J* = 3.0 Hz), 147.92, 149.76, 157.11, 158.33, 161.63 (d, *J =* 243.5 Hz).

*N-(4-((3,4-difluorobenzyl)oxy)benzyl)-2-methylquinolin-4-amine* (**9d**): Flash column chromatography on silica gel (hexanes/ethyl acetate, 90/10 to 50/50), pale yellow solid, 30% yield, m.p. = 199–201 °C, HPLC: 99%; ^1^H NMR (400 MHz, DMSO-*d*_6_) *δ* ppm: 2.61 (s, 3H), 4.69 (d, *J* = 5.5 Hz, 2H), 5.08 (s, 2H), 6.69 (s, 1H), 7.00 (d, *J* = 8.1 Hz, 2H), 7.24–7.30 (m, 1H), 7.38–7.47 (m, 4H), 7.59–7.66 (m, 1H), 7.88 (t, *J* = 7.2 Hz, 1H), 8.02 (d, *J* = 8.1 Hz, 1H), 8.66 (d, *J* = 8.3 Hz, 1H), 9.64–9.85 (m, 1H); ^13^C NMR (101 MHz, DMSO-*d*_6_) *δ* ppm: 20.40, 45.95, 68.70, 99.04, 115.67 (2C), 116.47, 117.00 (d, *J* = 17.5 Hz), 117.91 (d, *J* = 17.2 Hz), 120.29, 123.71, 124.71, 124.75, 124.78, 124.81, 126.42, 129.26 (2C), 129.95, 133.42, 135.48 (dd, *J* = 5.8, 3.7 Hz), 138.77, 148.49 (dd, *J* = 41.7, 12.5 Hz), 150.93 (dd, *J* = 41.8, 12.5 Hz), 154.49, 155.48, 158.04.

*N-(4-((4-isopropylbenzyl)oxy)benzyl)-2-methylquinolin-4-amine* (**9e**): Flash column chromatography on silica gel (hexanes/ethyl acetate, 90/10 to 50/50), pale yellow solid, 29% yield, m.p. = 95–97 °C, HPLC: 94%; ^1^H NMR (400 MHz, DMSO-*d*_6_) *δ* ppm: 1.18 (s, 3H), 1.20 (s, 3H), 2.38 (s, 3H), 2.88 (p, *J* = 6.9 Hz, 1H), 4.47 (d, *J* = 5.9 Hz, 2H), 5.01 (s, 2H), 6.28 (s, 1H), 6.94–7.00 (m, 2H), 7.22–7.26 (m, 2H), 7.30–7.39 (m, 5H), 7.53–7.60 (m, 1H), 7.66–7.73 (m, 2H), 8.22 (d, *J* = 7.9 Hz, 1H); ^13^C NMR (101 MHz, DMSO-*d*_6_) *δ* ppm: 24.33, 25.72, 33.65, 45.45, 69.54, 99.19, 115.15 (2C), 118.07, 121.87, 123.60, 126.77 (2C), 128.30 (2C), 128.64 (2C), 128.80, 129.13, 131.45, 134.98, 148.49, 148.53, 150.33, 157.85, 158.92.

*N-(4-((3,5-dimethoxybenzyl)oxy)benzyl)-2-methylquinolin-4-amine* (**9f**): Flash column chromatography on silica gel (hexanes/ethyl acetate, 90/10 to 50/50), pale yellow solid, 26% yield, m.p. = 134–136 °C, HPLC: 92%; ^1^H NMR (400 MHz, DMSO-*d*_6_) *δ* ppm: 2.40 (s, 3H), 3.75 (s, 6H), 4.48 (d, *J* = 5.8 Hz, 2H), 5.03 (s, 2H), 6.32 (s, 1H), 6.44 (t, *J* = 2.3 Hz, 1H), 6.63–6.57 (m, 2H), 6.92–7.02 (m, 2H), 7.40–7.29 (m, 3H), 7.45 (t, *J* = 6.0 Hz, 1H), 7.56 (ddd, *J* = 8.3, 6.8, 1.4 Hz, 1H), 7.71 (ddd, *J* = 8.4, 1.3, 0.5 Hz, 1H), 8.24–8.17 (dd, *J =* 8.5, *J =* 0.8 Hz 1H); ^13^C NMR (101 MHz, DMSO) *δ* ppm: 25.64, 45.79, 55.72 (2C), 69.89, 99.27, 100.16, 106.01 (2C), 115.47 (2C), 118.19, 121.82, 123.54, 128.73 (2C), 128.80, 129.02, 131.71, 140.15, 148.59, 150.48, 157.93, 158.89, 161.17 (2C).

*N-(4-(benzyloxy)benzyl)-6-chloro-2-methylquinolin-4-amine* (**9g**): Flash column chromatography on silica gel (hexanes/ethyl acetate, 90/10 to 30/70), pale yellow solid, 23% yield, m.p. = 165–167 °C, HPLC: 96%; ^1^H NMR (400 MHz, DMSO-*d*_6_) *δ* ppm: 2.39 (s, 3H), 4.45 (d, *J* = 5.8 Hz, 2H), 5.07 (s, 2H), 6.35 (s, 1H) 6.93–7.02 (m, 2H), 7.29–7.46 (m, 7H), 7.57 (dd, *J* = 8.9, 2.3 Hz, 1H), 7.70 (d, *J* = 8.9 Hz, 1H), 7.80 (t, *J* = 5.9 Hz, 1H), 8.39 (d, *J* = 2.3 Hz, 1H); ^13^C NMR (101 MHz, DMSO) *δ* ppm: 24.99, 44.97, 69.09, 99.29, 114.65 (2C), 118.31 120.78, 127.53 (2C), 127.64, 127.68, 128.30 (4C), 129.03, 130.15, 130.60, 137.04, 146.34, 149.27, 157.30, 158.97.

*N-(4-((4-chlorobenzyl)oxy)benzyl) 6-chloro-2-methylquinolin-4-amine* (**9h**): Flash colu-mn chromatography on silica gel (hexanes/ethyl acetate, 90/10 to 50/50), pale yellow solid, 31% yield, m.p. = 177–179 °C, HPLC: 91%; ^1^H NMR (400 MHz, DMSO-*d*_6_) *δ* ppm: 2.39 (s, 2H), 4.45 (d, *J* = 5.8 Hz, 2H), 5.08 (s, 2H), 6.35 (s, 1H), 7.02–6.92 (m, 2H), 7.29–7.36 (m, 2H), 7.42–7.48 (m, 4H), 7.56 (dd, *J* = 8.9, 2.3 Hz, 1H), 7.70 (d, *J* = 9.0 Hz, 2H), 8.37 (d, *J* = 2.4 Hz, 1H); ^13^C NMR (101 MHz, DMSO) *δ* ppm: 25.60, 45.52, 68.82, 99.86, 115.25 (2C), 118.88, 121.32, 128.09, 128.18, 128.87 (4C), 129.55, 129.88 (2C), 130.79, 131.35, 132.82, 136.68, 146.99, 149.79, 157.66, 159.57.

*N-(4-((4-fluorobenzyl)oxy)benzyl) 6-chloro-2-methylquinolin-4-amine* (**9i**): Flash colu-mn chromatography on silica gel (hexanes/ethyl acetate, 90/10 to 50/50), pale solid, 39% yield, m.p. = 170–172 °C, HPLC: 99%; ^1^H NMR (400 MHz, DMSO-*d*_6_) *δ* ppm: 2.40 (s, 3H), 4.46 (d, *J* = 5.9 Hz, 2H), 5.07 (s, 2H), 6.36 (s, 1H), 7.03–6.95 (m, 2H), 7.23–7.13 (m, 2H), 7.38–7.29 (m, 2H), 7.52–7.43 (m, 2H), 7.59–7.52 (m, 2H), 7.71 (d, *J* = 8.9 Hz, 1H), 8.36 (d, *J* = 2.3 Hz, 1H); ^13^C NMR (101 MHz, DMSO) *δ* ppm: 25.63, 45.81, 69.28, 99.93, 115.48 (3C), 115.70, 119.04, 121.27, 128.21, 128.90 (2C), 129.39, 130.14 (d, *J* = 8.3 Hz, 2C), 130.93, 131.48, 133.95 (d, *J* = 3.0 Hz, 1H), 147.25, 149.85, 157.93, 159.64, 162.26 (d, *J* = 243.8 Hz, 1H).

*N-(4-((3,4-difluorobenzyl)oxy)benzyl)-6-chloro-2-methylquinolin-4-amine* (**9j**) Flash column chromatography on silica gel (hexanes/ethyl acetate, 90/10 to 50/50), pale yellow solid, yield 46%, m.p. = 137–139 °C, HPLC: 96%; ^1^H NMR (400 MHz, DMSO-*d*_6_) *δ* ppm: 2.38 (s, 3H), 4.45 (d, *J* = 5.8 Hz, 2H), 5.06 (s, 2H), 6.34 (s, 1H), 6.97–7.00 (m, 2H), 7.31–7.35 (m, 2H), 7.39–7.58 (m, 4H), 7.70 (d, *J* = 8.9 Hz, 1H), 7.79 (t, *J* = 5.9 Hz, 1H), 8.38 (d, *J* = 2.3 Hz, 1H); ^13^C NMR (101 MHz, DMSO-*d_6_*) *δ* ppm: 25.61, 45.49, 68.33, 99.86, 115.23 (2C), 117.07, 117.15 (d, *J* = 17.3 Hz),117.24, 117.87, 117.96 (d, *J* = 17.2 Hz), 118.04, 118.89, 121.31, 124.88, 124.91, 124.93 (dd, *J* = 6.7, 3.5 Hz), 124.94, 124.98, 128.17, 128.87 (2C), 129.54, 130.83, 131.45, 135.43 (dd, *J* = 5.7, 3.6 Hz), 147.03, 148.39 (dd, *J* = 39.6, 12.5 Hz), 149.75, 150.83 (dd, *J* = 39.7, 12.5 Hz), 157.53, 159.59.

*N-(4-((4-isopropylbenzyl) oxy)benzyl)-6-chloro-2-methylquinolin-4-amine* (**9k**): Flash column chromatography on silica gel (hexanes/ethyl acetate, 90/10 to 50/50), pale yellow solid, yield 33%, m.p. = 126–128 °C, HPLC: 91%; ^1^H NMR (400 MHz, DMSO-*d*_6_) *δ* ppm: 1.19 (d, *J* = 6.9 Hz, 8H), 2.38 (s, 2H), 2.88 (p, *J =* 6.8 Hz, 1H), 4.45 (d, *J =* 5.8 Hz, 2H), 5.02 (s, 2H), 6.35 (s, 1H), 6.95–6.98 (m, 2H), 7.23–7.26 (m, 2H), 7.31–7.36 (m, 4H), 7.57 (dd, *J =* 8.9, 2.3 Hz, 1H), 7.70 (d, *J =* 8.9 Hz, 1H), 7.79 (t, *J =* 5.8 Hz, 1H), 8.38 (d, *J =* 2.3 Hz, 1H); ^13^C NMR (101 MHz, DMSO-*d_6_*) *δ* ppm: 23.76, 25.00, 33.08, 44.97, 68.98, 99.29, 114.61 (2C), 118.31, 120.78, 126.20 (2C), 127.72 (2C), 128.29 (2C), 128.57, 129.02, 130.16, 130.51, 134.40, 146.35, 149.27, 157.36, 158.97.

*N-(4-((3,5-dimethoxybenzyl)oxy)benzyl)-6-chloro-2-methylquinolin-4-amine* (**9l**): Flash column chromatography on silica gel (hexanes/ethyl acetate, 90/10 to 50/50), pale yellow solid, 33% yield, m.p. = 147–149 °C, HPLC: 96%; ^1^H NMR (400 MHz, DMSO-*d*_6_) *δ* ppm: 3.74 (d, *J* = 3.3 Hz, 6H), 4.44 (d, *J* = 5.7 Hz, 2H), 5.01 (s, 2H), 6.34 (s, 1H), 6.44 (t, *J* = 2.3 Hz, 1H), 6.59 (d, *J* = 2.3 Hz, 3H), 6.91–7.01 (m, 3H), 7.29–7.35 (m, 2H), 7.56 (dd, *J* = 8.9, 2.2 Hz, 1H), 7.70 (d, *J* = 8.9 Hz, 1H), 7.76 (t, *J* = 5.9 Hz, 1H), 8.38 (d, *J* = 2.3 Hz, 1H); ^13^C NMR (101 MHz, DMSO) *δ* ppm: 25.66, 45.53, 55.63 (2C), 69.53, 99.83, 99.86, 105.82 (2C), 115.23 (2C), 118.91, 121.32, 128.14, 128.84 (2C), 129.13, 130.90, 131.22, 140.01, 147.11, 149.73, 157.77, 159.63, 161.00 (2C).

*N-(4-(benzyloxy)benzyl)-6-bromo-2-methylquinolin-4-amine* (**9m**): Flash column chromatography on silica gel (hexanes/ethyl acetate, 90/10 to 30/70), pale yellow solid, 48% yield, m.p. = 164–166 °C, HPLC: 98%; ^1^H NMR (400 MHz, DMSO-*d*_6_) *δ* ppm: 2.38 (s, 3H), 4.45 (d, *J =* 5.8 Hz, 2H), 5.07 (s, 2H), 6.34 (s, 1H), 6.93–7.02 (m, 2H), 7.31–7.45 (m, 7H), 7.79 (t, *J* = 5.9 Hz, 1H), 8.53 (d, *J* = 2.1 Hz, 1H); ^13^C NMR (101 MHz, DMSO) *δ* ppm: 25.71, 45.54, 69.66, 99.87, 115.21 (2C), 116.44, 119.53, 124.48, 128.10 (2C), 128.24, 128,87 (4C), 131.07, 131.19, 132.09, 137.61, 147.32, 149.64, 157.87, 159.75.

*N-(4-((4-chlorobenzyl)oxy)benzyl)-6-bromo-2-methylquinolin-4-amine* (**9n**): Flash colu-mn chromatography on silica gel (hexanes/ethyl acetate, 90/10 to 50/50), pale yellow solid, 44% yield, m.p. = 184–186 °C, HPLC: 92%; ^1^H NMR (400 MHz, DMSO-*d*_6_) *δ* ppm: 2.39 (s, 3H), 4.43 (d, *J* = 5.7 Hz, 2H), 5.07 (s, 2H), 6.34 (s, 1H), 6.93–7.02 (m, 2H), 7.30–7.36 (m, 2H), 7.41–7.47 (m, 4H), 7.58 (t, *J* = 5.9 Hz, 1H), 7.61 –7.70 (m, 2H), δ 8.47 (d, *J* = 2.0 Hz, 1H); ^13^C NMR (101 MHz, DMSO) *δ* ppm: 25.64, 45.80, 69.17, 99.93, 115.49 (2C), 116.40, 119.63, 124.44 128.83 (2C), 128.93 (2C), 129.75 (2C), 131.07, 131.54, 132.01, 132.88, 136.80, 147.41, 149.77, 157.85, 159.73, HRMS (ESI): m/z calc. for C_24_H_21_BrClN_2_O [M + H]^+^: 467.0520; obt.: 467.0509.

*N-(4-((4-fluorobenzyl)oxy)benzyl) 6-bromo-2-methylquinolin-4-amine* (**9o**): Flash column chromatography on silica gel (hexanes/ethyl acetate, 90/10 to 50/50), pale solid, 35% yield, m.p. = 173–175 °C, HPLC: 98%; ^1^H NMR (400 MHz, DMSO-*d*_6_) *δ* ppm: 2.40 (s, 3H), 4.46 (s, 2H), 5.07 (d, *J* = 0.9 Hz, 2H), 6.36 (s, 1H), 6.95–7.03 (m, 2H), 7.13–7.23 (m, 2H), 7.29–7.37 (m, 2H), 7.43–7.53 (m, 2H), 7.58 (t, *J* = 5.9 Hz, 1H), 7.65 (dd, *J* = 3.0, 1.3 Hz, 2H), 8.50 (dd, *J* = 1.9, 0.7 Hz, 1H); ^13^C NMR (101 MHz, DMSO-*d*_6_) *δ* ppm: 25.66, 45.81, 69.28, 99.94, 115.48 (3C), 115.70, 116.39, 119.64, 124.44, 128.91 (2C), 130.14 (d, *J* = 8.2 Hz,2C), 131.10, 131.47, 132.00, 133.95 (d, *J* = 3.0 Hz), 147.44, 149.75, 157.93, 159.75, 162.26 (d, *J* = 243.8 Hz), HRMS (ESI): m/z calc. for C_24_H_21_BrFN_2_O [M + H]^+^: 451.0816; obt.: 451.0790.

*N-(4-((3,4-difluorobenzyl)oxy)benzyl) 6-bromo-2-methylquinolin-4-amine* (**9p**): Flash column chromatography on silica gel (hexanes/ethyl acetate, 90/10 to 50/50), pale yellow solid, 46% yield, m.p. = 142–144 °C, HPLC: 97%; ^1^H NMR (400 MHz, DMSO-*d*_6_) *δ* ppm: 2.37 (s, 3H), 4.45 (d, *J* = 5.7 Hz, 2H), 5.06 (s, 2H), 6.34 (s, 1H), 6.92–7.04 (m, 2H), 7.23–7.37 (m, 3H), 7.37–7.55 (m, 2H), 7.59–7.73 (m, 2H), 7.81 (t, *J* = 5.9 Hz, 1H), 8.52 (d, *J* = 2.1 Hz, 1H); ^13^C NMR (101 MHz, DMSO) *δ* ppm: 25.63, 45.48, 68.33, 99.86, 115.23 (2C), 116.46, 117.96 (d, *J* = 17.1 Hz), 117.16 (d, *J* = 17.4 Hz), 119.48, 124.47, 124.98, 128.88 (2C), 130.98, 131.44, 132.14, 135.43 (dd, *J* = 5.7, 3.8 Hz), 148.39 (dd, *J* = 39.4, 12.5 Hz), 149.66, 151.03 (d, *J* = 12.6 Hz), 157.53, 159.69.

*N-(4-((4-isopropylbenzyl)oxy)benzyl) 6-bromo-2-methylquinolin-4-amine* (**9q**): Flash column chromatography on silica gel (hexanes/ethyl acetate, 90/10 to 50/50), pale yellow solid, m.p. = 135–137 °C, 27% yield, HPLC: 97%; ^1^H NMR (400 MHz, DMSO-*d*_6_) *δ* ppm: 1.18 (s, 3H), 1.20 (s, 3H), 2.37 (s, 3H), 2.87 (hept, *J* = 6.9 Hz, 1H), 4.44 (d, *J* = 5.8 Hz, 2H), 5.01 (s, 2H), 6.34 (s, 1H), 6.94–6.99 (m, 2H), 7.22–7.26 (m, 2H), 7.30–7.37 (m, 4H), 7.61–7.70 (m, 2H), 7.79 (t, *J* = 5.9 Hz, 1H), 8.53 (d, *J* = 2.1 Hz, 1H); ^13^C NMR (101 MHz, DMSO) *δ* ppm: 24.33 (2C), 25.69, 45.52, 69.52, 99.86, 115.15 (2C), 116.43,119.51, 124.48, 126.77 (2C), 128.29 (2C), 128.85 (2C), 129.13 (2C), 131.06, 131.09, 132.10, 134.96, 147.29, 148.49, 149.64, 157.91, 159.73.

*N-(4-((3,5-dimethoxybenzyl)oxy)benzyl)-6-bromo-2-methylquinolin-4-amine* (**9r**): Flash column chromatography on silica gel (hexanes/ethyl acetate, 90/10 to 50/50), pale yellow solid, m.p. = 151–153 °C, 36% yield, HPLC: 94%; ^1^H NMR (400 MHz, DMSO-*d*_6_) *δ* ppm: 2.38 (s, 3H), 3.73 (s, 6H), 4.45 (d, *J* = 5.7 Hz, 2H), 5.01 (s, 2H), 6.35 (s, 1H), 6.44 (t, *J* = 2.3 Hz, 1H), 6.58 (d, *J* = 2.3 Hz, 2H), 6.92–7.01 (m, 2H), 7.28–7.36 (m, 2H), 7.60–7.66 (m, 1H), 7.66–7.72 (m, 1H), 7.86 (t, *J* = 5.9 Hz, 1H), 8.53 (d, *J* = 2.2 Hz, 1H); ^13^C NMR (101 MHz, DMSO) *δ* ppm: 25.46, 45.55, 55.64 (2C), 69.53, 99.83, 99.85, 105.82 (2C), 115.25 (2C), 116.54, 119.43, 124.53, 128.88 (2C), 130.69, 131.13, 132.27, 140.01, 146.88, 149.84, 157.79, 159.54, 161.00 (2C).

*N-(4-(benzyloxy)benzyl)-6-bromo-2-ethylquinolin-4-amine* (**9s**): Flash column chromatography on silica gel (hexanes/ethyl acetate, 90/10 to 50/50), pale yellow solid, 44% yield, m.p. = 136–138 °C, HPLC: 93%; ^1^H NMR (400 MHz, DMSO-*d*_6_) *δ* ppm: 1.17 (t, *J* = 7.6 Hz, 3H), 2.64 (q, *J* = 7.6 Hz, 2H), 4.45 (d, *J* = 5.7 Hz, 2H), 5.07 (s, 2H), 6.35 (s, 1H), 6.94–7.02 (m, 2H), 7.31–7.45 (m, 7H), 7.63–7.69 (m, 2H), 7.79 (t, *J* = 5.9 Hz, 1H), 8.52 (d, *J* = 1.9 Hz, 1H); ^13^C NMR (101 MHz, DMSO-*d*_6_) *δ* ppm: 14.05, 32.14, 45.58, 69.63, 98.98, 115.19 (2C), 116.45, 119.75, 124.47, 128.08 (2C), 128.23, 128.86 (2C), 128.96 (2C), 131.23, 131.26, 132.04, 137.61, 147.29, 149.73, 157.83, 164.61.

*N-(4-((4-chlorobenzyl)oxy)benzyl)-6-bromo-2-ethylquinolin-4-amine* (**9t**) Flash column chromatography on silica gel (hexanes/ethyl acetate, 90/10 to 50/50), pale yellow solid, 30% yield, m.p. = 122–124 °C, HPLC: 92%; ^1^H NMR (400 MHz, DMSO-*d*_6_) *δ* ppm: 1.16 (t, *J* = 7.6 Hz, 3H), 2.64 (q, *J* = 7.6 Hz, 2H), 4.45 (d, *J* = 5.6 Hz, 2H), 5.07 (s, 2H), 6.34 (s, 1H), 6.93–7.01 (m, 2H), 7.30–7.36 (m, 2H), 7.40–7.49 (m, 4H), 7.61–7.71 (m, 2H), 7.79 (t, *J* = 5.9 Hz, 1H), 8.51 (d, *J* = 2.0 Hz, 1H); ^13^C NMR (101 MHz, DMSO) *δ* ppm: 14.02, 32.13, 45.57, 68.79, 98.99, 115.24, 116.45, 119.74, 124.45 (2C), 128.86 (2C), 128.97 (2C), 129.86 (2C), 131.25, 131.40, 132.04, 132.80, 136.68, 147.28, 149.72, 157.62, 164.60.

*N-(4-(benzyloxy)benzyl)-6-methoxy-2-methylquinolin-4-amine* (**9u**): Flash column chromatography on silica gel (hexanes/ethyl acetate, 90/10 to 30/70), pale yellow solid, 32% yield, m.p. = 96–98 °C, HPLC: 98%; ^1^H NMR (400 MHz, DMSO-*d*_6_) *δ* ppm: 2.35 (s, 3H), 3.89 (s, 3H), 4.48 (d, *J* = 5.8 Hz, 2H), 5.07 (s, 2H), 6.26 (s, 1H), 6.94–7.03 (m, 2H), 7.23 (dd, *J* = 9.0, 2.8 Hz, 1H), 7.55 (t, *J* = 5,9 Hz, 1H) 7.31–7.45 (m, 7H), 7.60 (s, 2H), 7.60–7.66 (m, 2H); ^13^C NMR (101 MHz, DMSO) *δ* ppm: 25.42, 45.54, 56.08, 69.66, 99.35, 101.37, 115.21 (2C), 118.41, 120.60, 128.11 (2C), 128.24, 128.68 (2C), 128.87 (2C), 130.22, 131.66, 137.63, 144.02, 149.63, 155.98, 156.35, 157.80.

*N-(4-((4-chlorobenzyl)oxy)benzyl)-6-methoxy-2-methylquinolin-4-amine* (**9v**): Flash column chromatography on silica gel (hexanes/ethyl acetate, 90/10 to 30/70), pale yellow solid, 27% yield, m.p. = 259–261 °C, HPLC: 90%; ^1^H NMR (400 MHz, DMSO-*d*_6_) *δ* ppm: 2.57 (s, 3H), 3.96 (s, 3H), 4.67 (d, *J* = 5.9 Hz, 2H), 5.08 (s, 2H), 6.62 (s, 1H), 6.95–7.03 (m, 2H), 7.36–7.47 (m, 6H), 7.48–7.54 (m, 1H), 7.94 (d, *J* = 9.2 Hz, 1H), 8.11 (d, *J* = 2.7 Hz, 1H), 9.60 (t, *J* = 6.0 Hz, 1H); ^13^C NMR (101 MHz, DMSO) *δ* ppm: 19.77, 45.34, 56.45, 68.66, 98.25, 103.37, 115.13 (2C), 117.14, 121.56, 123.84, 128.33 (2C), 128.68 (2C), 129.25 (2C), 129.53, 132.39, 133.44, 136.20, 151.90, 154.04, 157.44, 157.62.

*N-(4-((4-fluorobenzyl)oxy)benzyl)-6-methoxy-2-methylquinolin-4-amine* (**9x**): Flash column chromatography on silica gel (hexanes/ethyl acetate, 90/10 to 0/100), pale yellow solid, 28% yield, m.p. = 141–143 °C, HPLC: 97%; ^1^H NMR (400 MHz, DMSO-*d*_6_) *δ* ppm 2.36 (s, 3H), 3.89 (s, 3H), 4.49 (d, *J* = 5.8 Hz, 2H), 5.05 (s, 2H), 6.28 (s, 1H), 6.98 (d, *J* = 8.2 Hz, 2H), 7.18–7.26 (m, 3H), 7.33 (d, *J* = 8.1 Hz, 2H), 7.46–7.50 (m, 2H), 7.62–7.66 (m, 2H), 7.70 (t, *J* = 6.0 Hz, 1H); ^13^C NMR (101 MHz, DMSO) *δ* ppm: 25.11, 45.53, 56.12, 68.94, 99.32, 101.48, 115.23, 115.67 (d, *J* = 21,8 Hz, 2C), 118.35, 120.84, 128.70 (2C), 129.72, 130.34 (d, *J* = 9,09 Hz, 2C), 131.63, 133.83 (d, *J* = 3,03 Hz), 143.40, 149.92, 156.09, 157.70, 162.20 (d, *J* = 244.4 Hz).

*N-(4-((3,4-difluorobenzyl) oxy)benzyl)-6-methoxy-2-methylquinolin-4-amine* (**9y**): Flash column chromatography on silica gel (hexanes/ethyl acetate, 90/10 to 0/100), pale yellow solid, m.p. = 104–106 °C, 46% yield, HPLC: 97%; ^1^H NMR (400 MHz, DMSO-*d*_6_) *δ* ppm: 2.34 (s, 3H), 3.88 (s, 3H), 4.48 (d, *J* = 5.8 Hz, 2H), 5.06 (s, 2H), 6.25 (s, 1H), 6.96–7.01 (m, 2H), 7.22 (dd, *J* = 9.1, 2.7 Hz, 1H), 7.30–7.35 (m, 2H), 7.40–7.54 (m, 2H), 7.55–7.60 (m, 1H), 7.59–7.63 (m, 2H); ^13^C NMR (101 MHz, DMSO) *δ* ppm: 25.34, 45.49, 56.08, 68.34, 99.34, 101.35, 115.24 (2C), 117.57 (dd, *J* = 80.1, 17.1 Hz), 118.37, 120.65, 124.95 (dd, *J* = 6.7, 3.5 Hz, 1C), 128.69 (2C),130.11, 131.90, 135.46 (dd, *J* = 5.8, 3.7 Hz), 143.86, 124.95 (dd, *J* = 6.7, 3.5 Hz), 149.66, 150.83 (dd, *J* = 39.6, 12.5 Hz), 155.99, 156.27, 157.46.

*N-(4-((4-isopropylbenzyl)oxy)benzyl)-6-methoxy-2-methylquinolin-4-amine* (**9w**): Flash column chromatography on silica gel (hexanes/ethyl acetate, 90/10 to 0/100), pale yellow solid, m.p. = 133–135 °C, 33% yield, HPLC: 91%; ^1^H NMR (400 MHz, DMSO-*d*_6_) *δ* ppm: 1.18 (d, *J* = 2.3 Hz, 3H), 1.20 (d, *J* = 2.4 Hz, 3H), 2.36 (s, 2H), 2.88 (p, *J* = 6.7 Hz, 1H), 3.89 (s, 3H), 4.48 (d, *J* = 5.8 Hz, 2H), 5.02 (s, 2H), 6.29 (s, 1H), 6.95–6.99 (m, 2H), 7.22–7.27 (m, 3H), 7.31–7.36 (m, 4H), 7.62–7.67 (m, 2H), 7.73 (t, *J* = 5.9 Hz, 1H); ^13^C NMR (101 MHz, DMSO) *δ* ppm: 24.33, 25.02, 33.54, 45.54, 56.13, 69.53, 99.29, 101.50, 115.17 (2C), 118.32, 120.92, 126.77 (2C), 128.30 (2C), 128.70 (2C), 129.53, 129.56, 131.40, 134.98, 143.19, 148.49, 150.01, 156.00, 156.11 157.87.

*N-(4-((3,5-dimethoxybenzyl)oxy)benzyl)-6-methoxy-2-methylquinolin-4-amine* (**9z**): Flash column chromatography on a silica gel (hexanes/ethyl acetate, 90/10 to 50/50), pale yellow solid, 33% yield, m.p. = 164–166 °C, HPLC: 96%; ^1^H NMR (400 MHz, DMSO-*d*_6_) *δ* ppm: 2.35 (s, 3H), 3.73 (s, 6H), 3.89 (s, 3H), 4.47 (d, *J* = 5.8 Hz, 2H), 5.01 (s, 2H), 6.25 (s, 1H), 6.41–6.49 (m, 1H), 6.59 (d, *J* = 2.3 Hz, 2H), 6.91–7.02 (m, 2H), 7.22 (dd, *J* = 9.1, 2.7 Hz, 1H), 7.29–7.36 (m, 2H), 7.55 (t, *J* = 5.7 Hz, 1H), 7.59–7.66 (m, 2H); ^13^C NMR (101 MHz, DMSO) *δ* ppm: 25.41, 45.54, 55.63 (2C), 56.07, 69.53, 99.34, 99.83, 101.35,105.83 (2C), 115.24 (2C), 118.40, 120.59, 128.66 (2C), 130.23, 131.68, 140.03, 144.02, 149.61, 155.96, 156.34,157.71, 161.00 (2C).

*N-(4-(benzyloxy)benzyl)-2-ethyl-6-methoxyquinolin-4-amine* (**9aa**): Flash column chromatography on a silica gel (hexanes/ethyl acetate, 90/10 to 50/50), pale yellow solid, 34% yield, m.p. = 118–120 °C, HPLC: 92%; ^1^H NMR (400 MHz, DMSO-*d*_6_) *δ* ppm: 1.16 (t, *J* = 7.6 Hz, 3H), 2.62 (q, *J* = 7.6 Hz, 2H), 3.89 (s, 3H), 4.48 (d, *J* = 5.8 Hz, 2H), 5.07 (s, 2H), 6.27 (s, 1H), 6.96 –7.00 (m, 2H), 7.23 (dd, *J* = 9.1, 2.7 Hz, 1H), 7.33 –7.44 (m, 7H), 7.56 (t, *J* = 5.9 Hz, 1H), 7.61 (d, *J* = 2.7 Hz, 1H), 7.65 (d, *J* = 9.1 Hz, 1H); ^13^C NMR (101 MHz, DMSO), *δ* ppm: 14.25, 31.89, 42.04, 45.61, 56.08, 69.64, 98.44, 101.35, 115.19 (2C), 118.65, 120.57, 128.08 (2C), 128.23, 128.78 (2C), 128.86 (2C), 130.37, 131.69, 137.63, 143.92, 149.72, 156.00, 157.77, 161.31, 161.36.

### 3.1. Minimum Inhibitory Concentration

The determination of the minimum inhibitory concentrations (MICs) for each synthesized compound was performed in 96-well microplates. Isoniazid was used as positive control and compound solutions were prepared at concentrations of 2 mg/mL in DMSO. They were diluted in Middlebrook 7H9 medium containing 10% ADC (albumin, dextrose, and catalase) to a concentration of 20 μg/mL of each compound containing 2% DMSO (Sigma-Aldrich, St. Louis, MO, USA). After, they were evaluated for the presence of crystals—if crystals were present, the mixture was diluted once more to half of the previous concentration. It is noteworthy that only molecules capable of forming a real solution were evaluated. Serial 2-fold dilutions of each drug in 100 μL of Middlebrook 7H9 medium containing 10% ADC (BD co.) were prepared directly in 96-well plates at concentration ranges starting with the maximum concentration allowed by the solubility of each compound. Growth controls without antibiotic and sterility controls without inoculation were included. The MIC was determined for *M. tuberculosis* H37Rv. Mycobacterial strains were grown in Middlebrook 7H9 containing 10% OADC (oleic acid, albumin, dextrose, and catalase) and 0.05% tween 80, and cells were vortexed with sterile glass beads (4 mm) for 5 min to disrupt clamps and then allowed to settle for 20 min. The supernatants were measured using a spectrophotometer at an absorbance of 600 nm. The Mtb suspensions were aliquoted and stored at −20 °C. Each suspension was appropriately diluted in Middlebrook 7H9 broth containing 10% ADC to achieve an optical density of 0.006 at 600 nm, and 100 μL was added to each well of the plate except to the sterility controls. The plates were covered, sealed, and incubated at 37 °C. After 7 days of incubation, 60 μL of 0.01% resazurin solution was added to each well, and the plate was incubated for an additional 48 h at 37 °C. In the MIC assay, a color change from blue to pink indicates bacterial growth, and the MIC was established as the lowest compound concentration prior to color change. Three tests were performed independently for each chemical structure and the MIC values were reported as the highest value from the three assays.

### 3.2. Cellular Viability Evaluation

Cellular viability was determined using two distinct methods—3-(4,5-dimethylthiazol-2-yl)-2,5-diphenyl-2*H*-tetrazolium bromide (MTT) and neutral red uptake (NRU). The cell lines employed in the evaluation were Vero and HepG2 cells. Both cell lines were grown with Dulbecco’s Modified Eagle Medium (DMEM-Gibco, Grand Island, NY, USA) and supplemented with 10% fetal bovine serum by Invitrogen, 1% antibiotics (penicillin and streptomycin) by Gibco, and 0.1% fungizone by Gibco. For the MTT and NRU assays, Vero (2 × 10^3^ cells/well) and HepG2 (4 × 10^3^ cells/well) cells were seeded in 96-well culture plates and incubated overnight. The *N*-(4-(benzyloxy)benzyl)-4-aminoquinolines were diluted at concentrations of 3 µM using DMSO 1% and were incubated with the cell lines for 72 h at 37 °C. In the MTT investigation, after incubation for 72 h at 37 °C under 5% CO_2_, the cells were incubated with MTT solution (5 mg/mL, Sigma-Aldrich, St. Louis, MO, USA) for 4 h. The formazan crystals were solubilized in 100 µL of DMSO. An EZ Read 400 microplate reader (Biochrom, Cambridge, UK) was used to measure the absorbance at 570 nm. The mean absorbance of negative control wells was established as the maximum viability and the values of treated cells were calculated as the percentage of vehicle control (1% DMSO). The precipitated purple formazan crystals were directly equivalent to the number of live cells with active mitochondrial metabolism. In the NRU investigation, after 72 h of cell incubation, PBS was used to wash the cells and 200 µL of neutral red dye solution (25 µg/mL, Sigma-Aldrich, St. Louis, MO, USA) prepared in serum-free medium was added to the plate and incubated for 3 h at 37 °C under 5% CO_2_. Cells were washed with PBS followed by the addition of 100 µL of desorb solution (ethanol/acetic acid/water, 50:1:49) for 30 min with smooth homogenization to extract neutral red dye from the viable cells. An EZ Read 400 microplate reader was operated to measure the absorbance at 562 nm, and the cell viability was attributed as a percentage, considering the vehicle control cell (1% DMSO) as maximum cell viability.

### 3.3. Chemical Stability

The experiment was carried out by the Center for Applied Mass Spectrometry (CEMSA), São Paulo, Brazil. In brief, the test compounds (10 µM) were incubated at 37 °C for 24 h in the presence of pH-controlled buffer solution at pH 1.2 (simulating the pH of the stomach—0.1 M HCl), pH 7.4 (simulating plasma pH—phosphate buffer) and pH 9.1 (simulating intestinal pH—0.1 M NH_4_HCO_3_). Afterward, compounds were quantified by HPLC–MS/MS. Alprenolol drug was used as an analytical control (data not shown). The results were presented as the percentage, comparing the signal at time zero of the assay (100%) with the signal produced by concentrations after the incubation period.

### 3.4. Permeability

The experiment was carried out by the Center for Applied Mass Spectrometry (CEMSA), São Paulo, Brazil. In brief, **the** Parallel Artificial Membrane Permeability Assay (PAMPA) assay consists of quantifying the test compound (via HPLC–MS/MS) after an incubation period, in two solutions separated by an artificial lipid membrane. The result of this test is expressed in units of diffusion rate (permeation). The procedure comprised the following steps: (1) preparing the membrane, which contains lipids, with specific activating solutions, creating a hydrophobic surface that simulates the intestinal epithelial cell; (2) adding the test compound at a concentration of 10 µM to the donor aqueous phase (buffered pH 7.4); (3) after 5 h at room temperature, an aliquot of the solution receptor (buffered at pH 7.4) is removed, in which the compound is transported by passive diffusion, for quantification by HPLC–MS/MS.

### 3.5. Metabolic Stability

The experiment was carried out by the Center for Applied Mass Spectrometry (CEMSA), São Paulo, Brazil. In brief, the metabolic stability assay was performed in the presence of rat liver microsomes. Compounds were incubated at 37 °C in a buffered solution containing 1 mg/mL of microsomal protein and nicotinamide adenine dinucleotide (NADH). The reaction was stopped at different times using acetonitrile. The compound concentration was determined by HPLC–MS/MS at each incubation time (0, 5, 15, and 30 min) and the percentage remaining versus time curve was determined. Verapamil was used as an analytical control (data not shown).

## Figures and Tables

**Table 1:** Yields after purification of 4-amino-*N*-(4-benzyloxy)quinolines **9a**–**9aa**, ClogP values, and in vitro activity against *M. tuberculosis* H37Rv.

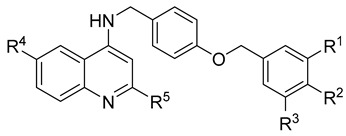								
Entry	R^1^	R^2^	R^3^	R^4^	R^5^	Yield (%) ^a^	ClogP ^b^	MIC (µM)
**9a**	H	H	H	H	Me	32	6.13	>7
**9b**	H	Cl	H	H	Me	29	6.85	>12.9
**9c**	H	F	H	H	Me	31	6.28	26.8
**9d**	F	F	H	H	Me	30	6.35	25.6
**9e**	H	*i*Pr	H	H	Me	29	7.55	27
**9f**	OMe	H	OMe	H	Me	26	6.14	12.1
**9g**	H	H	H	Cl	Me	28	6.94	6.4
**9h**	H	Cl	H	Cl	Me	31	7.66	5.9
**9i**	H	F	H	Cl	Me	29	7.09	6.1
**9j**	F	F	H	Cl	Me	46	7.16	5.9
**9k**	H	*i*Pr	H	Cl	Me	33	8.37	5.8
**9l**	OMe	H	OMe	Cl	Me	33	6.95	5.7
**9m**	H	H	H	Br	Me	48	7.09	5.8
**9n**	H	Cl	H	Br	Me	44	7.81	2.7
**9o**	H	F	H	Br	Me	35	7.24	2.8
**9p**	F	F	H	Br	Me	46	7.31	5.3
**9q**	H	*i*Pr	H	Br	Me	27	8.52	5.3
**9r**	OMe	H	OMe	Br	Me	36	7.10	5.1
**9s**	H	H	H	Br	Et	44	7.62	5.6
**9t**	H	Cl	H	Br	Et	30	8.34	5.2
**9u**	H	H	H	OMe	Me	32	6.45	13
**9v**	H	Cl	H	OMe	Me	27	7.16	11.9
**9x**	H	F	H	OMe	Me	28	6.59	12.4
**9y**	F	F	H	OMe	Me	46	6.67	5.9
**9w**	H	*i*Pr	H	OMe	Me	33	7.88	23.4
**9z**	OMe	H	OMe	OMe	Me	33	6.46	11.2
**9aa**	H	H	H	OMe	Et	34	6.98	12.5
**INH**	-	-	-	-	-	-	-	2.3

^a^ Yield of purified compounds; ^b^ ClogP calculated by ChemBioDraw Ultra, version 12.0.2.1076. INH, isoniazid.

**Table 2:** Viability of Vero and HepG2 cells, chemical stability, permeability, and metabolic stability of 4-amino-*N*-(4-benzyloxy)quinolines **9n** and **9o**.

Entry	Selectivity ^a^		Chemical Stability ^d^			Permeability	Metabolic Stability	
	CC_50_^a^ Vero (µM)	CC_50_^a^ HepG2 (µM)	pH 1.2 ^e^ (%)	pH 7.4 ^f^ (%)	pH 9.1 ^g^ (%)	PAMPA (10^−6^ cm/s)	Cl_int_^h^ (mL/min/kg)	*t*_1/2_ ^i^ (min)
**9n**	>3 ^b^ >3 ^c^	>3 ^b^ >3 ^c^	14.9	5.0	13.8	0.3	39	19
**9o**	>3 ^b^ >3 ^c^	>3 ^b^ >3 ^c^	22.9	7.6	11.1	3.0	56	7.9

^a^ The selectivity of the compounds was studied on Vero and HepG2 cells. The results were expressed as the concentration able to reduce cell viability by 50% (CC_50_) determined by MTT and neutral red assays. ^b^ Determined by the MTT method. ^c^ Determined by the neutral red method. ^d^ Percentage of remaining compound after incubation at 37 °C for 24 h. ^e^ A 0.1 M HCl solution. ^f^ PBS. ^g^ A 0.1 M NH_4_HCO_3_ solution. ^h^ Rat liver microsomes intrinsic clearance. ^i^ Half-life.

## Data Availability

The data presented in this study are available herein and in the Supplementary Materials.

## Supplementary Materials

The following supporting information can be downloaded at: https://www.mdpi.com/article/10.3390/molecules27082556/s1. Figures S1–S56: ^1^H and ^13^C spectra for compounds **9a**–**9aa** and mass spectra for hit compounds **9n** and **9o**.

## Sample Availability

Samples of the synthesized compounds are available from the authors.
