# Supplementary material for: Synthesis and Antimycobacterial Evaluation of N-(4-(Benzyloxy)benzyl)-4-aminoquinolines
Source: Molecules. 2022 Apr 15;27(8):2556. doi: 10.3390/molecules27082556 (PMC9027910; doi:10.3390/molecules27082556)
Supplement: Supplementary file 1 [file molecules-27-02556-s001.zip › molecules-1668803-supplementary.pdf]

Supporting Information

Synthesis and Antimycobacterial Evaluation of *N*-(4-(Benzyloxy)benzyl)-4-aminoquinolines

Estevão Silveira Grams, Alessandro Silva Ramos, Mauro Neves Muniz, Raoní Scheibler Rambo, Marcia Alberton Perelló, Nathalia Sperotto, Laura Calle González, Lovaine Duarte, Adilio Silva Dadda, Guilherme Arraché Gonçalves, Cristiano Valim Bizarro, Luiz Augusto Basso, Pablo Machado*

Table of contents:

1. ^{1}H NMR and ^{13}C NMR spectra of synthesized compounds **9a-aa**.
2. Mass spectra of synthesized compounds **9n** and **9o**.

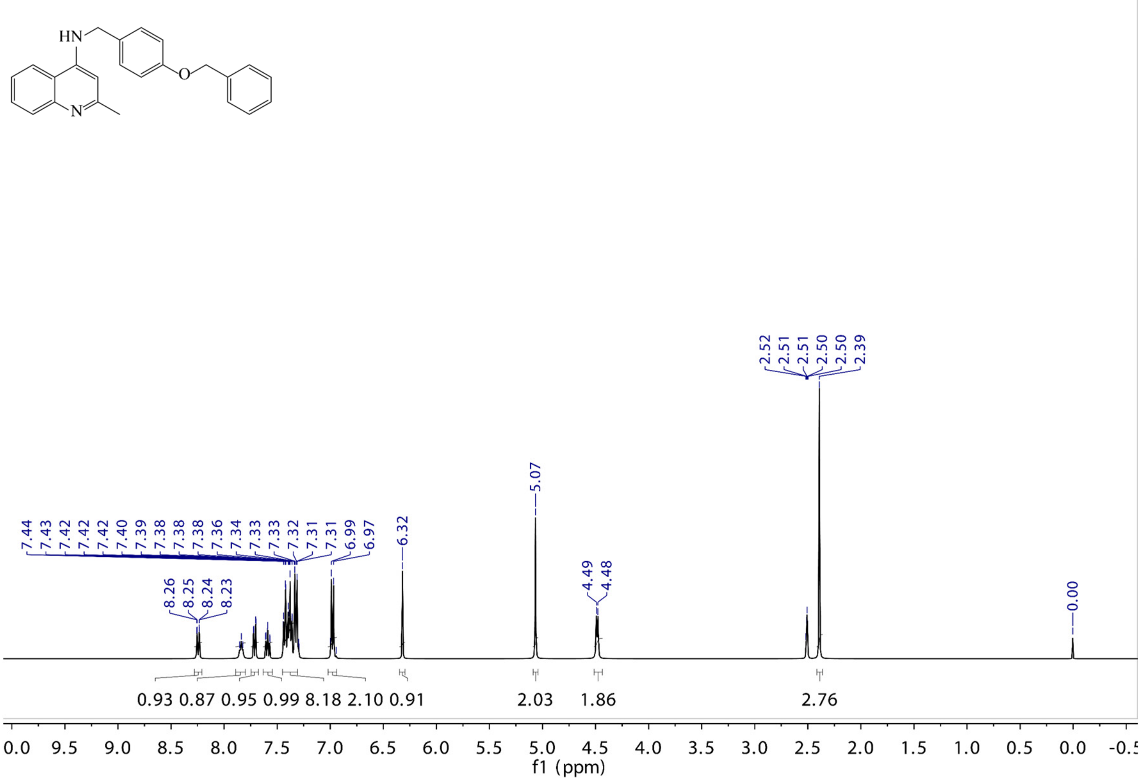

Figure S1: ^{1}H spectrum of **9a** in DMSO-*d*$_6$.

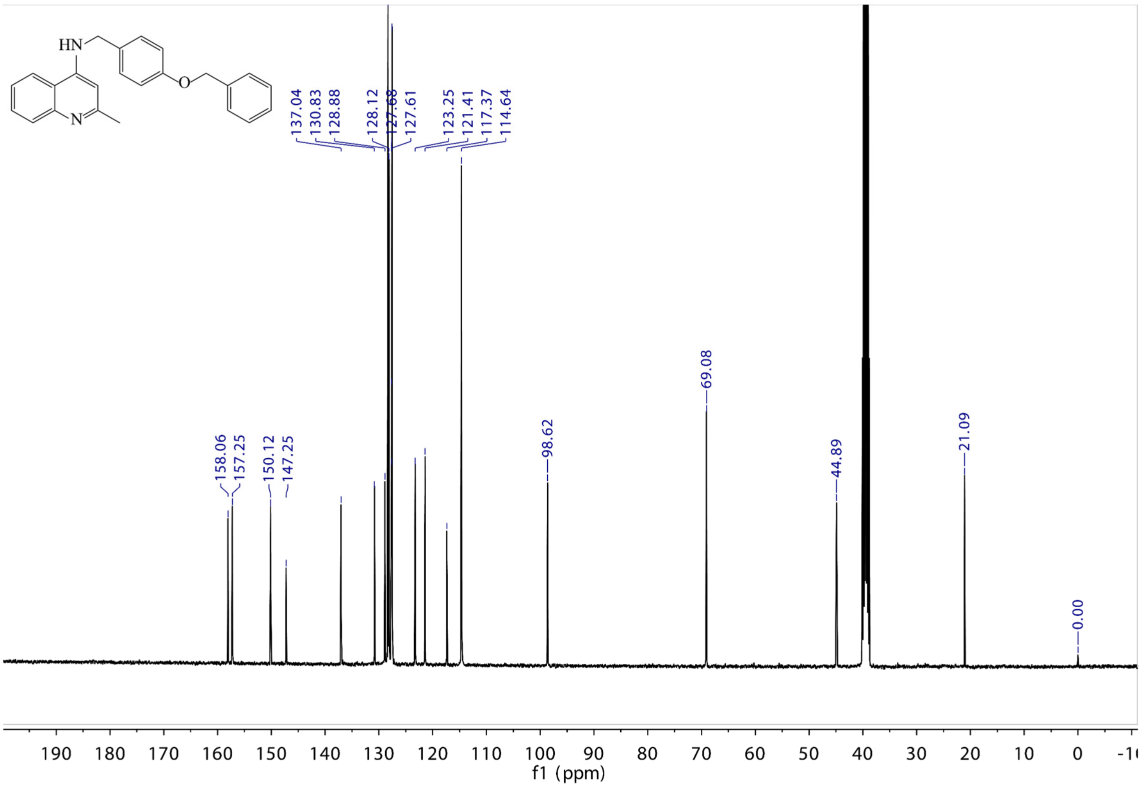

Figure S2: ^{13}C spectrum of **9a** in DMSO-*d*$_6$.

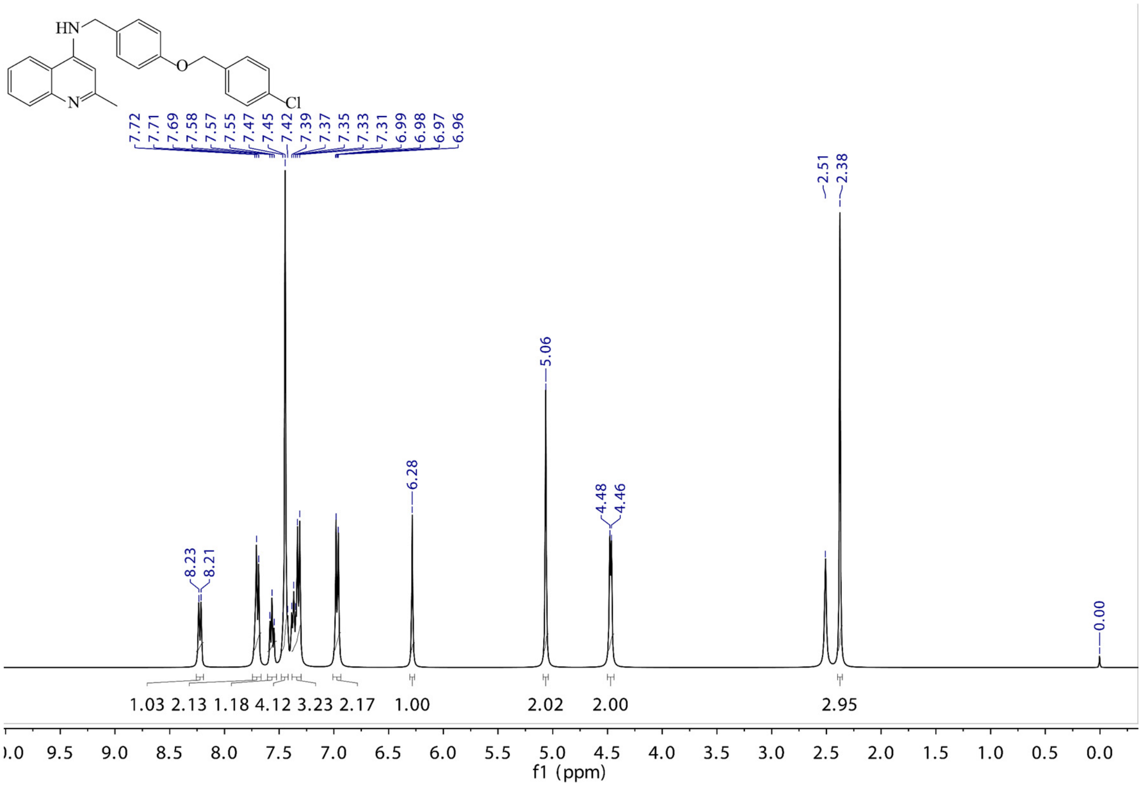

Figure S3: ^{1}H spectrum **9b** of in DMSO-*d₆*.

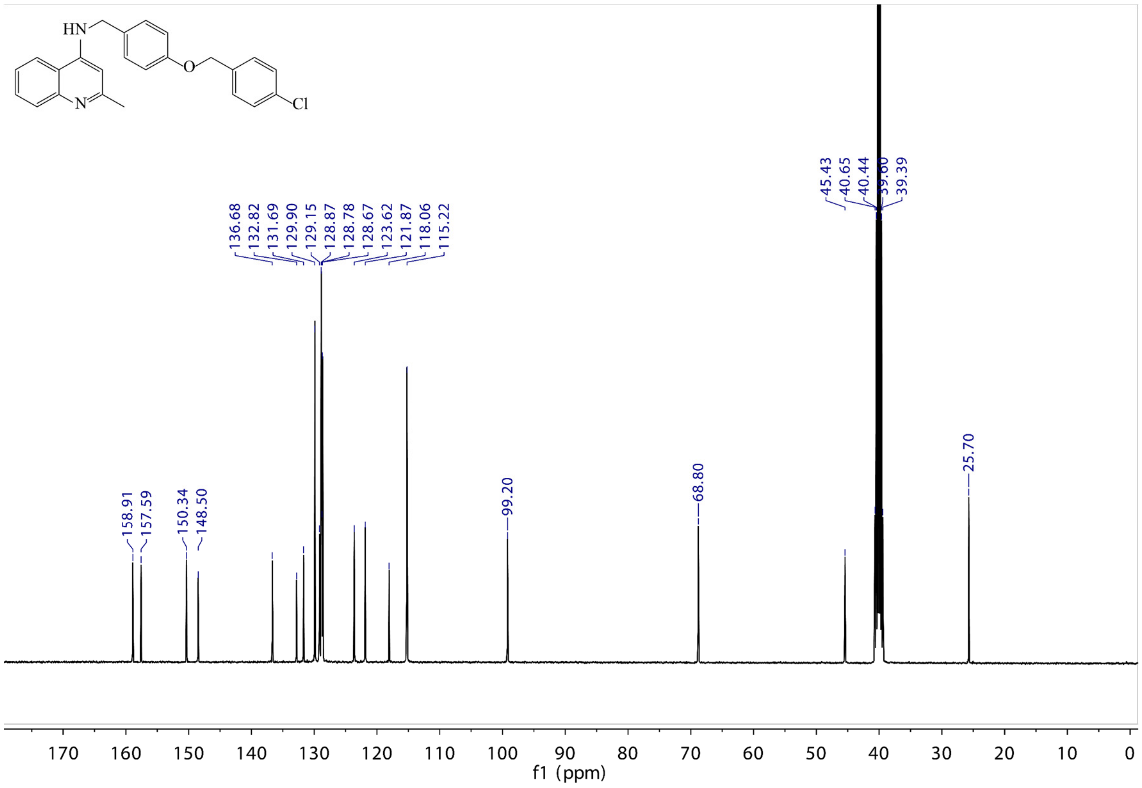

Figure S4: ^{13}C spectrum **9b** of in DMSO-d_6.

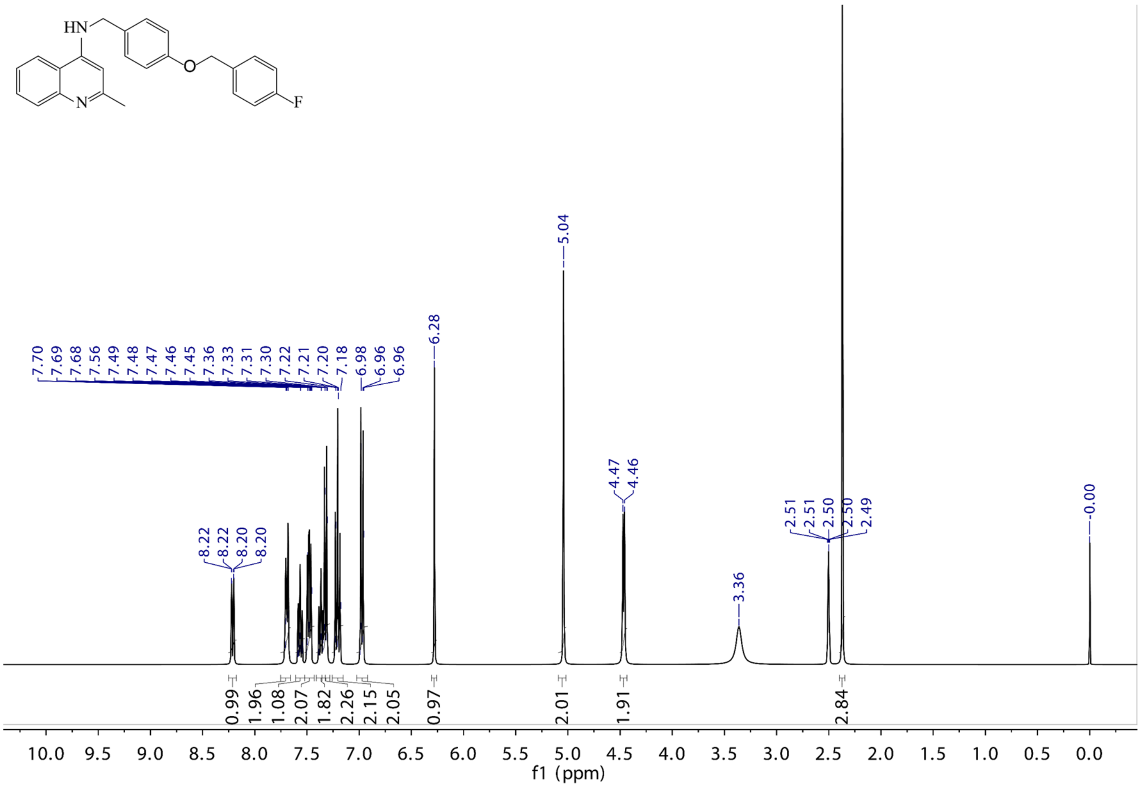

Figure S5: ^{1}H spectrum of **9c** in DMSO-*d₆*.

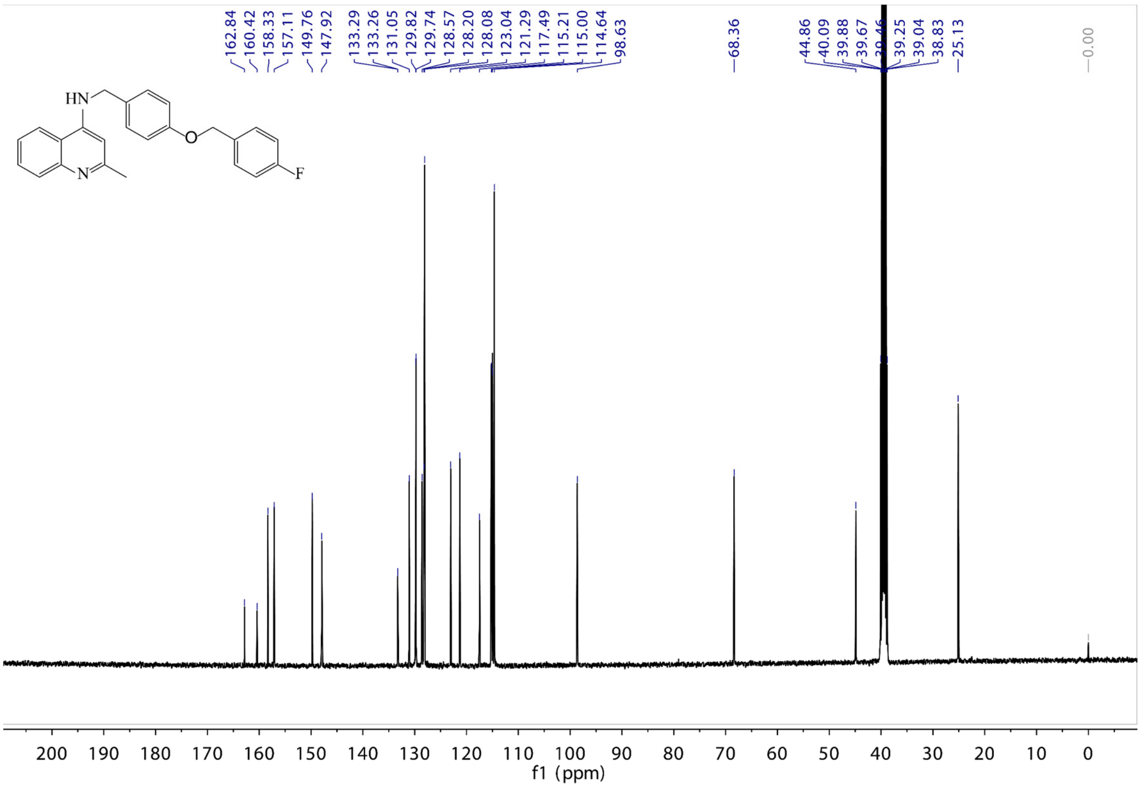

Figure S6: ^{13}C spectrum of **9c** in DMSO-*d₆*.

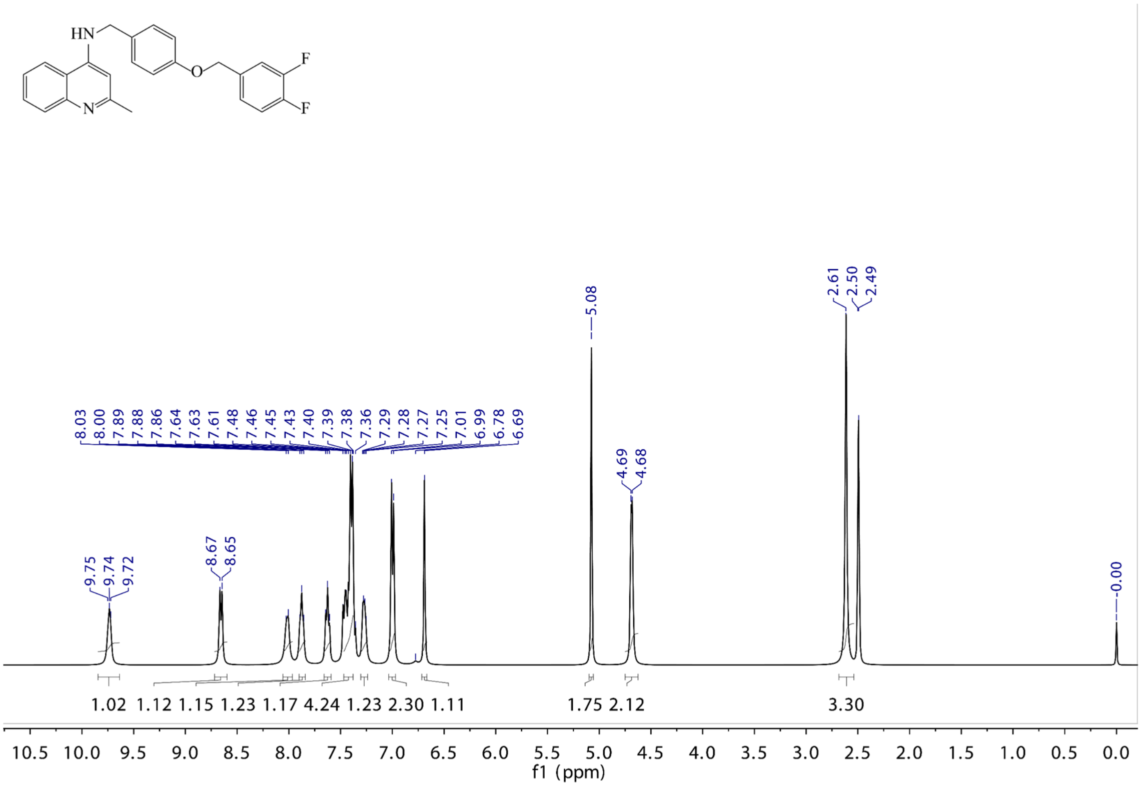

Figure S7: ^{1}H spectrum of **9d** in DMSO-d_6.

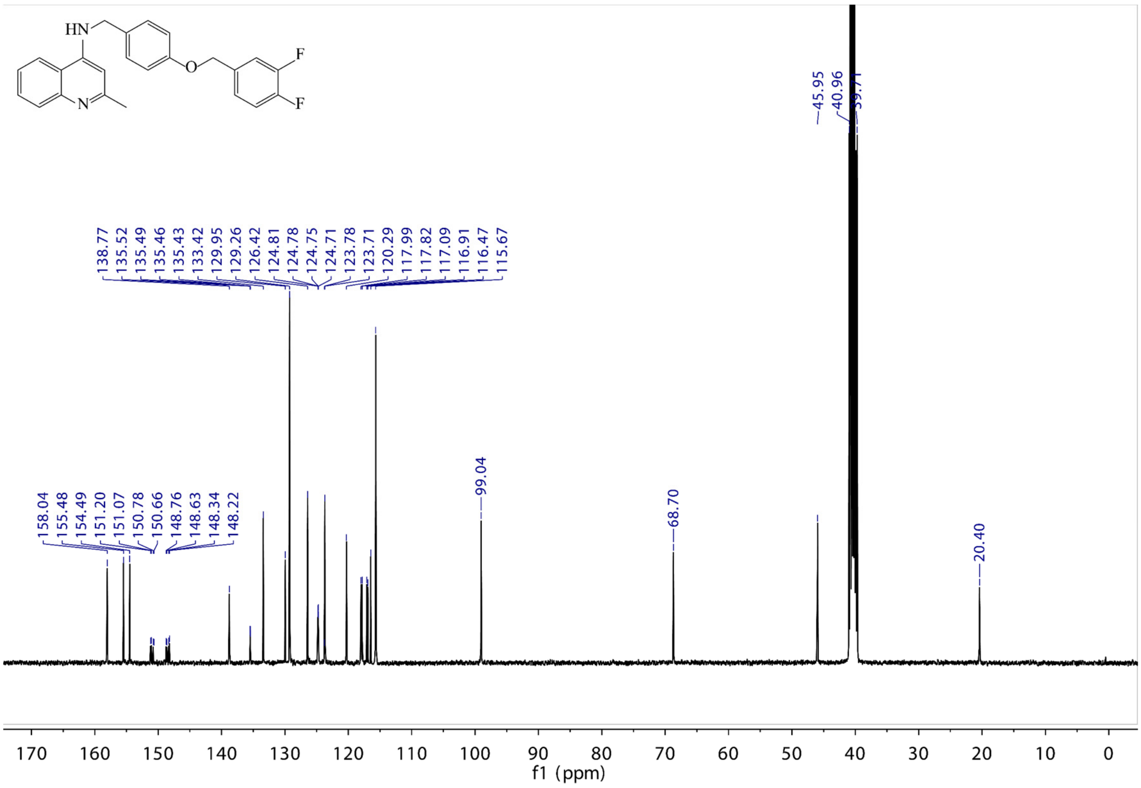

Figure S8: ^{13}C spectrum of **9d** in DMSO-d_6.

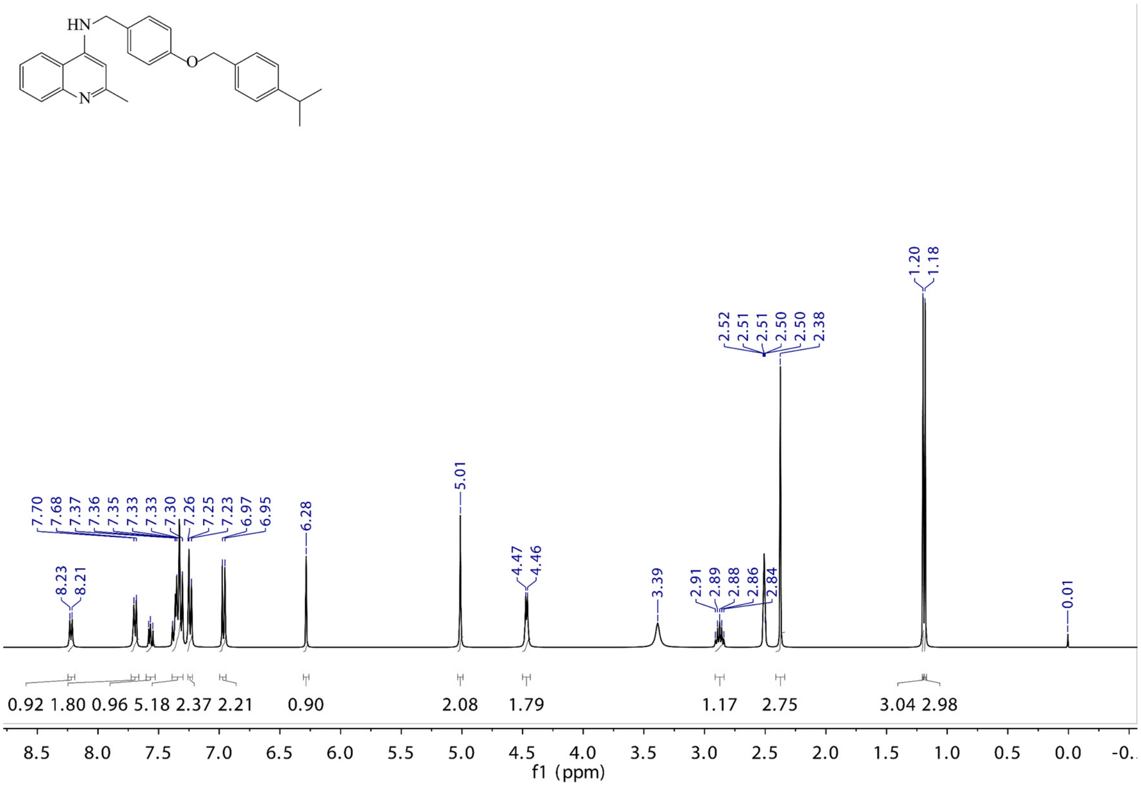

Figure S9: ^{1}H spectrum of **9e** in DMSO-d_6.

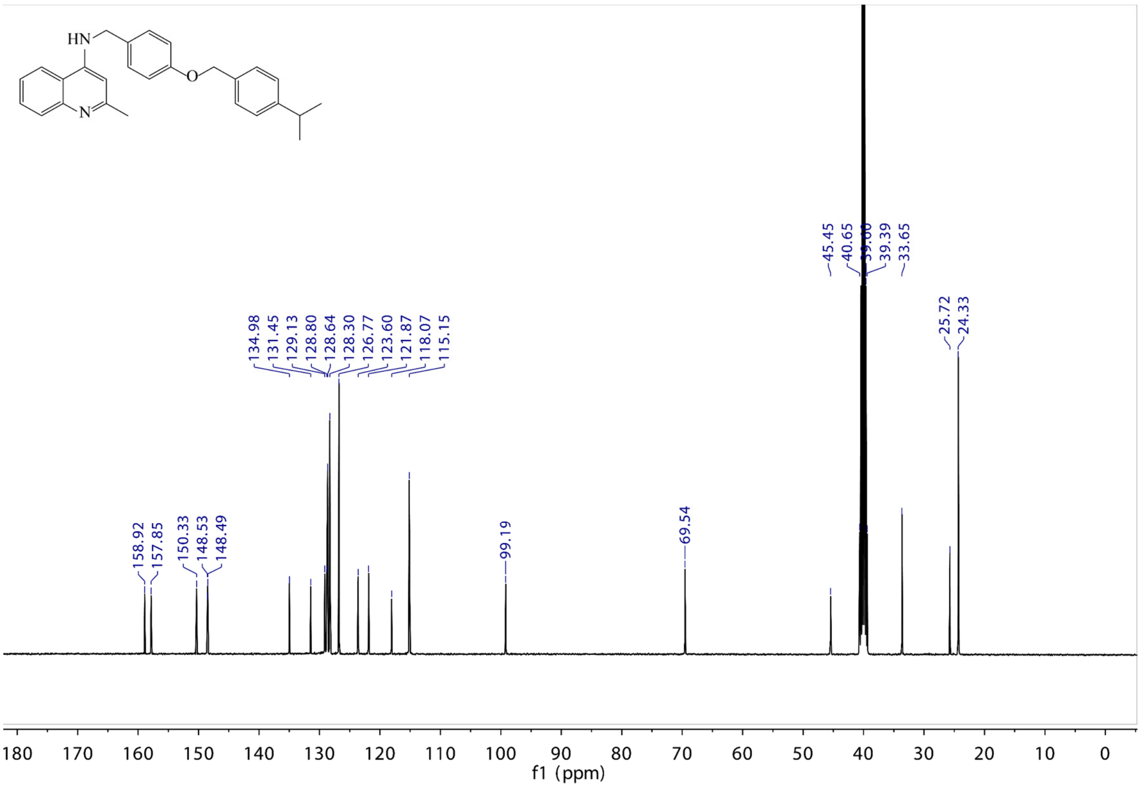

Figure S10: ^{13}C spectrum of **9e** in DMSO-d_6.

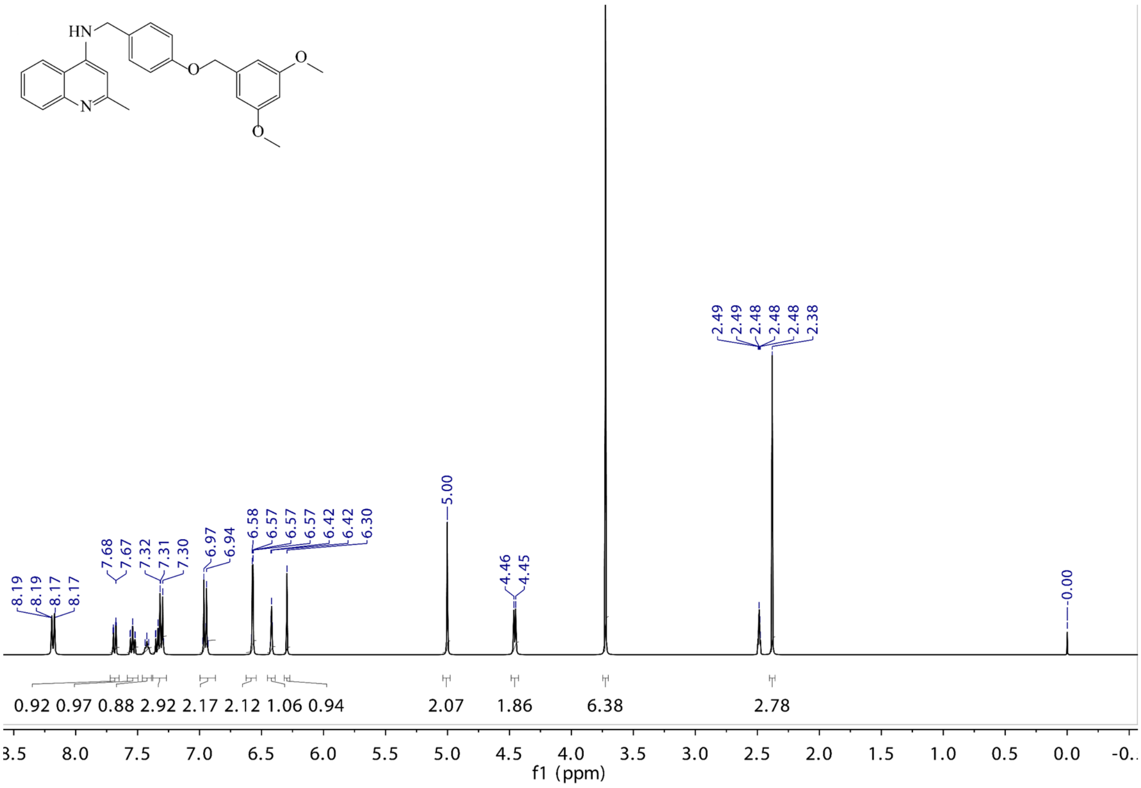

Figure S11: ^{1}H spectrum of **9f** in DMSO-*d6*.

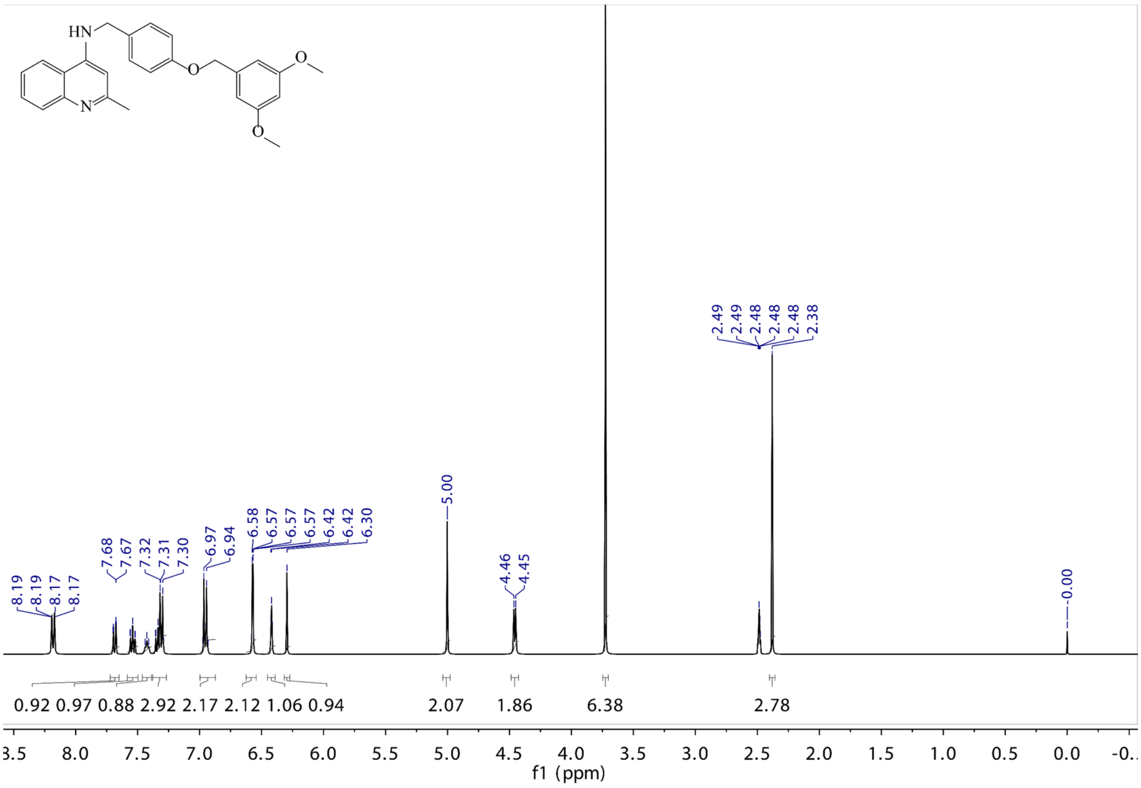

Figure S12: ^{13}C spectrum of **9f** in DMSO-*d6*.

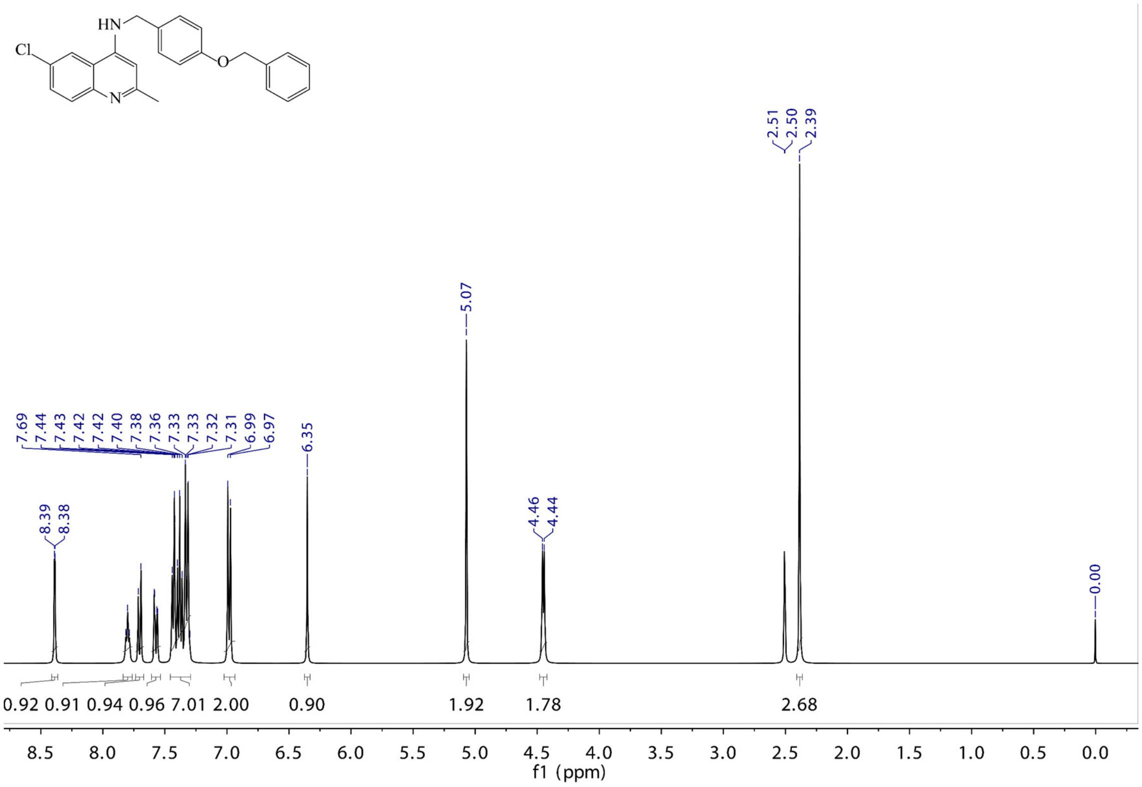

Figure S13: ^{1}H spectrum of **9g** in DMSO-*d*6.

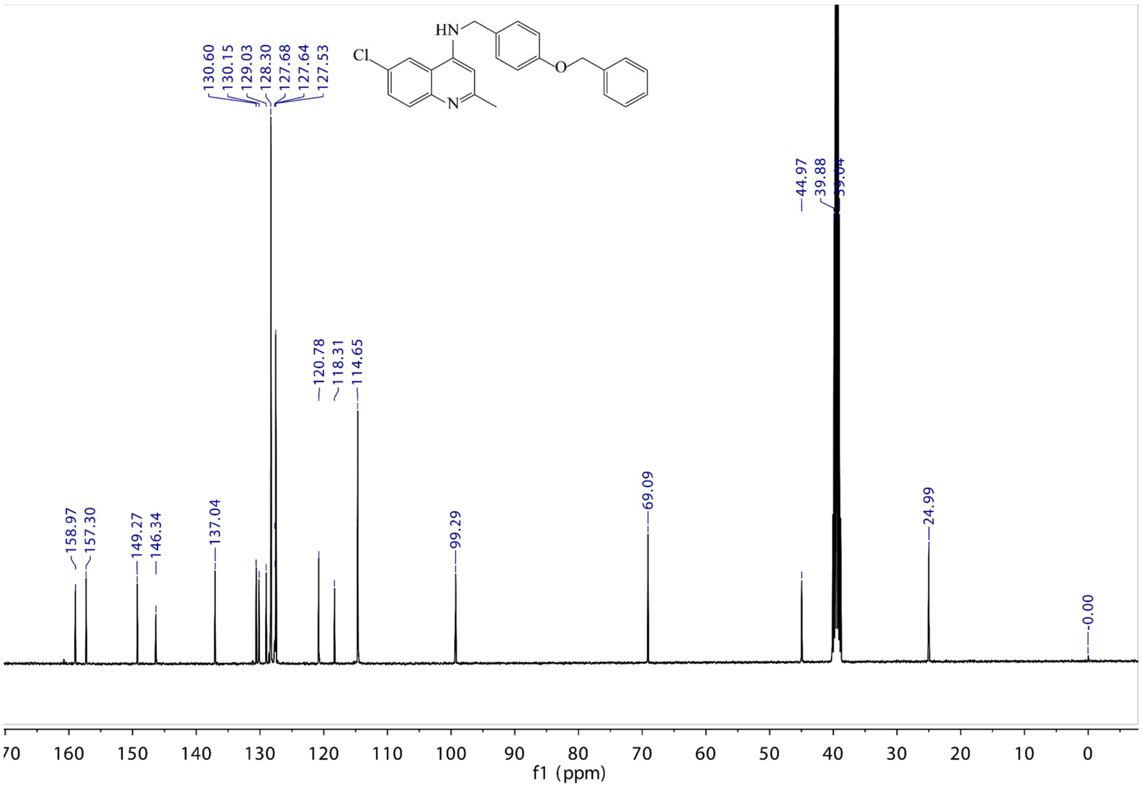

Figure S14: ^{13}C spectrum of **9g** in DMSO-*d*6.

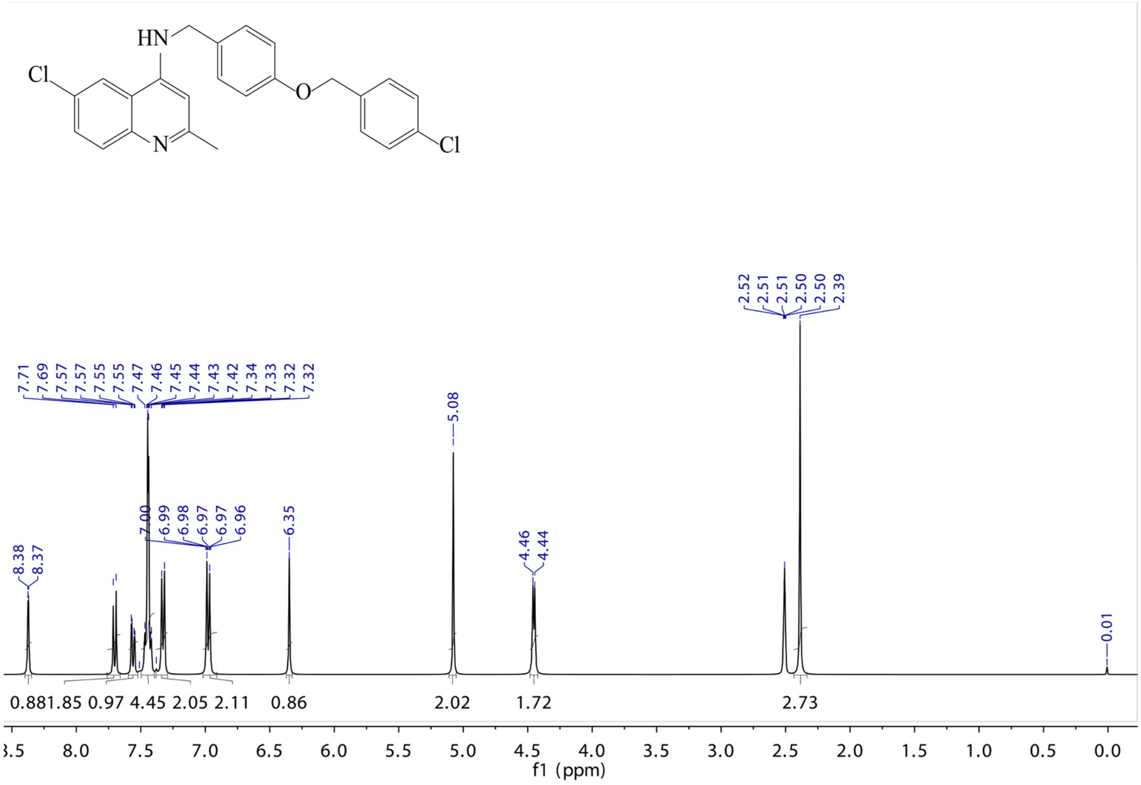

Figure S15: ^{1}H spectrum of **9h** in DMSO-*d6*.

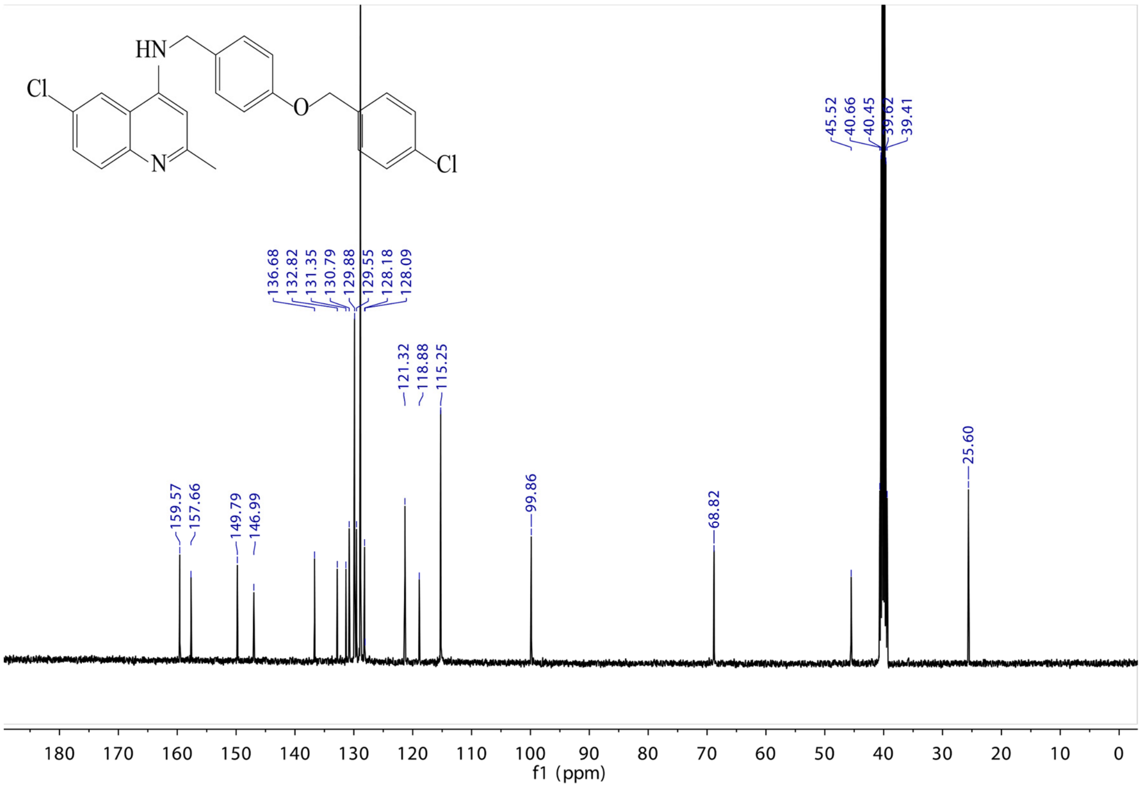

Figure S16: ^{13}C spectrum of **9h** in DMSO-*d6*.

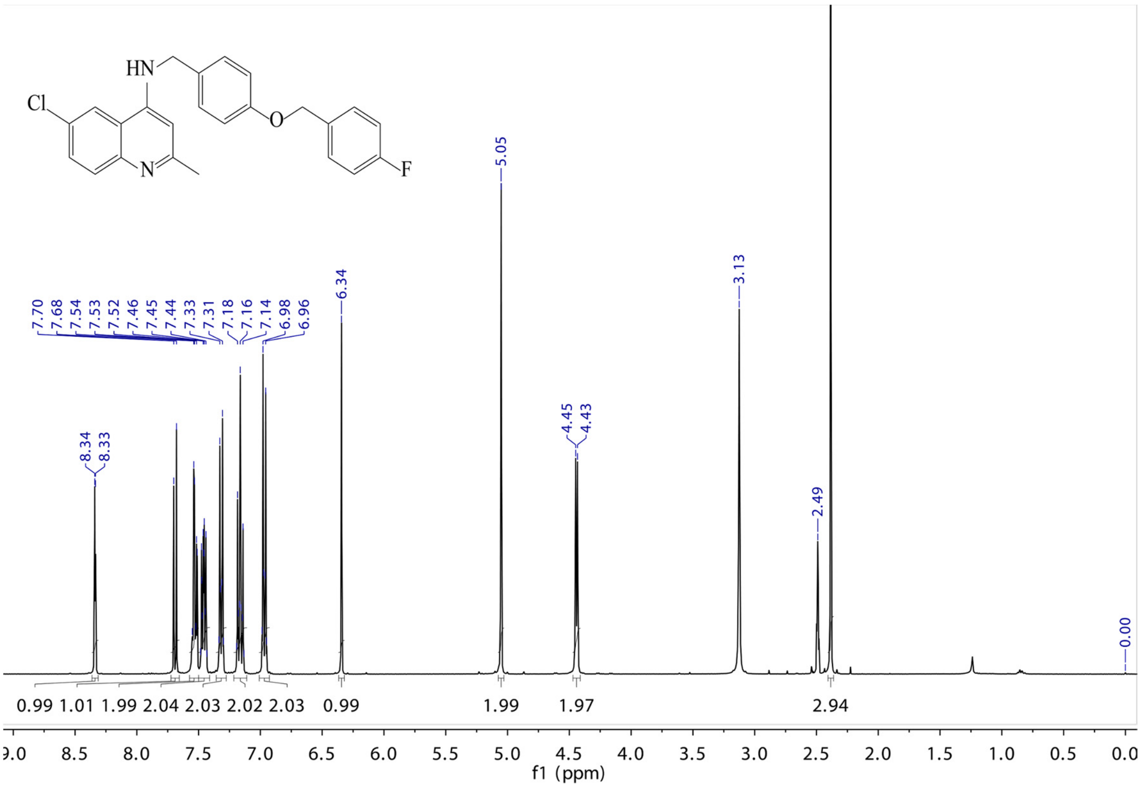

Figure S17: ^{1}H spectrum of **9i** in DMSO-*d₆*.

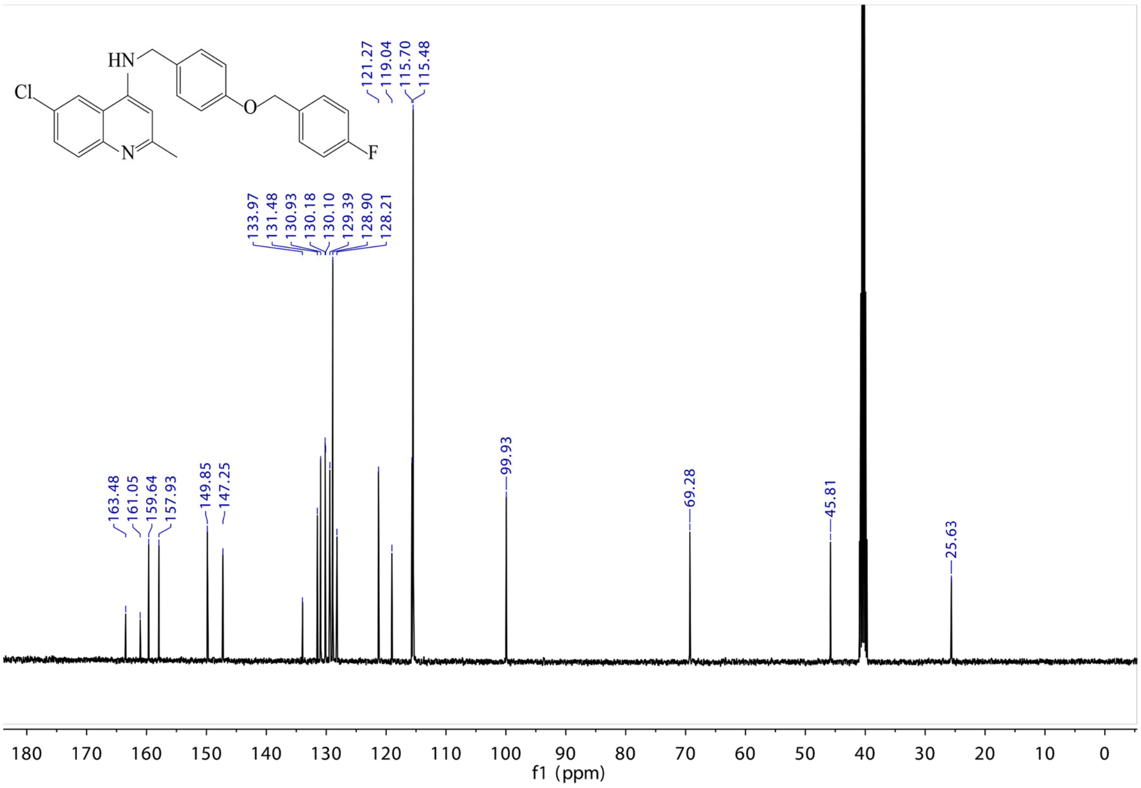

Figure S18: ^{13}C spectrum of **9i** in DMSO-*d₆*.

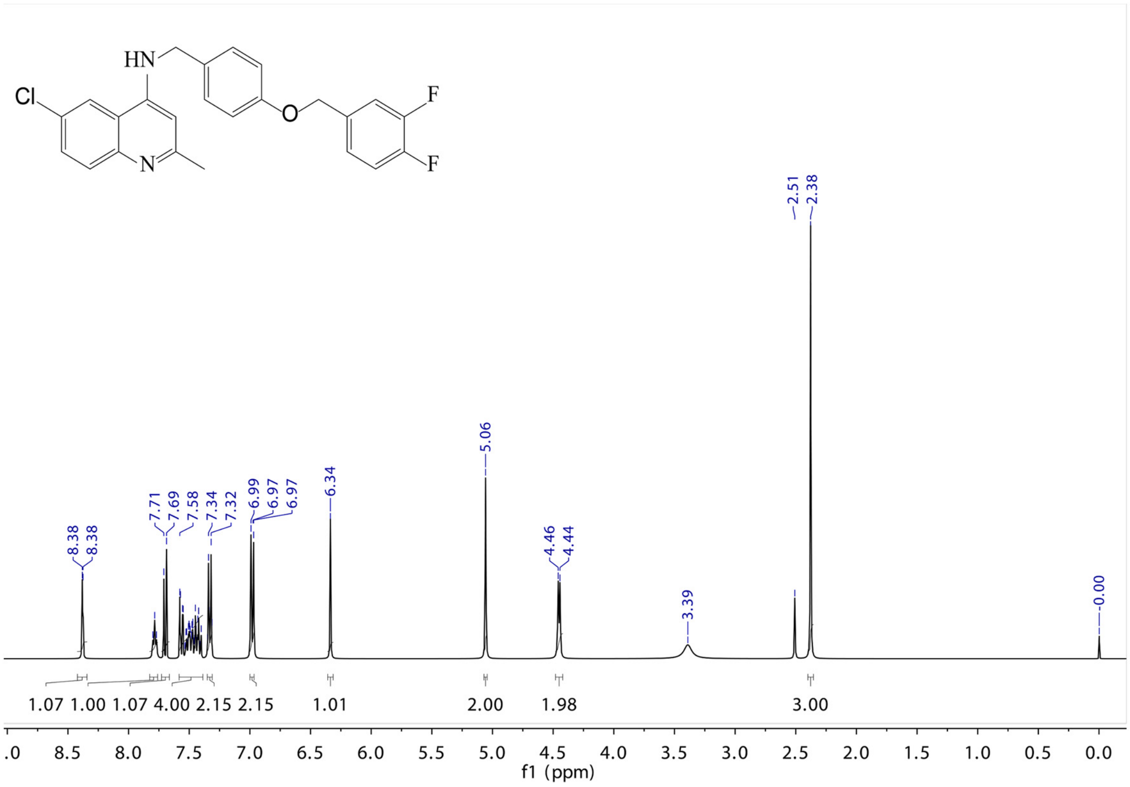

Figure S19: ^{1}H spectrum of **9j** in DMSO-*d₆*.

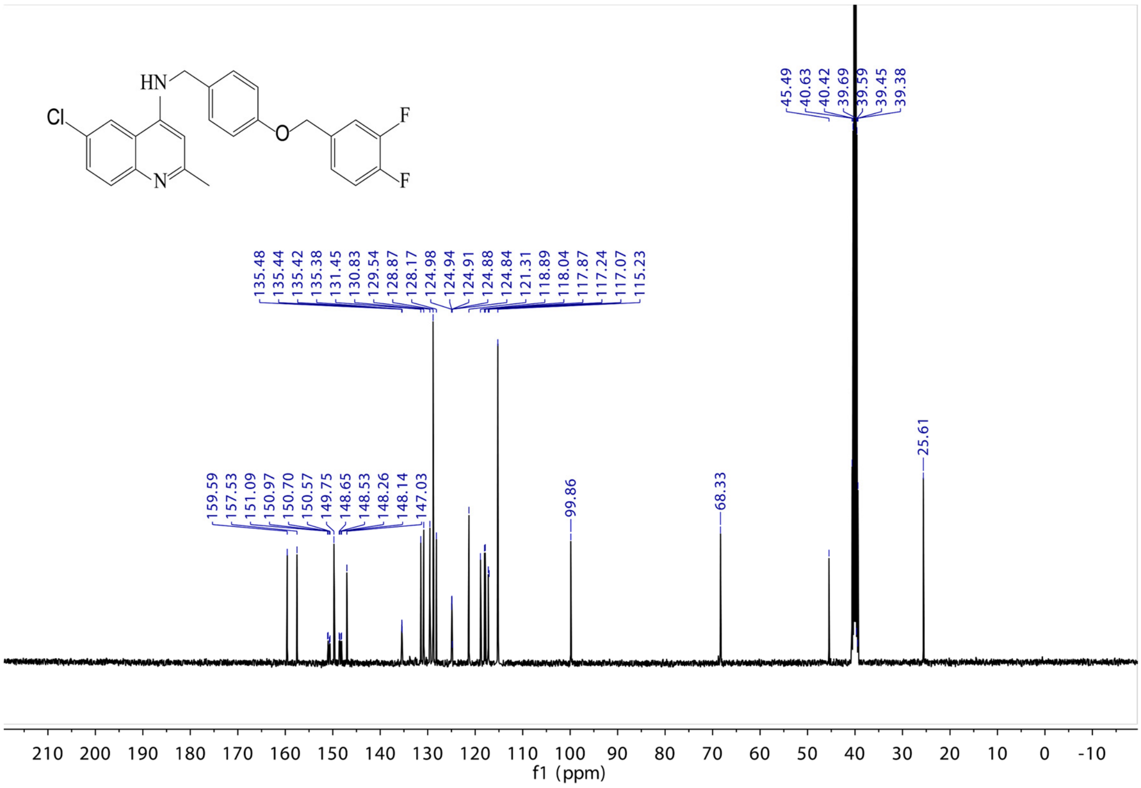

Figure S20: ^{13}C spectrum of **9j** in DMSO-*d₆*.

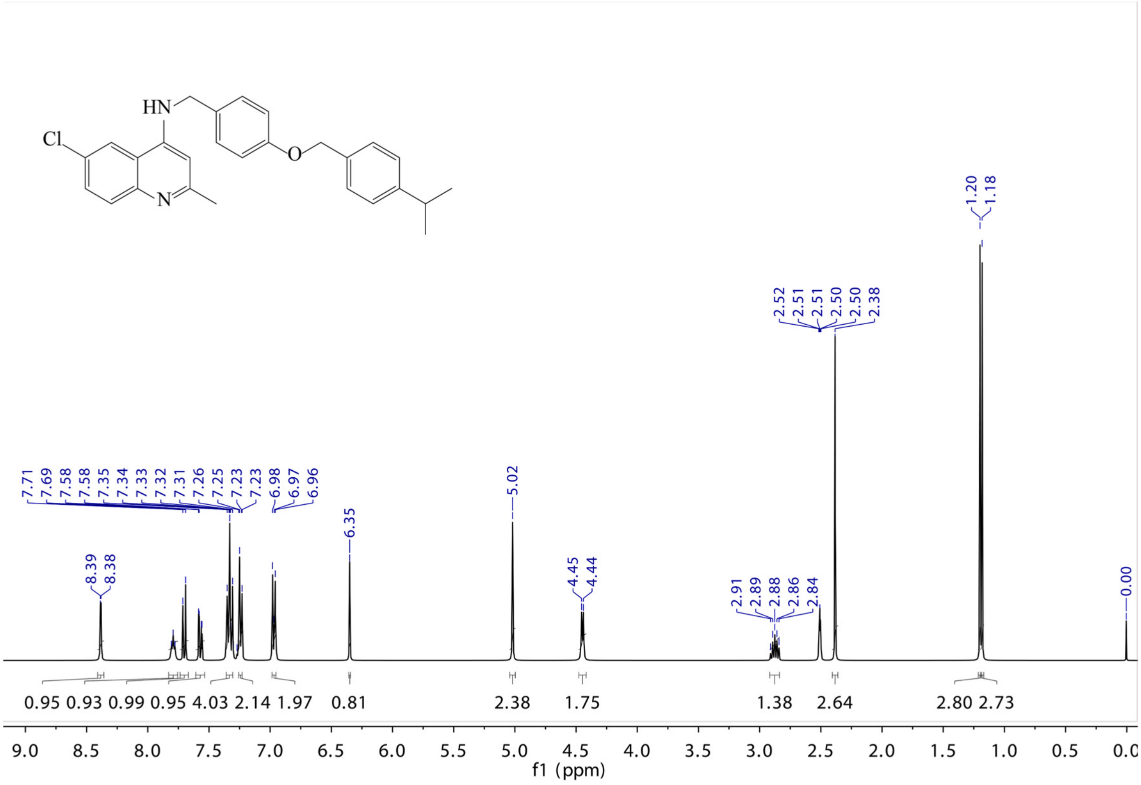

Figure S21: ^{1}H spectrum of **9k** in DMSO-*d*$_6$.

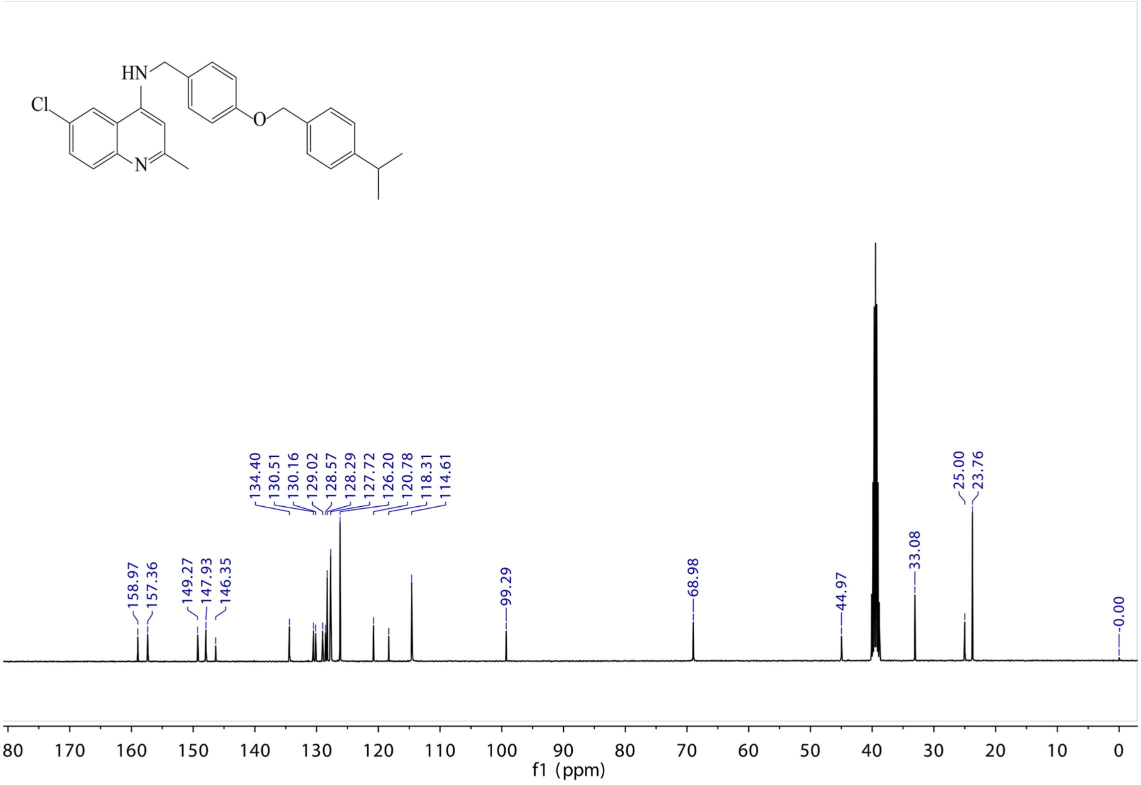

Figure S22: ^{13}C spectrum of **9k** in DMSO-*d*$_6$.

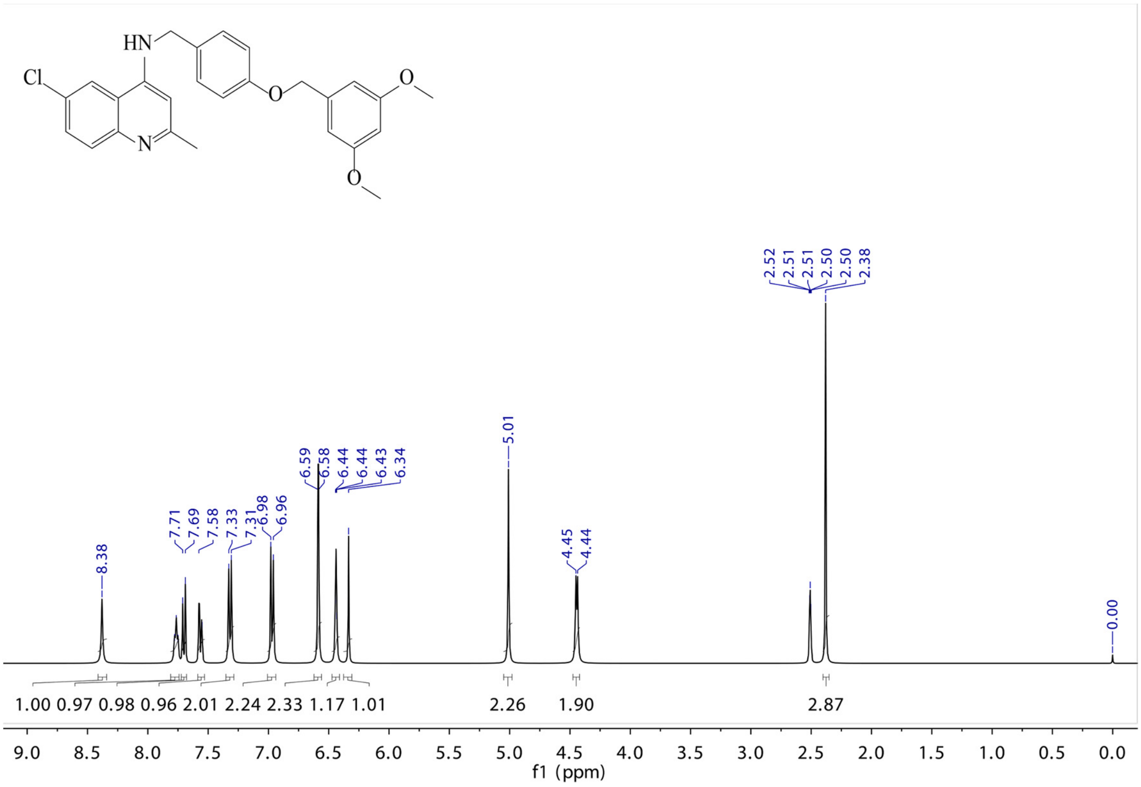

Figure S23: ^{1}H spectrum of **9l** in DMSO-d_6.

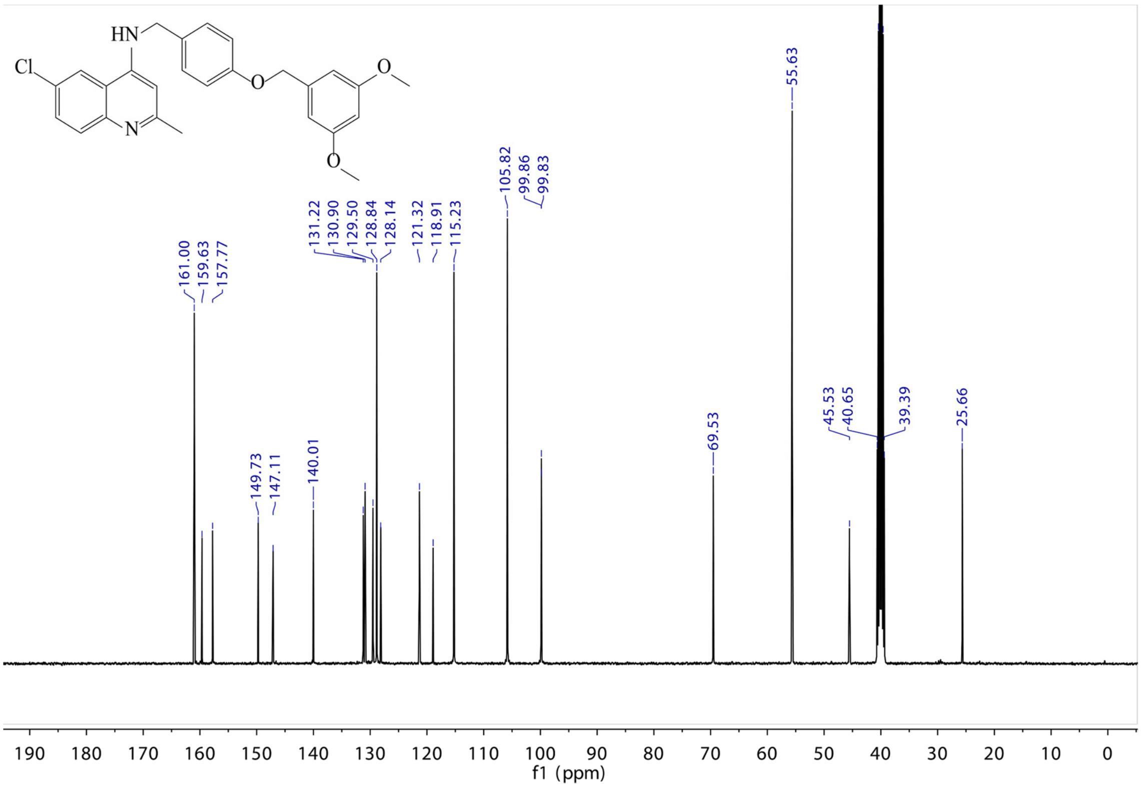

Figure S24: ^{13}C spectrum of **9l** in DMSO-d_6.

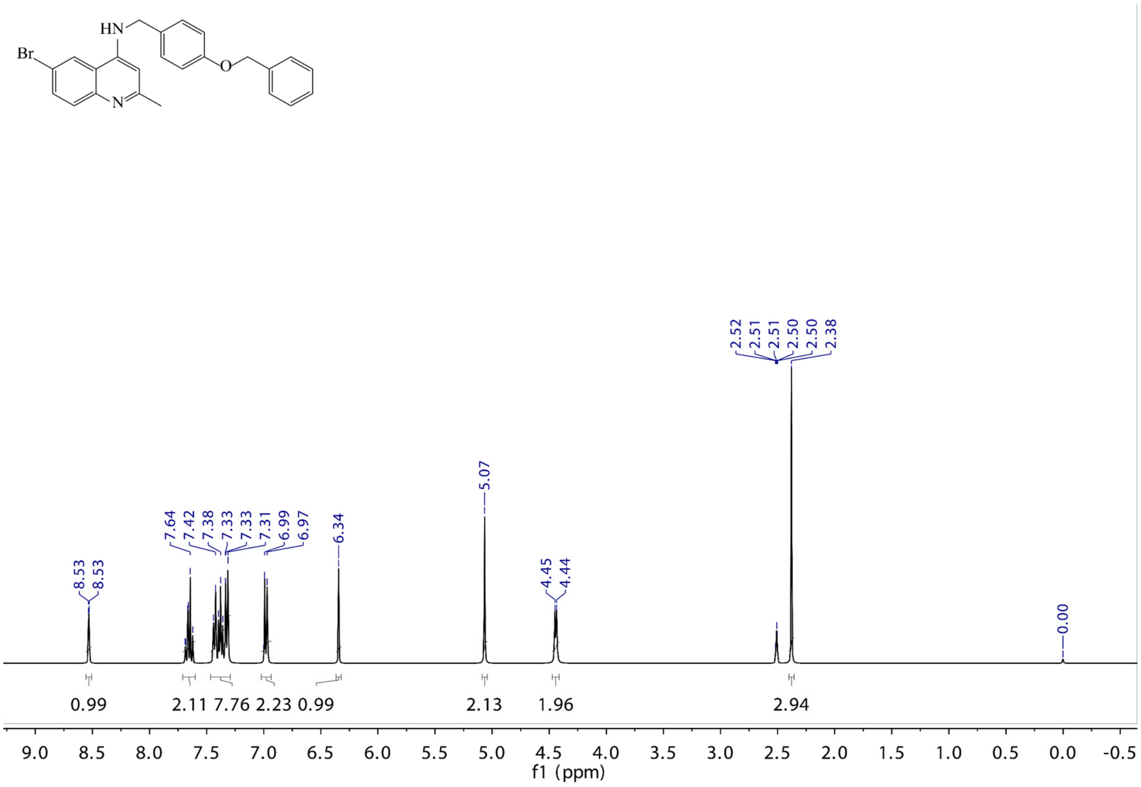

Figure S25: ^{1}H spectrum of **9m** in DMSO-d_6.

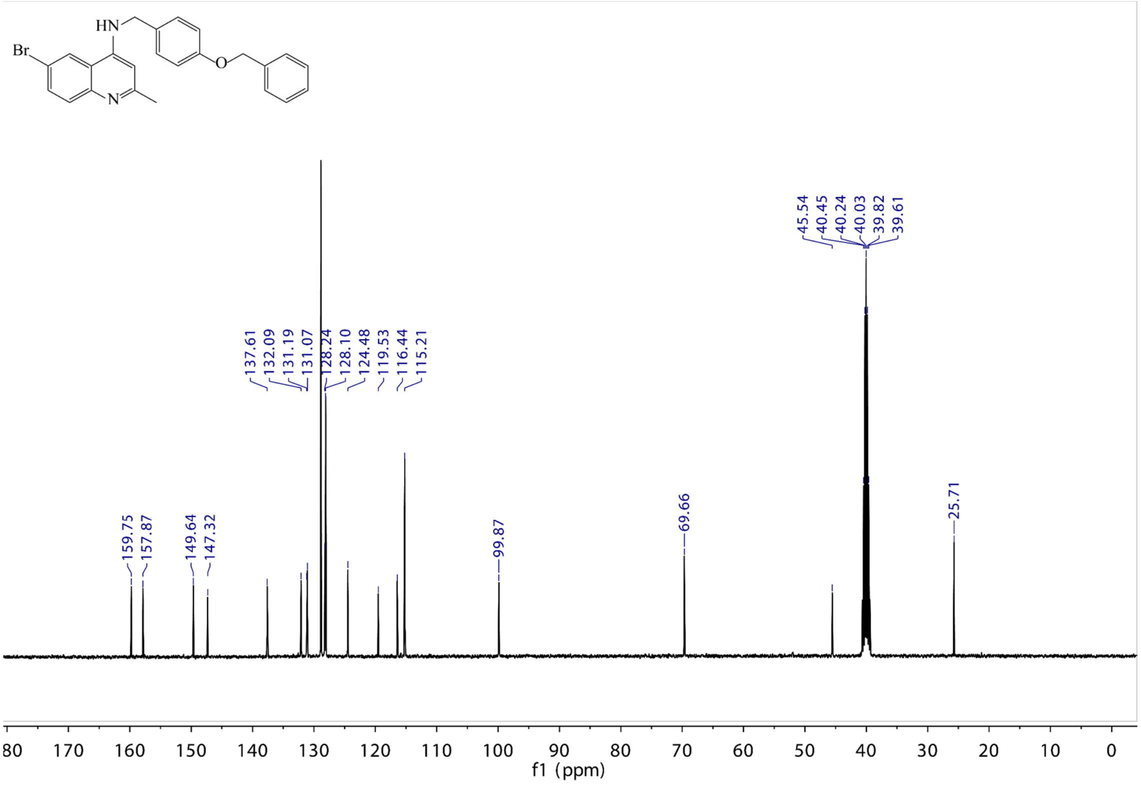

Figure S26: ^{13}C spectrum of **9m** in DMSO-d_6.

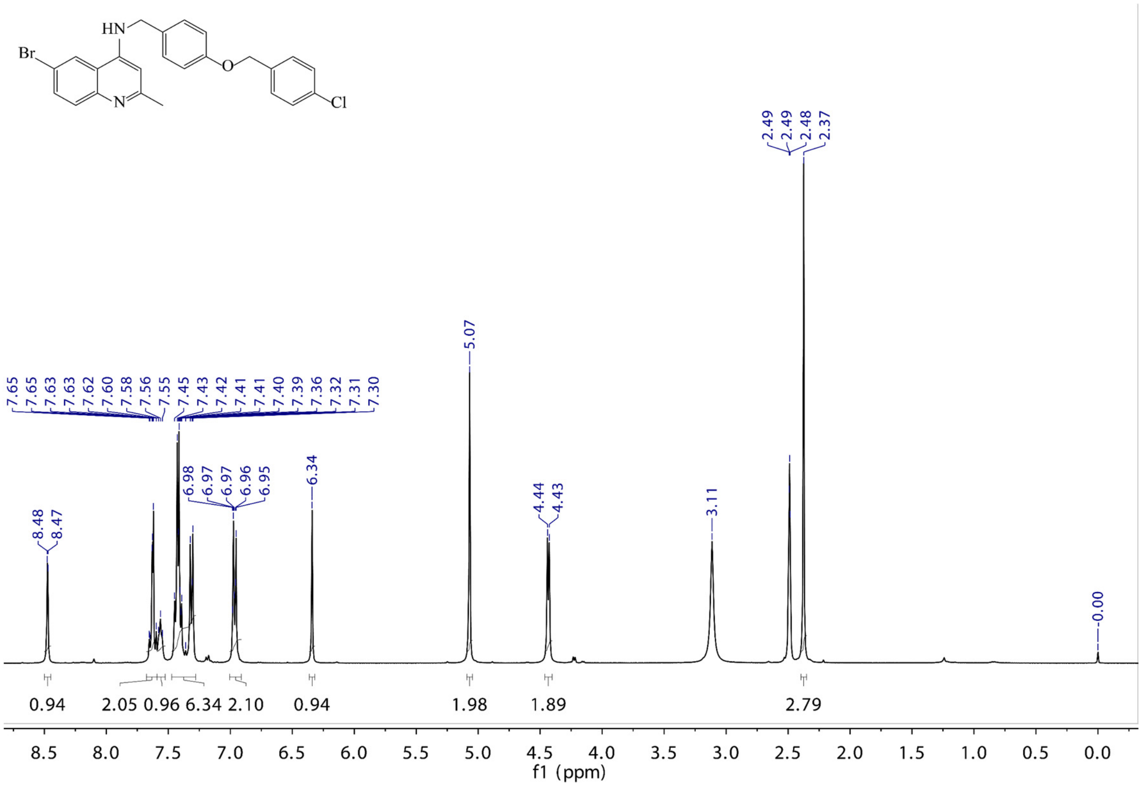

Figure S27: ^{1}H spectrum of **9n** in DMSO-*d*$_6$.

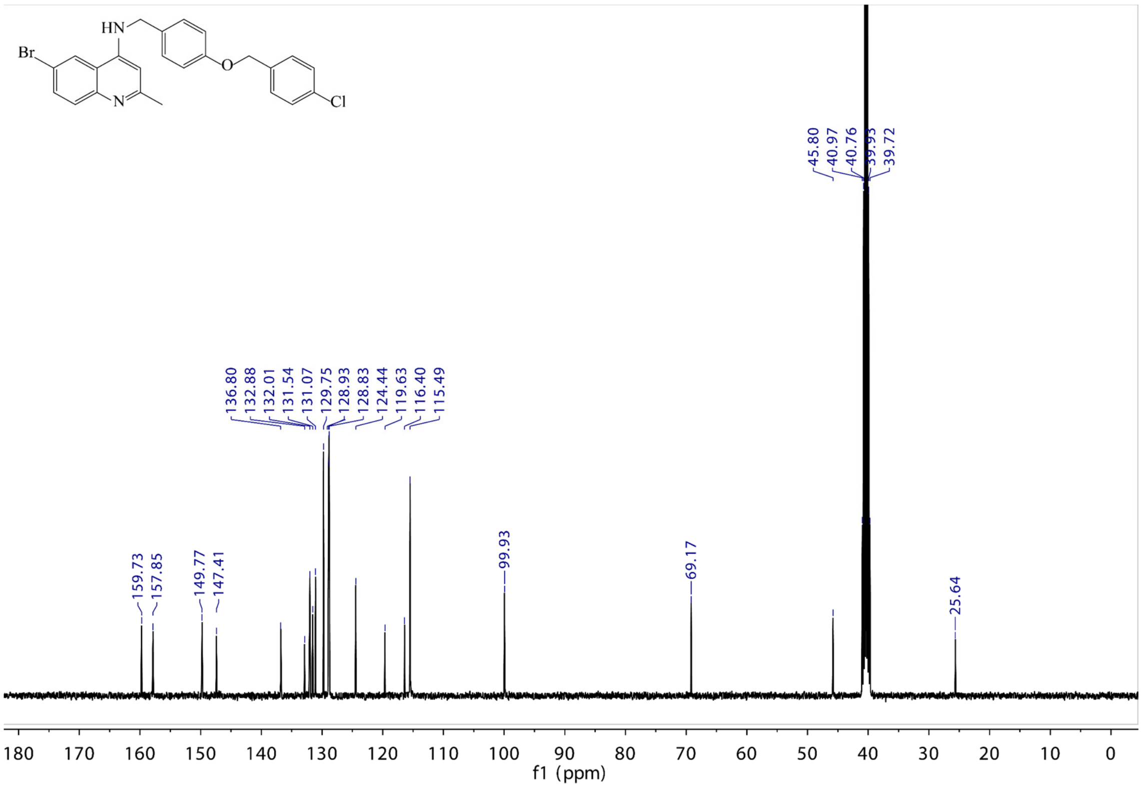

Figure S28: ^{13}C spectrum of **9n** in DMSO–*d6*.

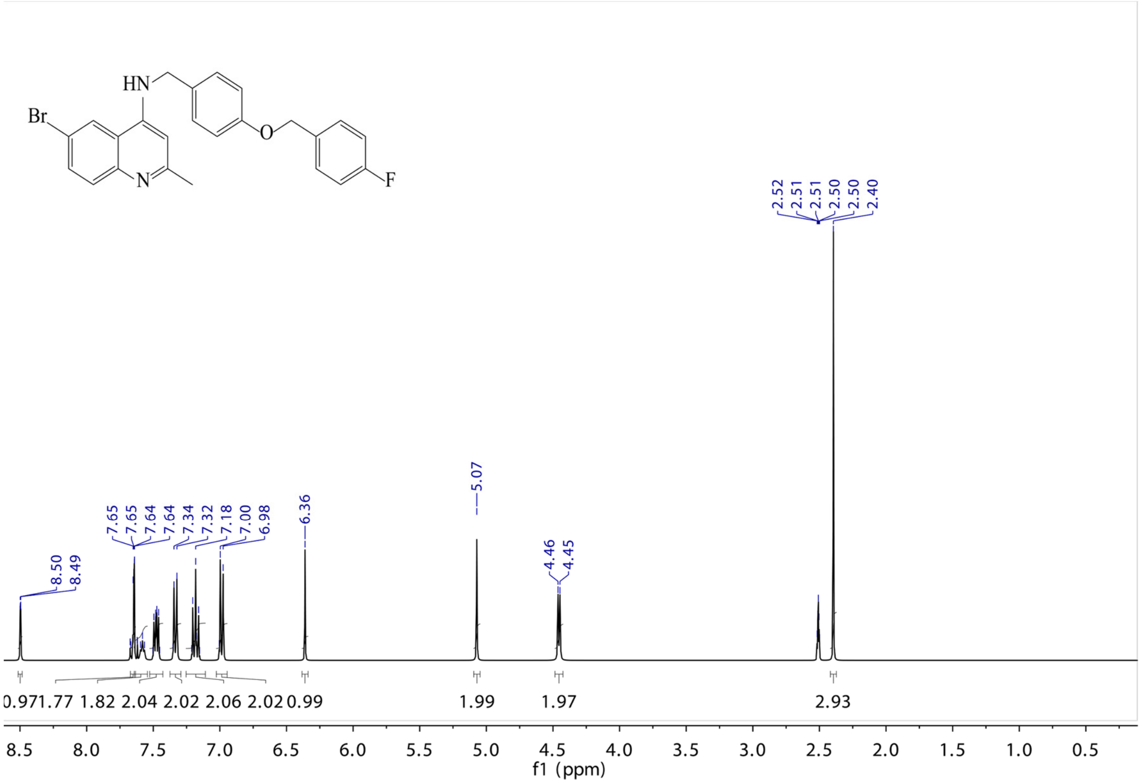

Figure S29: 1H spectrum of 9o in DMSO-d_6.

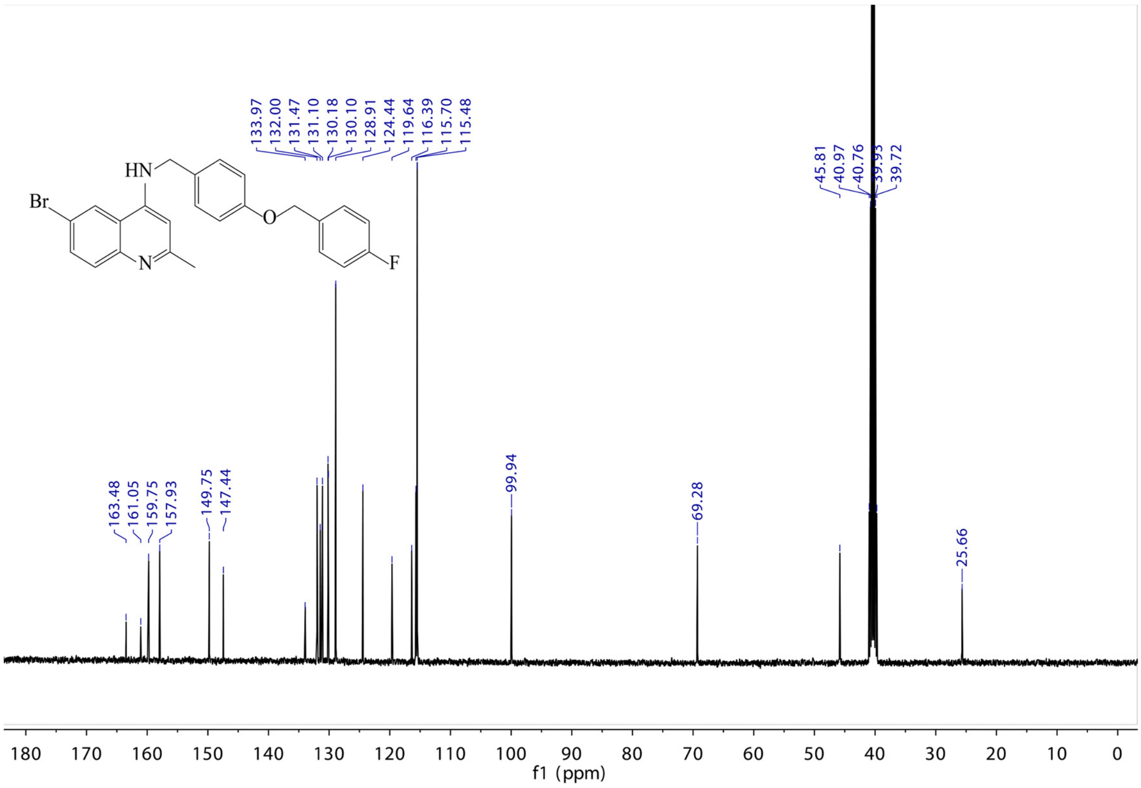

Figure S30: ^{13}C spectrum of **9o** in DMSO-d_6.

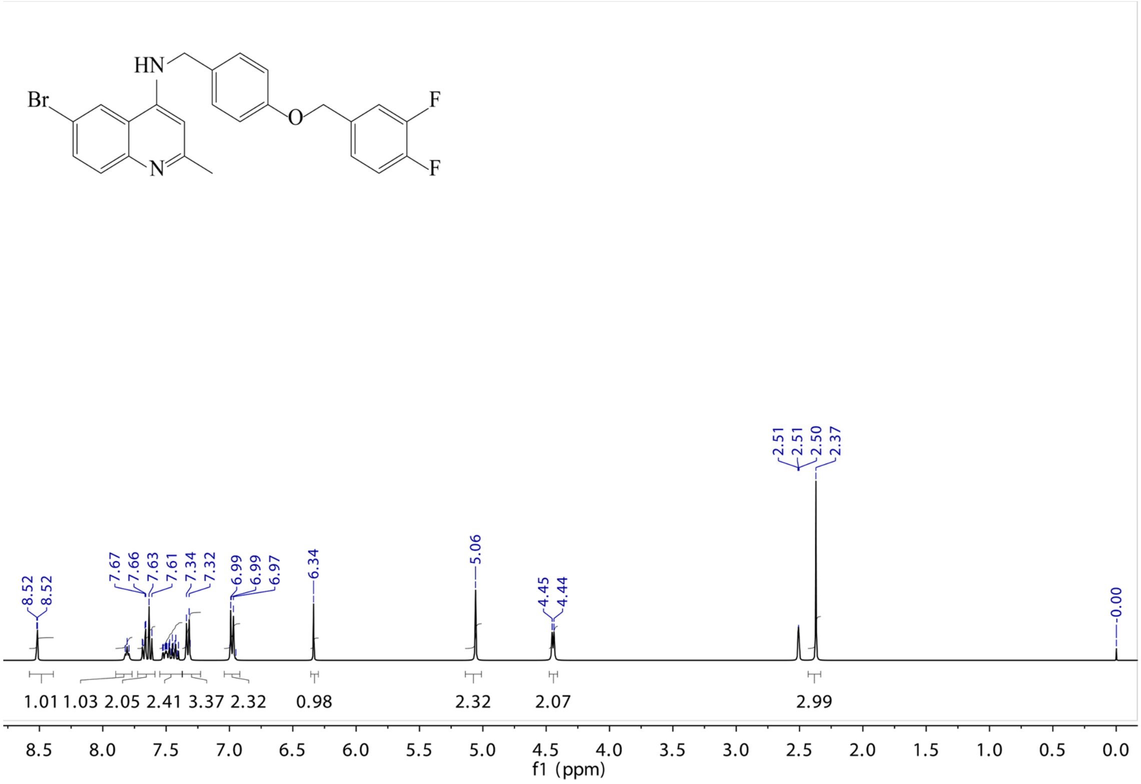

Figure S31: ^{1}H spectrum of **9p** in DMSO-*d₆*.

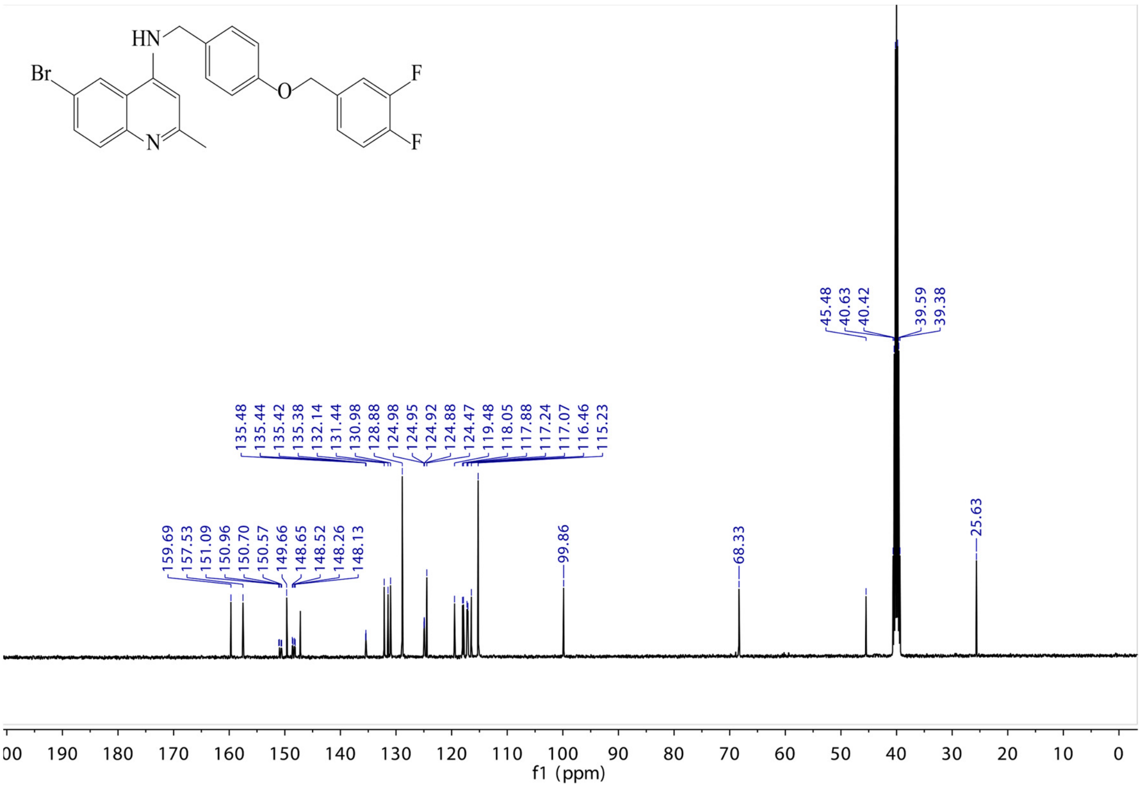

Figure S32: ^{13}C spectrum of **9p** in DMSO-*d₆*.

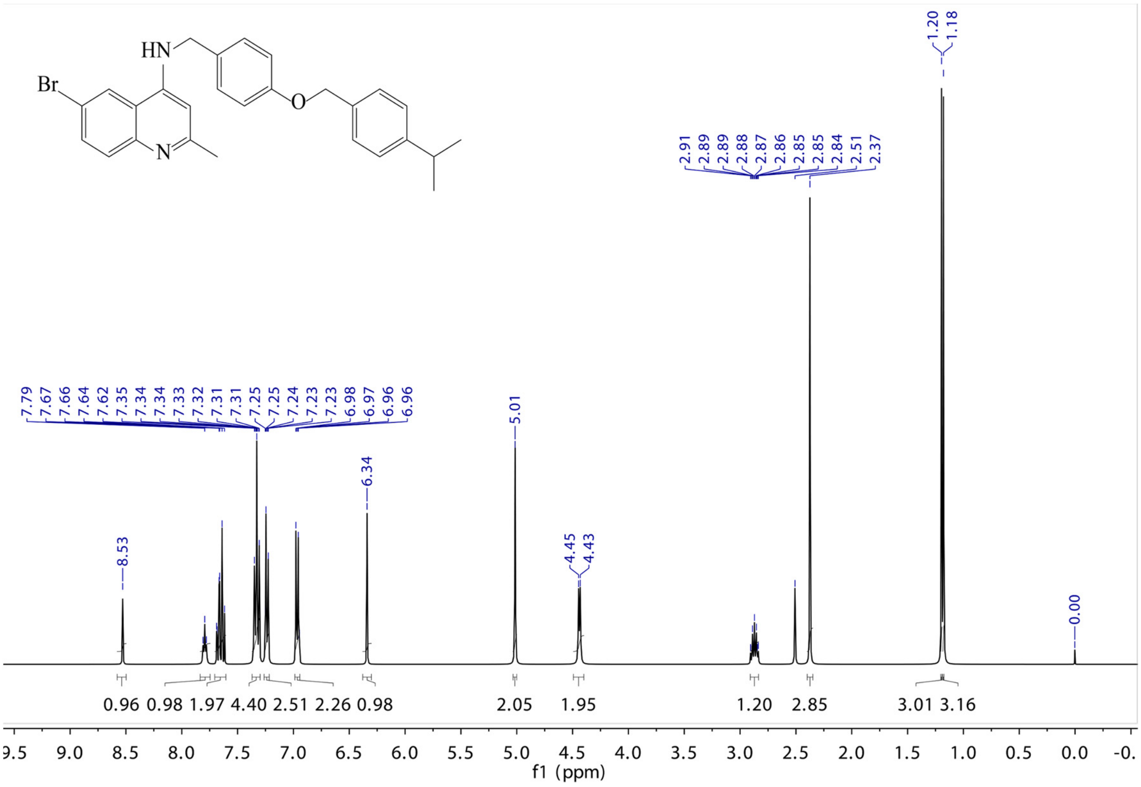

Figure S33: ^{1}H spectrum of **9q** in DMSO-*d₆*.

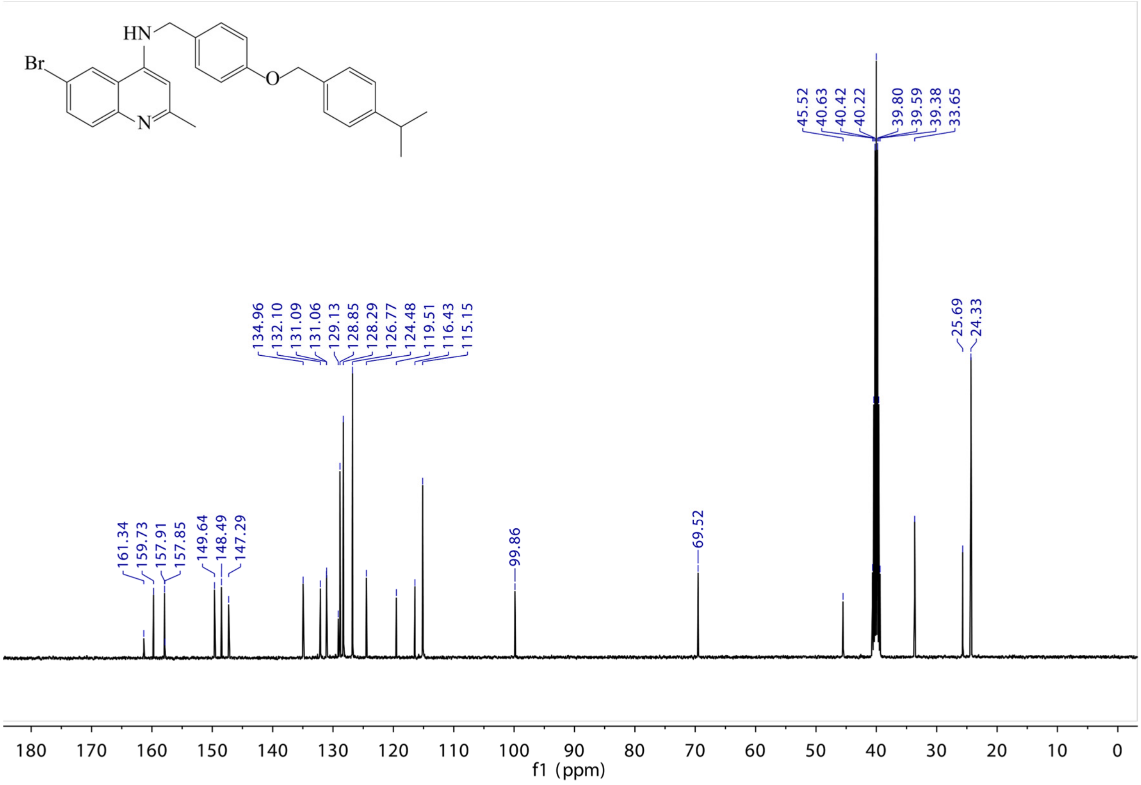

Figure S34: ^{13}C spectrum of **9q** in DMSO-*d₆*.

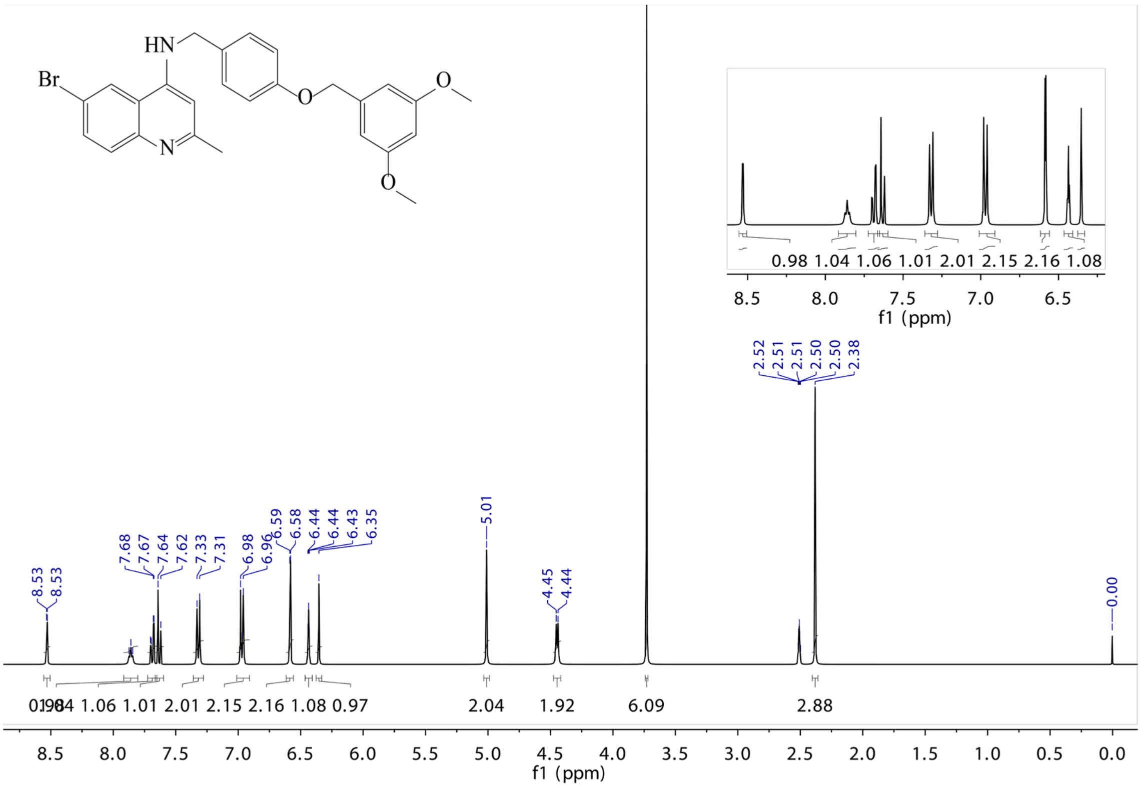

Figure S35: ^{1}H spectrum of **9r** in DMSO-*d₆*.

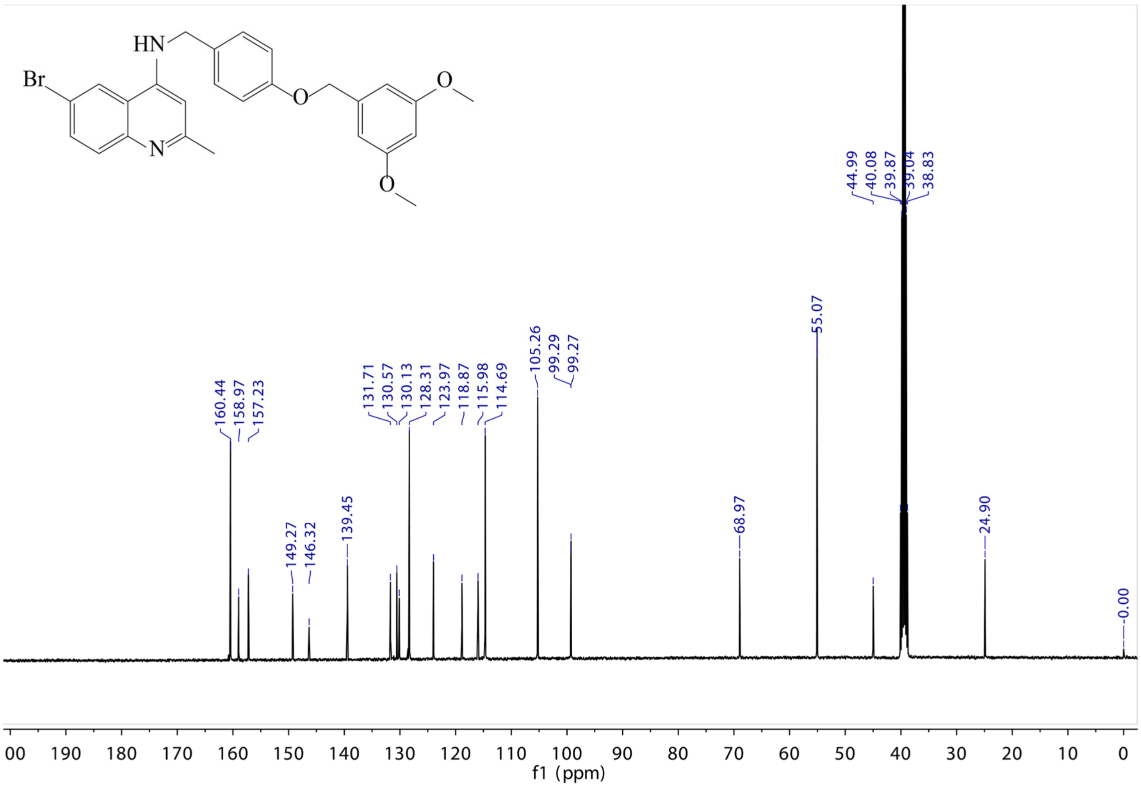

Figure S36: ^{13}C spectrum of **9r** in DMSO-*d₆*.

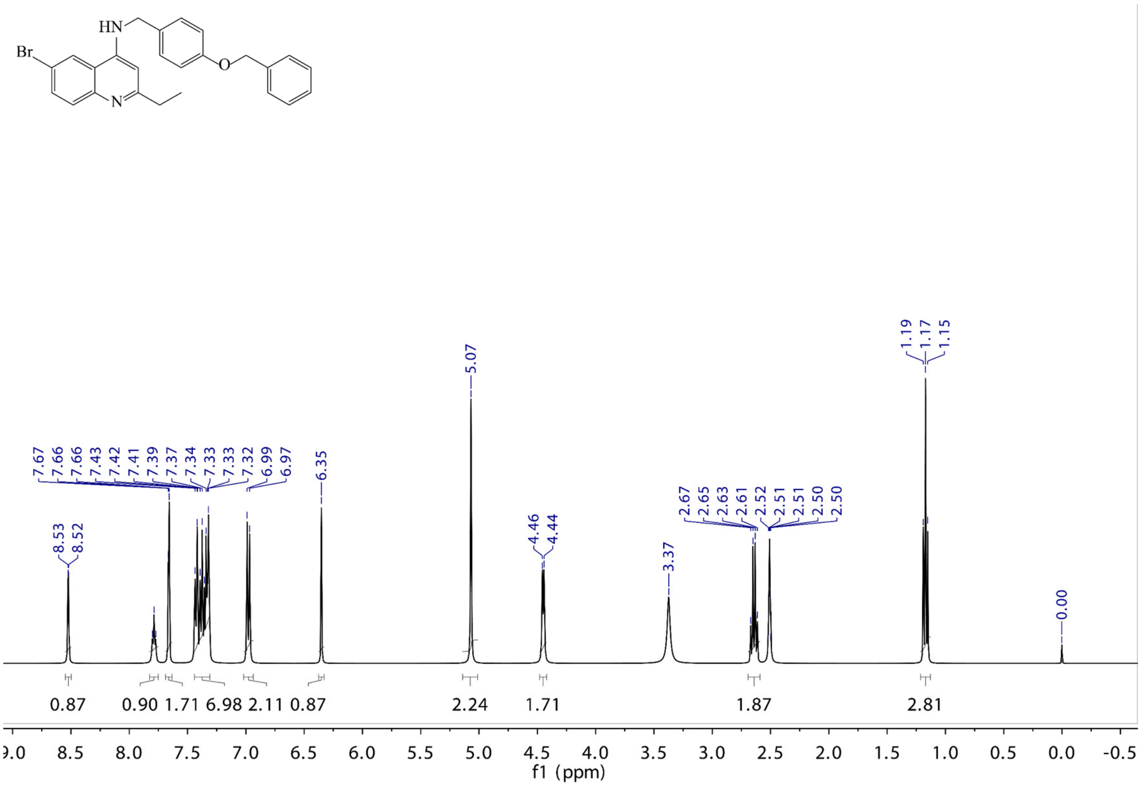

Figure S37: ^{1}H spectrum of **9s** in DMSO-*d6*.

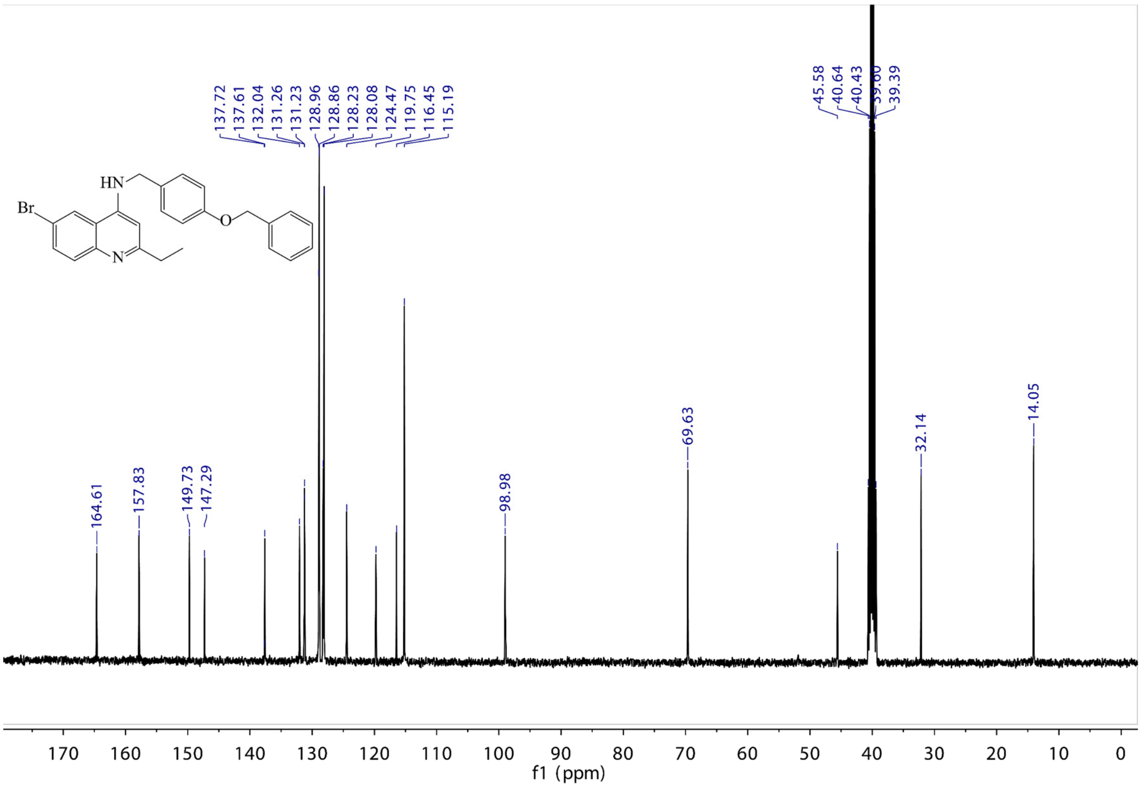

Figure S38: ^{13}C spectrum of **9s** in DMSO-*d6*.

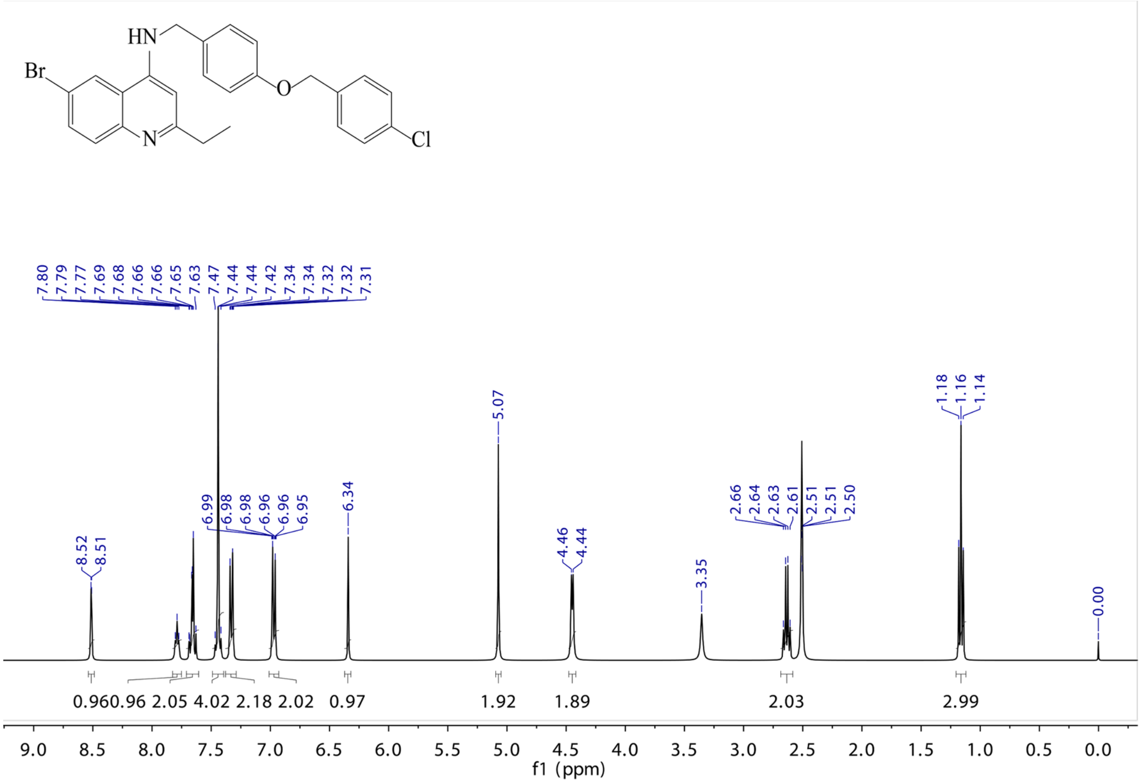

Figure S39: ^{1}H spectrum of **9t** in DMSO-d_6.

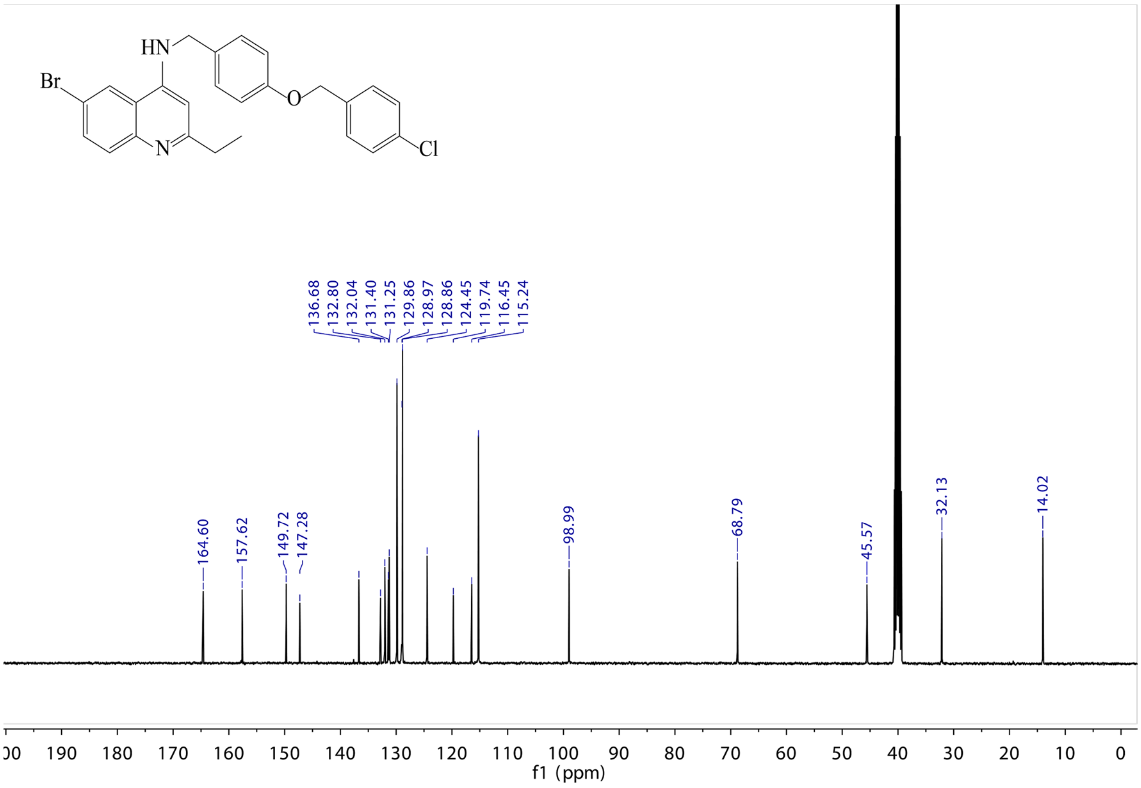

Figure S40: ^{13}C spectrum of **9t** in DMSO-d_6.

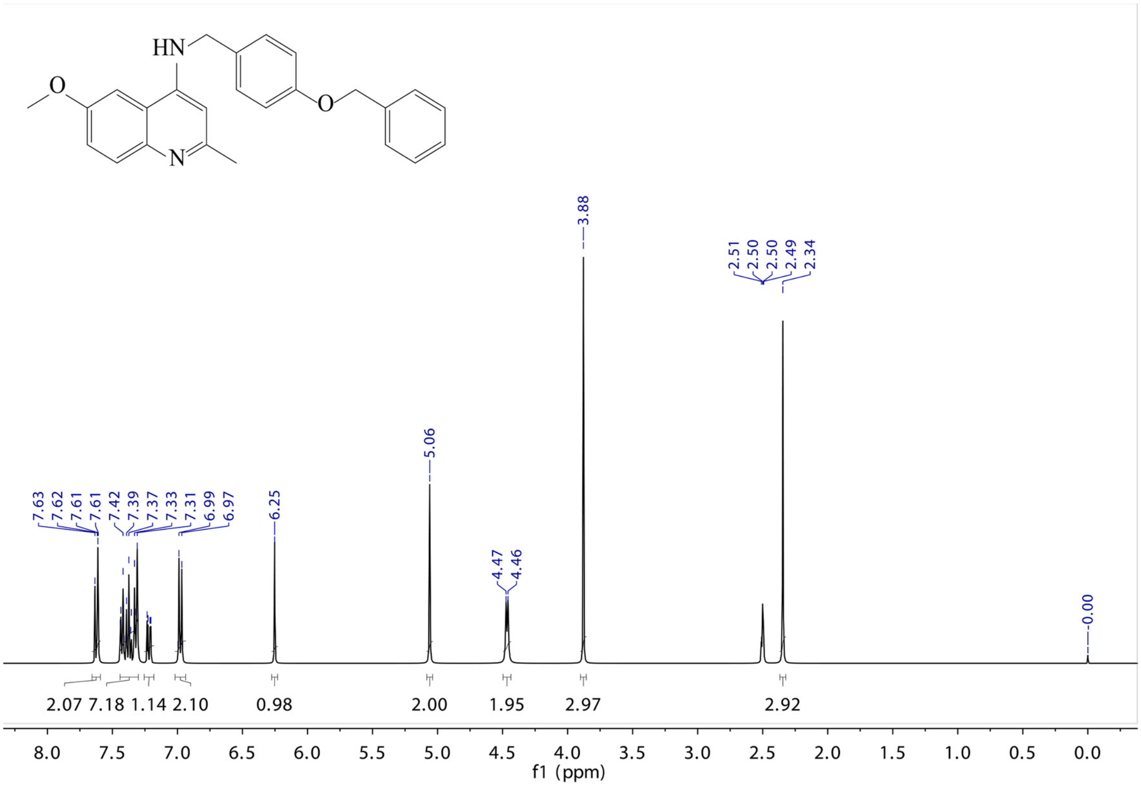

Figure S41: ^{1}H spectrum of **9u** in DMSO-*d6*.

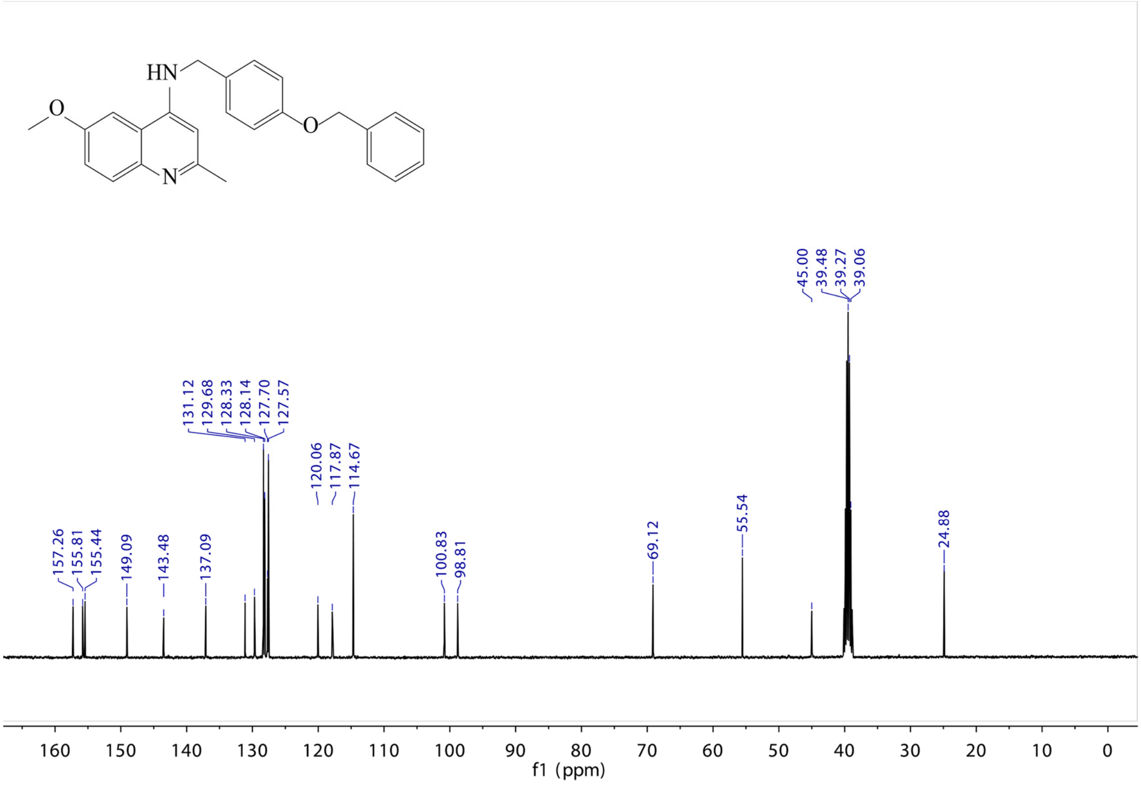

Figure S42: ^{13}C spectrum of **9u** in DMSO-*d6*.

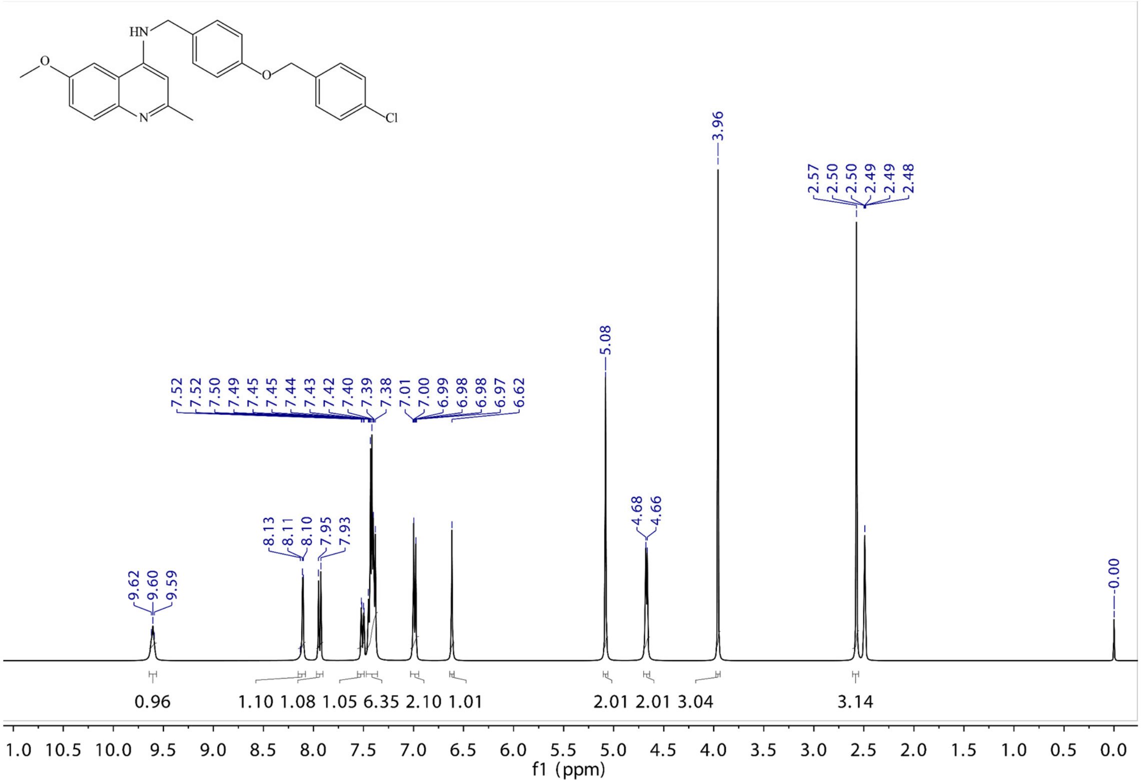

Figure S43: ^{1}H spectrum of **9v** in DMSO-*d6*.

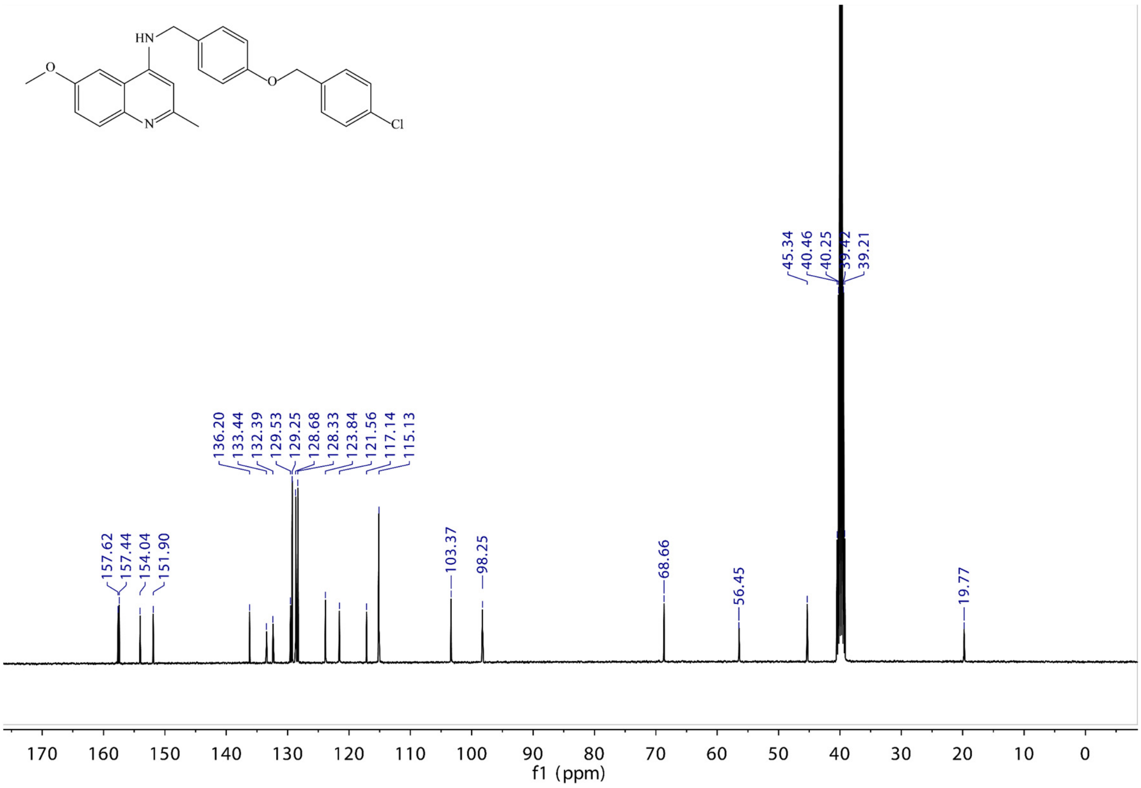

Figure S44: ^{13}C spectrum of **9v** in DMSO-*d6*.

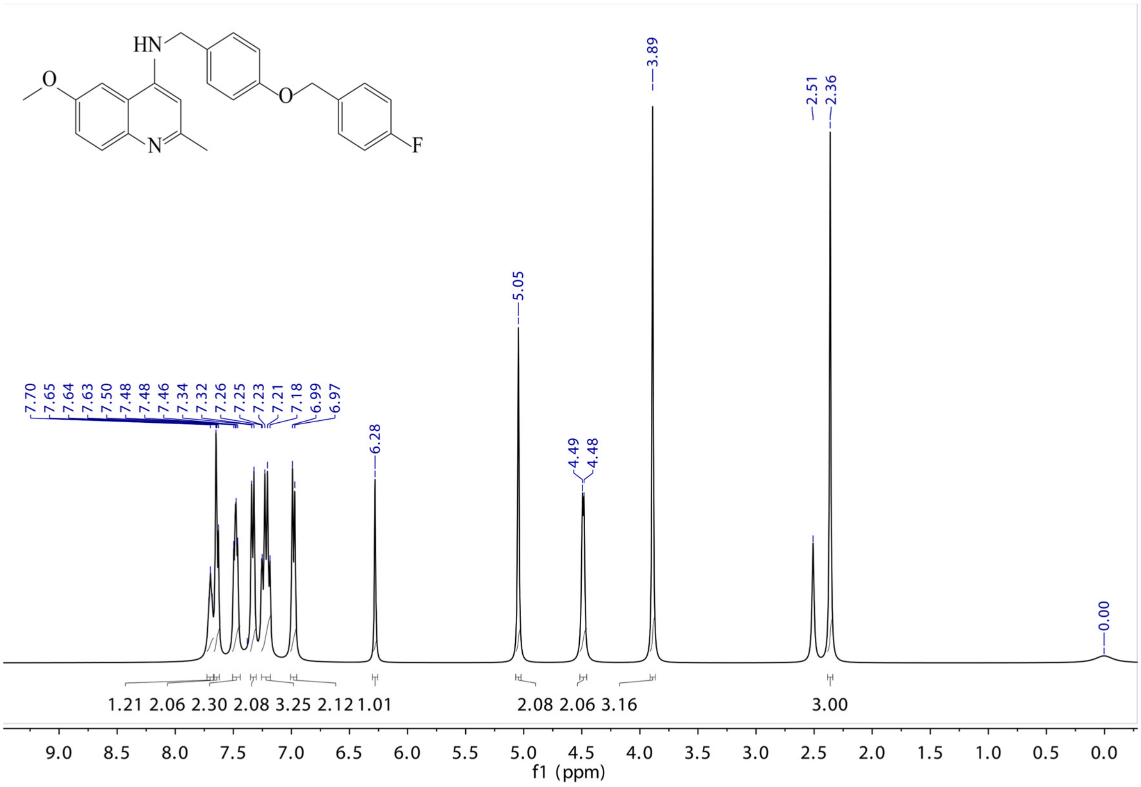

Figure S45: ^{1}H spectrum of **9x** in DMSO-*d₆*.

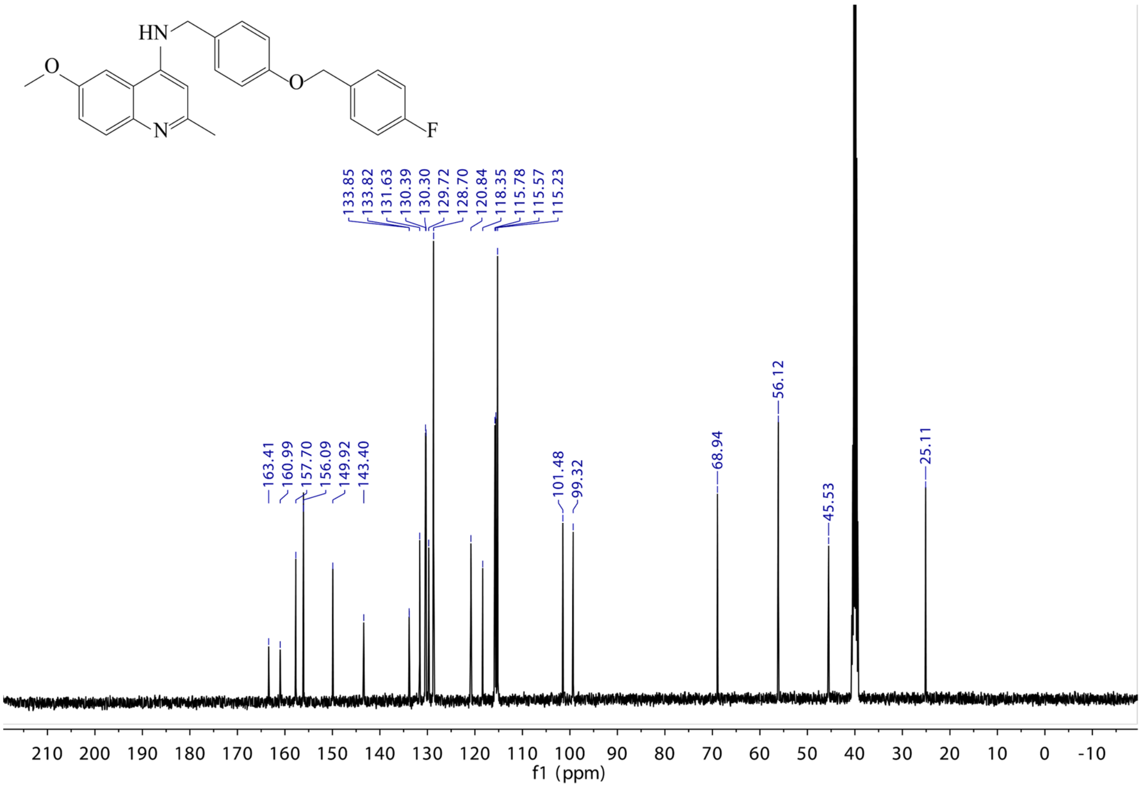

Figure S46: ^{13}C spectrum of **9x** in DMSO-d_6.

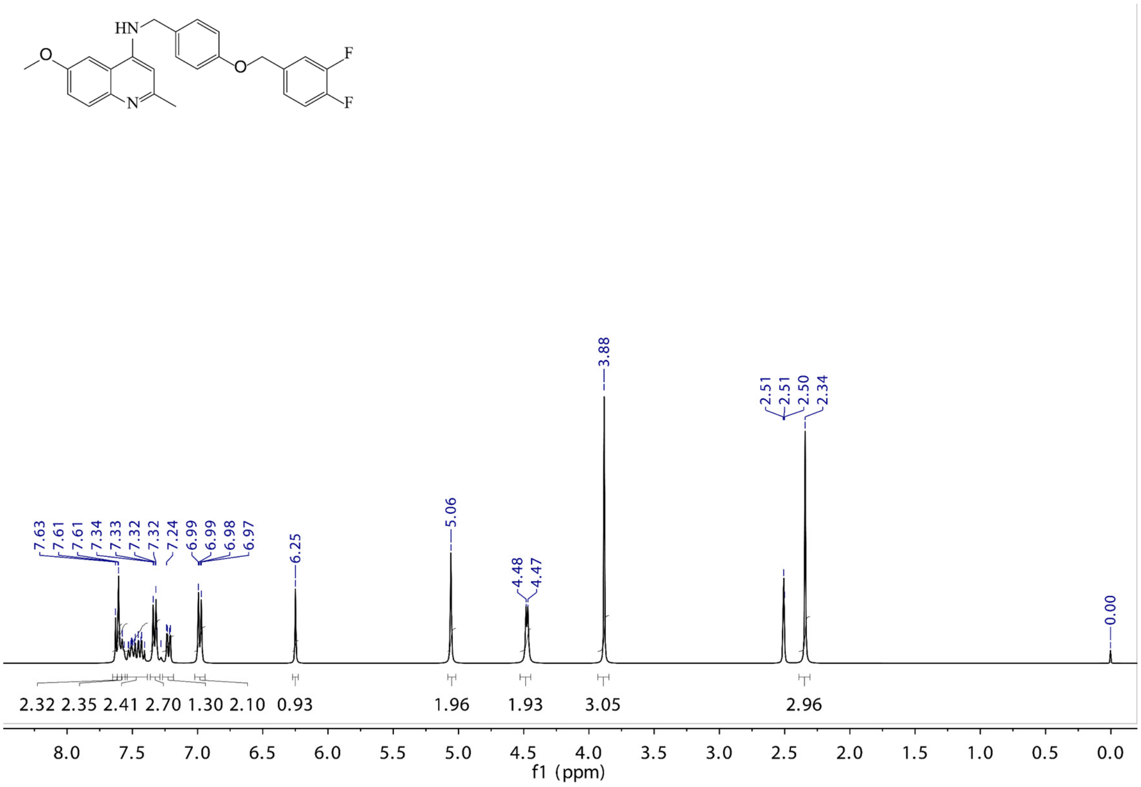

Figure S47: ^{1}H spectrum of **9y** in DMSO-d_6.

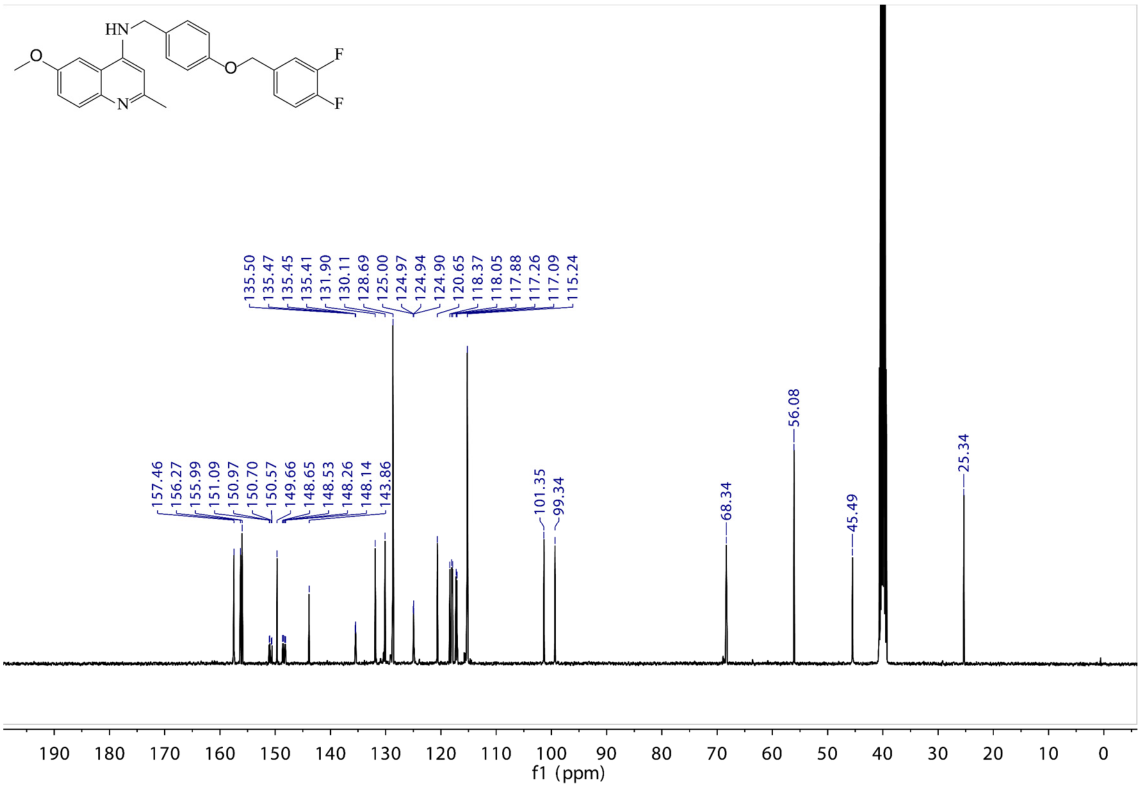

Figure S48: ^{13}C spectrum of **9y** in DMSO-d_6.

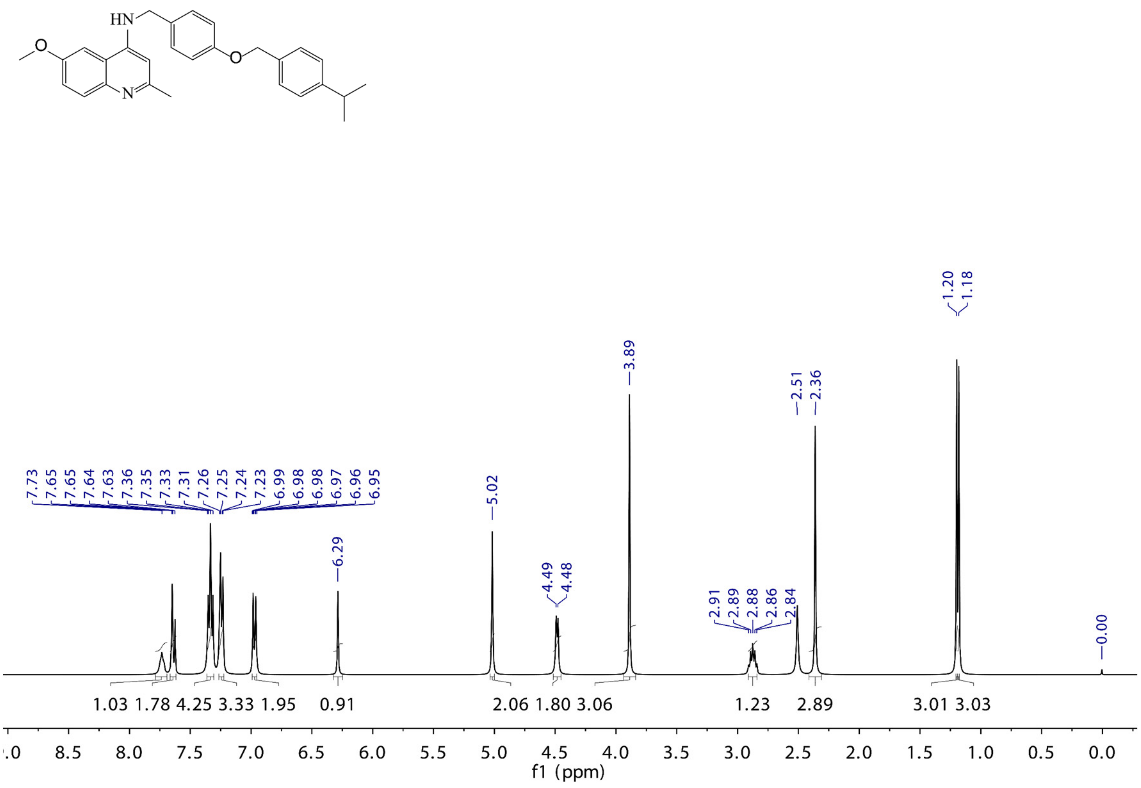

Figure S49: ^{1}H spectrum of **9w** in DMSO-d_6.

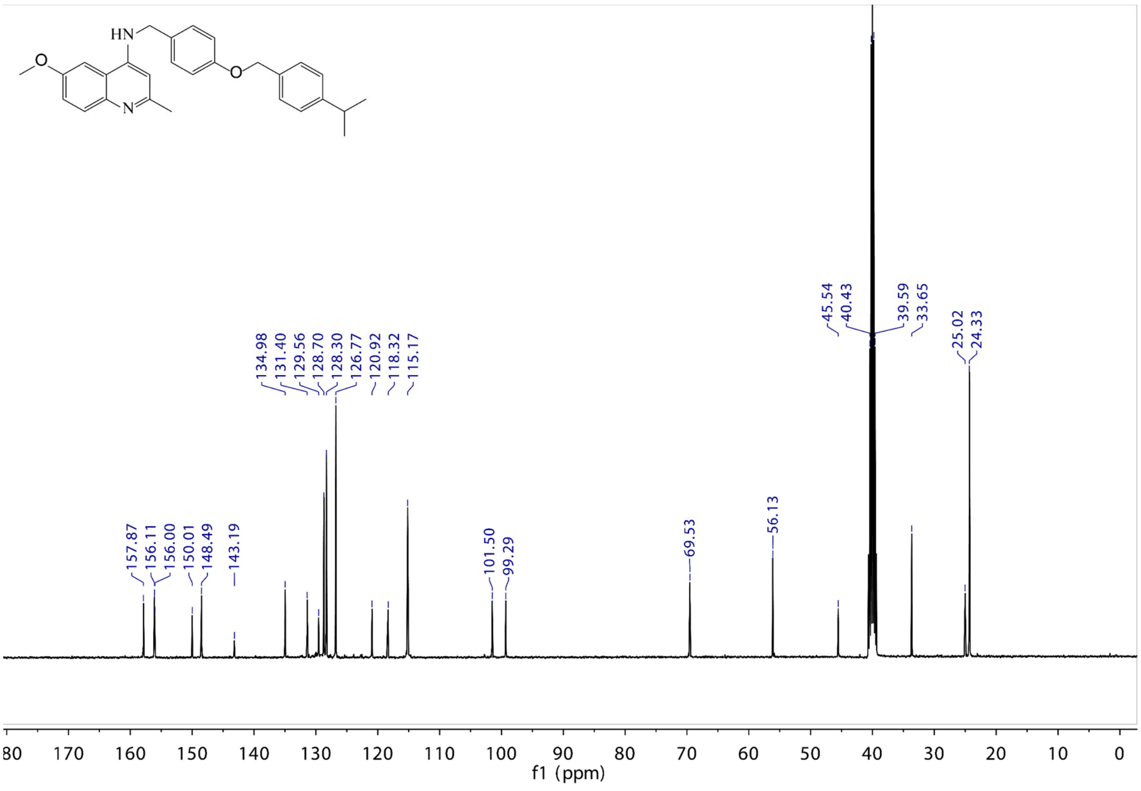

Figure S50: ^{13}C spectrum of **9w** in DMSO-d_6.

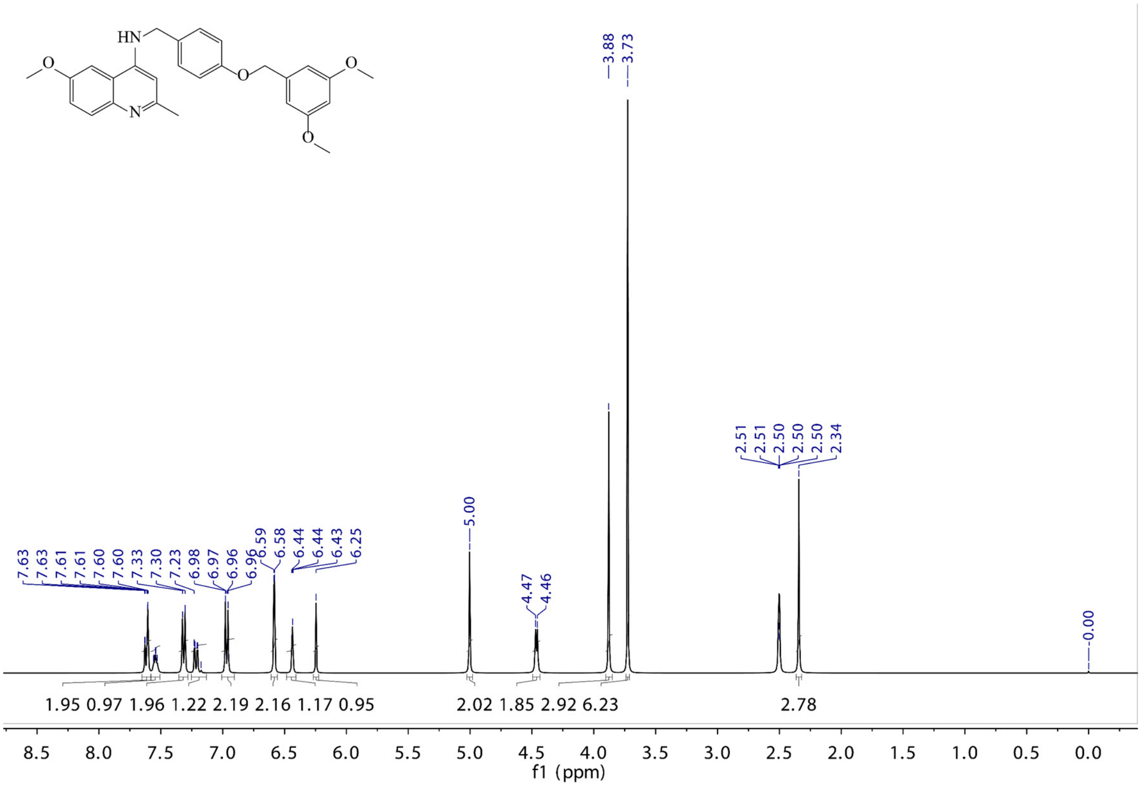

Figure S51: ^{1}H spectrum of **9z** in DMSO-d_6.

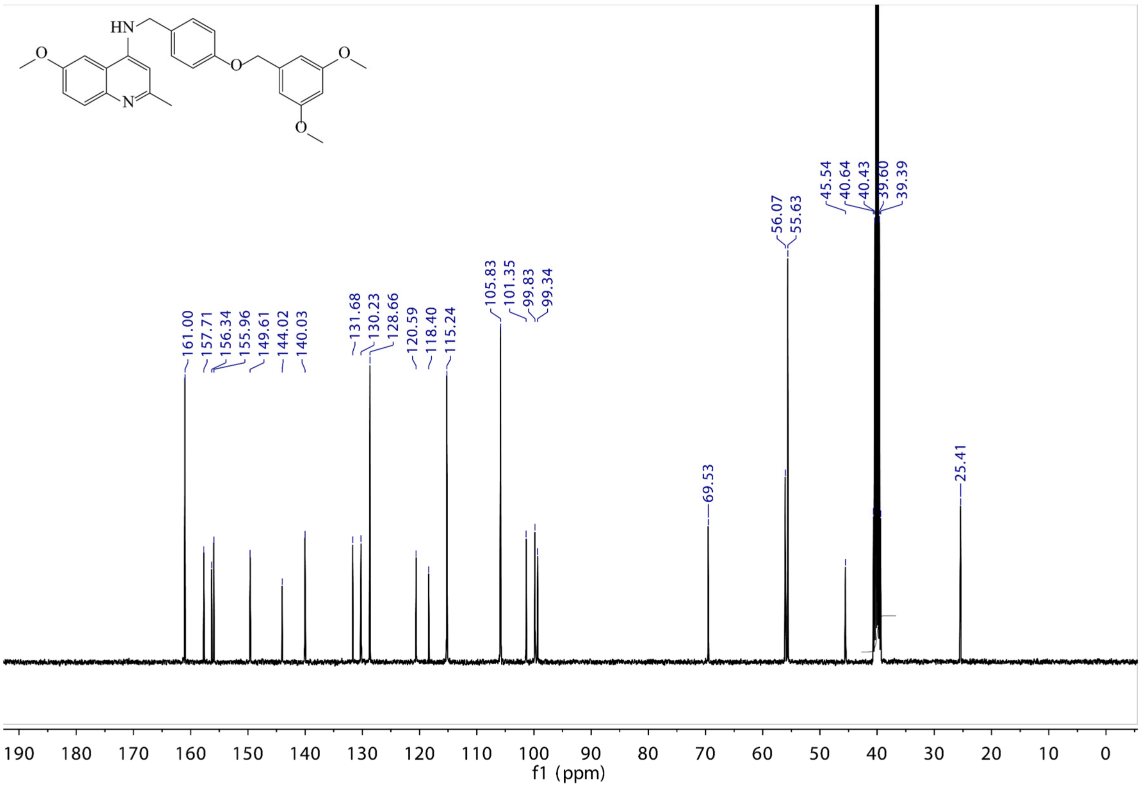

Figure S52: ^{13}C spectrum of **9z** in DMSO-d_6.

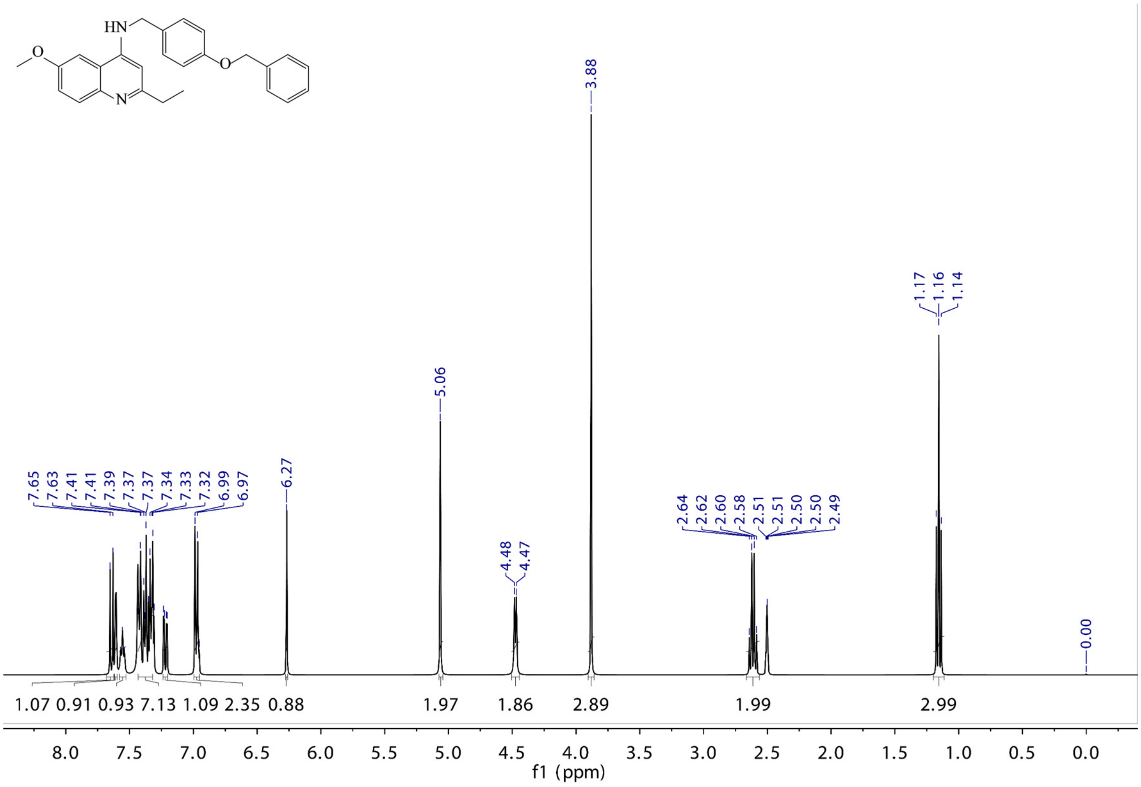

Figure S53: ^{1}H spectrum of **9aa** in DMSO-d_6.

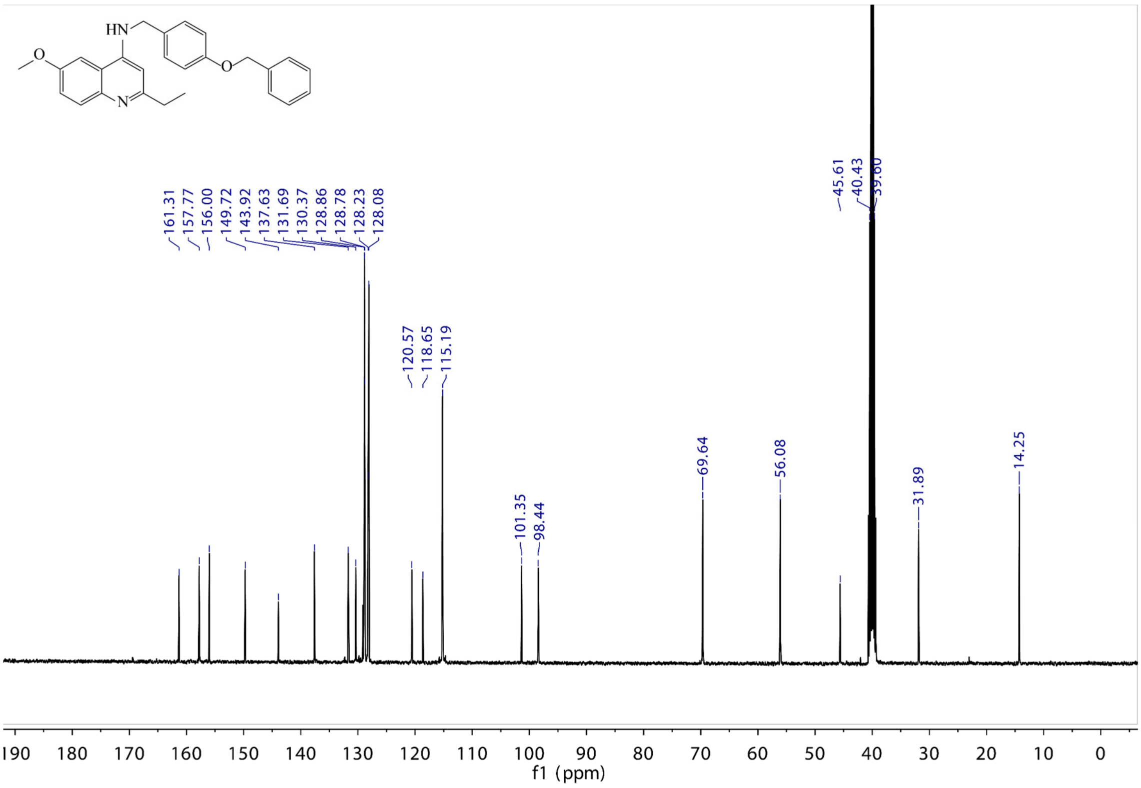

Figure S54: ^{13}C spectrum of **9aa** in DMSO-d_6.

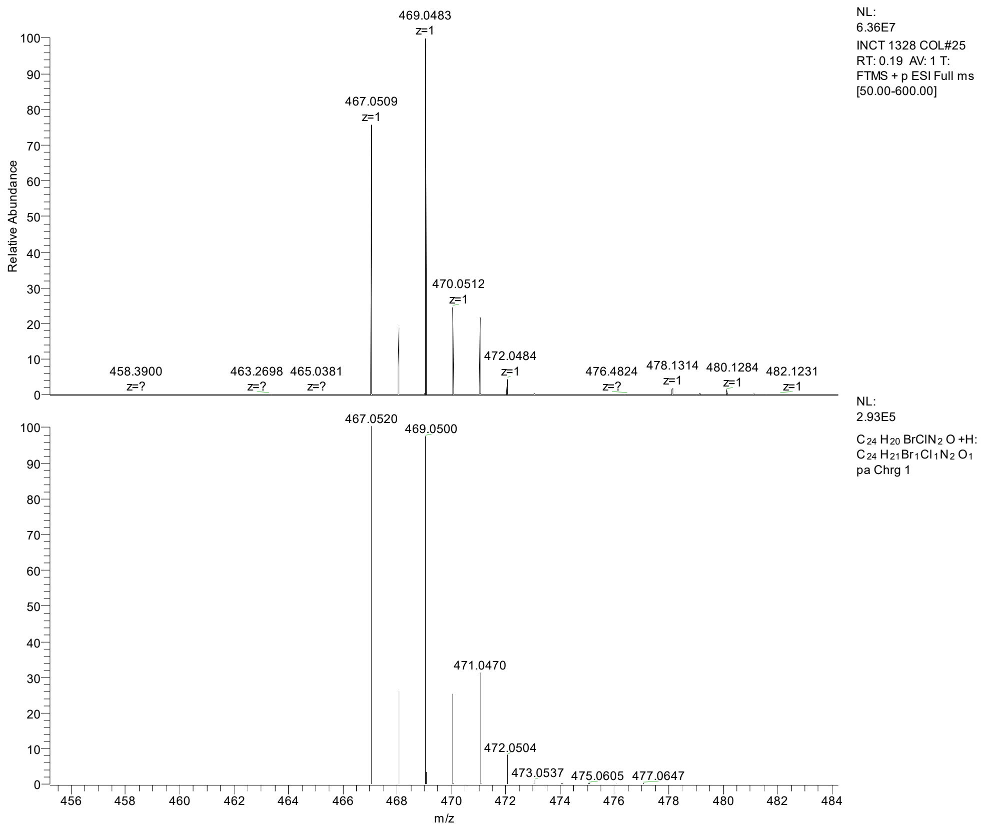

Figure S55: Mass spectrum of **9n**.

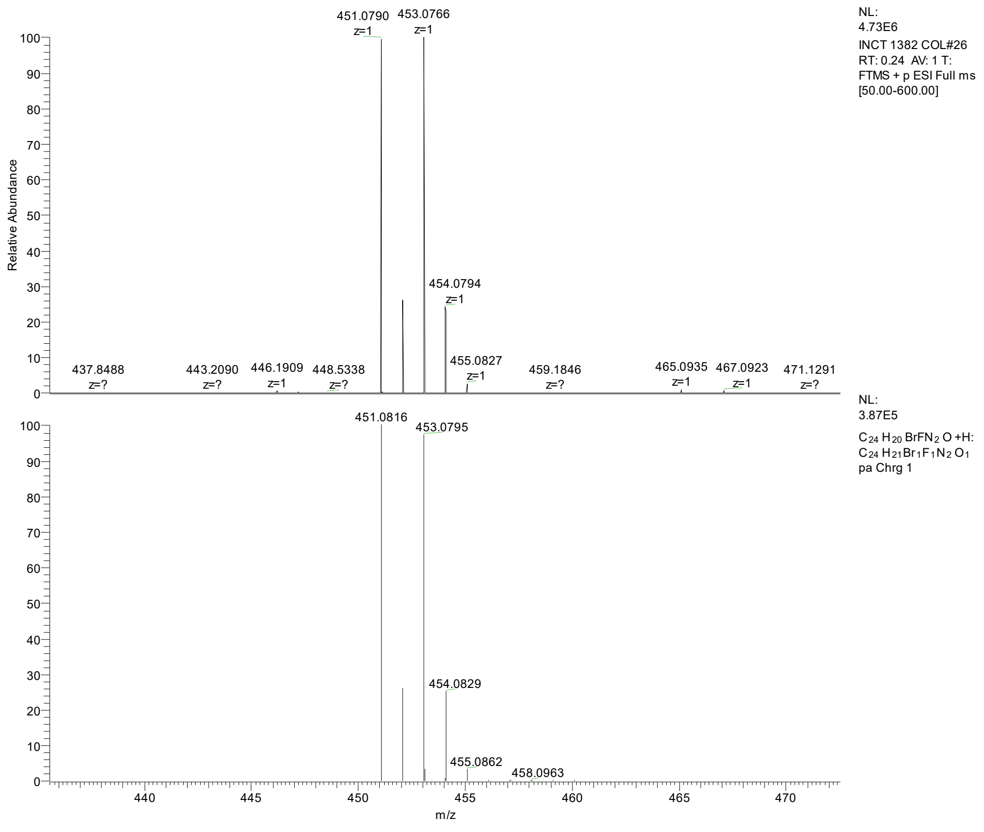

Figure S56: Mass spectrum of **9o**.
